# Glutamine suppresses senescence and promotes autophagy through glycolysis inhibition-mediated AMPKα lactylation in intervertebral disc degeneration

**DOI:** 10.1038/s42003-024-06000-3

**Published:** 2024-03-14

**Authors:** Yangyang Zhang, Zhengqi Huang, Weitao Han, Jiajun Wu, Shuangxing Li, Tianyu Qin, Chao Zhang, Ming Shi, Shun Han, Bo Gao, Song Jin, Yin Xiao, Kang Xu, Wei Ye

**Affiliations:** 1 Department of Spine Surgery, the First Affiliated Hospital of University of South China, Hengyang, 421200 China; 2 Department of Spine Surgery, Sun Yat-sen Memorial Hospital of Sun Yat-sen University, Guangzhou, 510289 China; 3 Guangdong Provincial Key Laboratory of Malignant Tumor Epigenetics and Gene Regulation, Medical Research Center, Sun Yat-sen Memorial Hospital, Sun Yat-sen University, Guangzhou, 510289 China; 4 Department of Orthopedics, the Eighth Affiliated Hospital of Sun Yat-sen University, Shenzhen, 518031 China; 5 School of Medicine and Dentistry, Menzies Health Institute Queensland, Griffith University, Brisbane, QLD Australia

**Keywords:** Senescence, Diseases

## Abstract

Regulating metabolic disorders has become a promising focus in treating intervertebral disc degeneration (IDD). A few drugs regulating metabolism, such as atorvastatin, metformin, and melatonin, show positive effects in treating IDD. Glutamine participates in multiple metabolic processes, including glutaminolysis and glycolysis; however, its impact on IDD is unclear. The current study reveals that glutamine levels are decreased in severely degenerated human nucleus pulposus (NP) tissues and aging Sprague-Dawley (SD) rat nucleus pulposus tissues, while lactate accumulation and lactylation are increased. Supplementary glutamine suppresses glycolysis and reduces lactate production, which downregulates adenosine-5’-monophosphate-activated protein kinase α (AMPKα) lactylation and upregulates AMPKα phosphorylation. Moreover, glutamine treatment reduces NP cell senescence and enhances autophagy and matrix synthesis via inhibition of glycolysis and AMPK lactylation, and glycolysis inhibition suppresses lactylation. Our results indicate that glutamine could prevent IDD by glycolysis inhibition-decreased AMPKα lactylation, which promotes autophagy and suppresses NP cell senescence.

## Abstract (web summary)

Glutamine could prevent IDD by glycolysis inhibition through decreased AMPKα lactylation, which promotes autophagy and suppresses NP cell senescence.

## Introduction

Low back pain (LBP) has emerged as a global public health problem and has a prevalence rate of 18.3% worldwide, leading to substantial disability and medical costs^1–3^. Intervertebral disc degeneration (IDD) is an important cause of LBP^4,5^. As IDD progresses, the anabolism of extracellular matrix (ECM) components, including aggrecan (ACAN) and type II collagen (COL2A1), is suppressed, whereas the catabolism of ECM components, such as a disintegrin-like and metalloprotease with thrombospondin type-1 motif (ADAMTSs) and matrix metalloproteinases (MMPs), is increased in nucleus pulposus (NP)^6^. Moreover, cell senescence and programmed cell death, such as autophagy, representing the arrest of the cell cycle and considered a hallmark of aging, were disturbed^7–9^. Several factors, including inflammatory cytokines, mechanical overloading, aging, and oxidative stress, accumulate over a long period and regulate the biological function of intervertebral disc cells, ultimately accelerating IDD^10^. Recently, the regulation of metabolic disorders has become a promising and encouraging strategy for the treatment of IDD^11^. Obesity and type 2 diabetes have been shown to play essential roles in the development of IDD^12^. Several studies have shown the involvement of metabolites, including cholesterol, fatty acids, lactate, and homocysteine^13–16^, as well as metabolic reprogramming^16^, in the biological function of nucleus pulposus (NP) cells. Moreover, previous studies have shown that drugs such as atorvastatin, metformin, and melatonin regulate metabolism in NP and can effectively inhibit IDD^17–19^. Therefore, it is necessary to assess the effects of changes in metabolites and metabolic pathways in IDD.

Glutamine, which is a metabolic substrate, participates in multiple metabolic processes as a carbon source and nitrogen donor for the synthesis of nucleotides, amino acids, and lipids, as well as the tricarboxylic acid (TCA) cycle^20^. It is not only an in vivo metabolite but also a common clinical drug^21^. Moreover, it is the most abundant amino acid in human serum^21^. Glutamine is closely related to the occurrence and development of metabolic diseases, such as type 2 diabetes and obesity^22,23^, and can promote bone and cartilage development and repair^24–26^. However, the correlation between glutamine and IDD is still unclear.

Glycolysis is the main mode of energy metabolism in NP cells, and lactate is the end product of glycolysis, which is an essential factor in IDD^27,28^. In several diseases, glutamine can cause metabolic reprogramming, including glutaminolysis and glycolysis^22,29,30^. In adipose cells, glutamine inhibited glycolysis to decrease the production of uridine-5-diphospho-n-acetylglucosamine (UDP-GlcNAc) and restricted the O-GlcNAcylation of SP1 to decrease inflammation^22^. Whether glutamine modulates glycolysis in NP cells remains elusive. In addition, loss of the lactate transporter MCT4 could induce lactate accumulation, causing an imbalance in intracellular pH homeostasis and disc degeneration^31^. Extracellular lactate accumulation could increase reactive oxygen species (ROS) levels and pyroptosis in NP cells through acid-sensing ion channels^15^. Whether glutamine inhibits IDD by reducing lactate accumulation is uncertain. In addition, gene lactylation is widely involved in diseases, such as Alzheimer’s disease^32–34^; however, it has not been reported in IDD.

In the current study, we demonstrated the effect of glutamine on cell autophagy and senescence in NP and the role of glycolysis, lactate accumulation, and gene lactylation in the above process. Evidence that glutamine is beneficial in NP cells provides insights into the pathogenesis, prevention, and treatment of IDD.

## Results

### Glutamine levels were closely associated with IDD

**Fig. 1:**
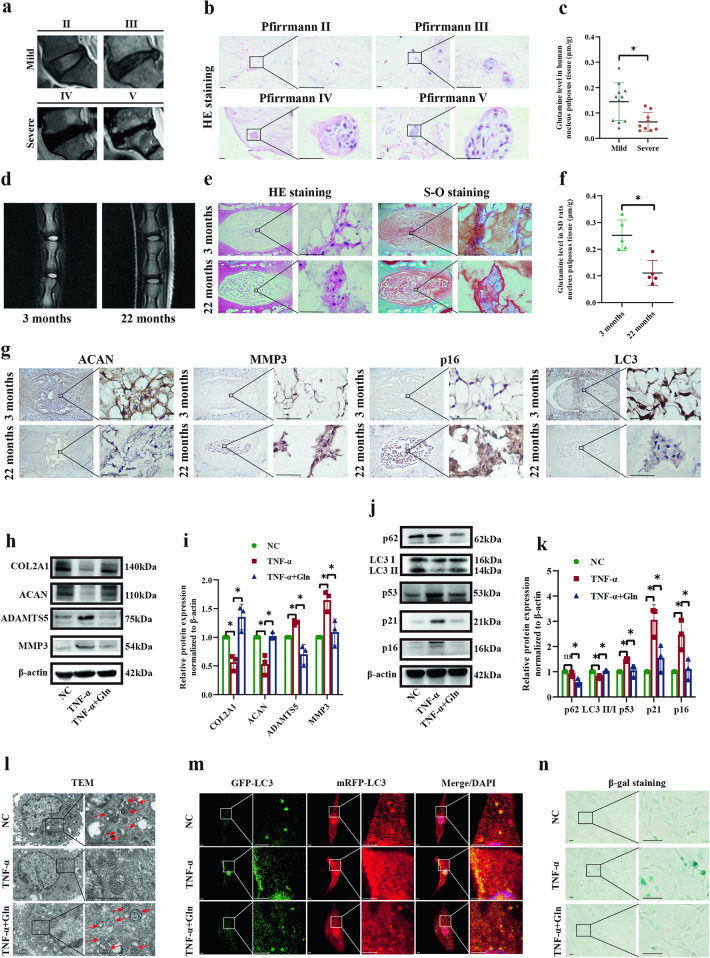
Glutamine levels were closely associated with IDD. **a** MRI scans of mildly and severely degenerated human NP tissues (mild: Pfirrmann grades II and III, severe: Pfirrmann grades IV and V). **b** Hematoxylin-eosin (HE) and Safranin O and Fast Green (S-O) staining of mildly and severely degenerated human NP tissues. Scale bar = 500 μm. **c** Glutamine levels of mildly (*n* = 11) and severely degenerated (*n* = 9) NP tissues. **d** MRI scans of NP tissues from 3-month-old and 22-month-old SD rats. **e** HE and S-O staining of NP tissues from 3-month-old and 22-month-old SD rats. Scale bar = 500 μm. **f** Glutamine levels in NP tissues from 3-month-old (*n* = 5) and 22-month-old (*n* = 5) SD rats. **g** IHC staining of ACAN, MMP3, p16, and LC3 expression in NP tissues from 3-month-old and 22-month-old SD rats. Scale bar = 500 μm. **h** Western blot analysis showing the protein expression of COL2A1, ACAN, ADAMTS5, and MMP3 in SD rat NP cells stimulated with TNF-α and glutamine. **i** Semiquantitative analysis and statistical analysis of the Western blots in (**h**). **j** Western blot analysis showing the protein expression of p62, LC3 II/I, p53, p21, and p16 in SD rat NP cells stimulated with TNF-α and glutamine. **k** Semiquantitative analysis and statistical analysis of the Western blots in (**j**). **l** TEM images showing autolysosome-like vesicles in SD rat NP cells stimulated with TNF-α and glutamine. Scale bar = 2 μm. **m** Yellow spots (GFP^+^mRFP^+^) and red spots (GFP^-^mRFP^+^) indicate autophagosomes and autolysosomes, respectively, in SD rat NP cells stimulated with TNF-α and glutamine. Scale bar = 2 μm. **n** β-gal staining showed senescent cells stimulated by TNF-α and glutamine. Scale bar = 100 μm. ^*^p < 0.05.

To investigate the clinical relevance of glutamine in IDD, eleven mildly degenerated and nine severely degenerated NP tissues based on the Pfirrmann classification were collected. A lower T2 signal of the discs of the lumbar vertebra (Fig. 1a) and more aggregation of the NP cells were discovered in the severely degenerated group than in the mildly degenerated group (Fig. 1b). The global glutamine levels in severely degenerated human NP tissues were significantly lower than those in mildly degenerated human NP tissues (Fig. 1c, *p* = 0.0095). In degenerated NP tissues in the aging rat model (Fig. 1d–g), we also detected lower glutamine levels (Fig. 1f, *p* = 0.0027). The 22-month-old rats showed a lower T2 signal of the disc of the caudal vertebra (Fig. 1d) and decreased cell numbers with more aberrant clusters of NP cells (Fig. 1e) compared with the 3-month-old rats. Moreover, there were meaningful decreases in the expression levels of ACAN and LC3 in 22-month-old rats, while MMP3 and p16 were markedly enhanced, suggesting IDD in 22-month-old SD rats (Fig. 1g).

Next, we evaluated the effects of glutamine on cellular matrix synthesis, autophagy, and senescence in an SD rat NP cell model treated with TNF-α. NP cells treated with TNF-α showed a significant decrease in matrix synthesis-associated protein levels (COL2A1 and ACAN) and an increase in cellular matrix degradation-associated proteins (ADAMTS5 and MMP3), and these effects were reversed by the addition of glutamine (Fig. 1h, i). In addition, glutamine attenuated the increase in the expression levels of senescence-associated proteins (p53, p21, and p16) and induced autophagy at the protein level (p62, LC3 II/I) in the TNF-α group (Fig. 1j, k).

Moreover, we detected the rescue effect of glutamine on autophagy using TEM and mRFP-GFP-LC3 fluorescent spots. TEM showed significantly fewer autolysosomes in the TNF-α-treated cells than in the controls, whereas glutamine-induced significantly more autolysosomes in the TNF-α group (Fig. 1l). We transfected NP cells with the mRFP-GFP-LC3 virus to determine their autophagic capability and quantified the numbers of autophagosomes (merge, yellow spots, GFP^+^mRFP^+^) and autolysosomes (merge, red spots, GFP^-^mRFP^+^). The numbers of autophagosomes and autolysosomes were significantly decreased in NP cells after TNF-α treatment compared to the controls, and these effects could be reversed by glutamine treatment (Fig. 1m). SA-β-gal staining demonstrated a marked increase in SA-β-gal-positive cells in the TNF-α group, and this effect was profoundly reversed by glutamine treatment (Fig. 1n). These results indicate that glutamine can enhance autophagy and cellular matrix synthesis and inhibit senescence.

### Glutamine-induced glycolysis inhibition, which promoted cellular matrix synthesis and autophagy and reduced senescence

**Fig. 2:**
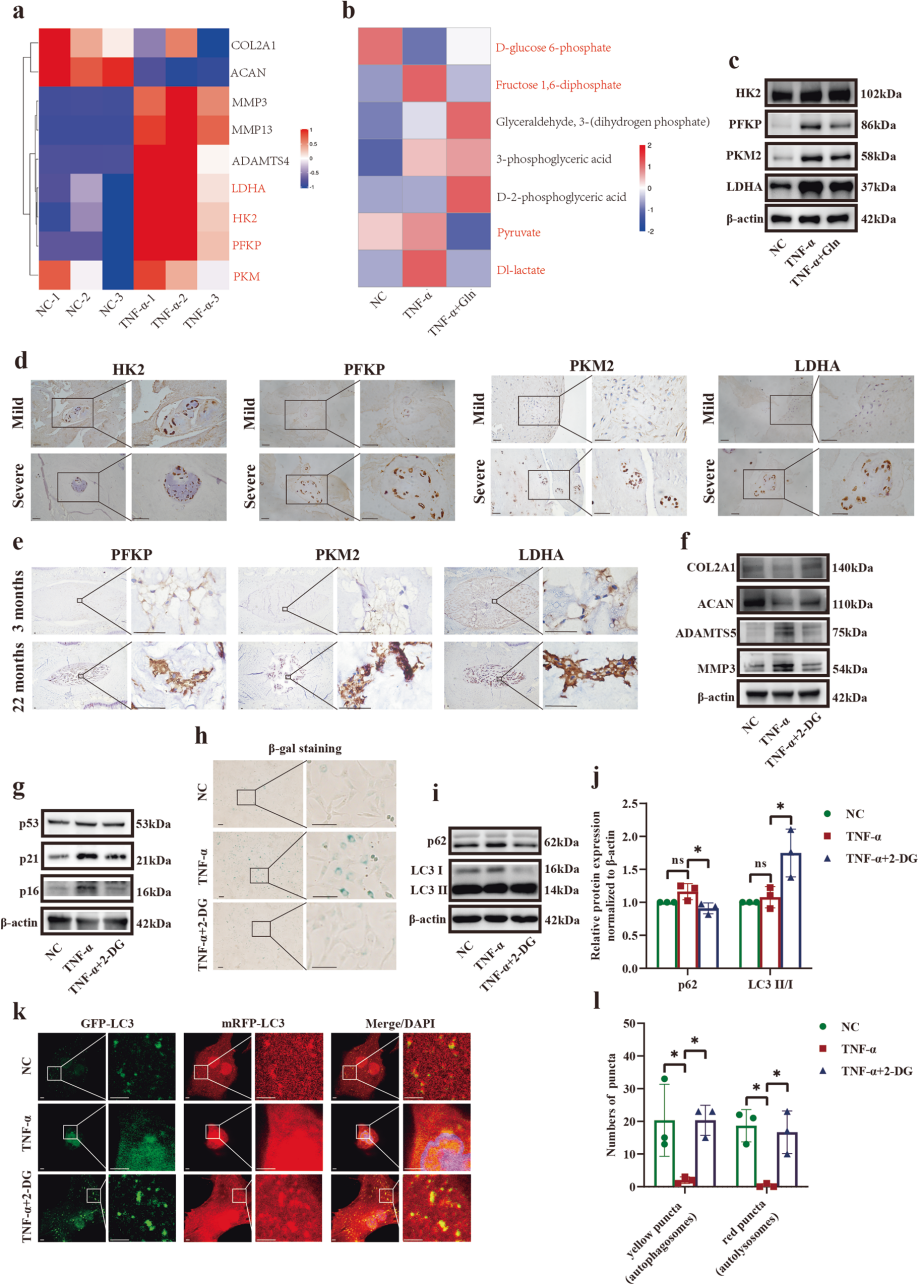
Glutamine-induced glycolysis inhibition, which promoted cellular matrix synthesis and autophagy and reduced senescence. **a** Transcriptomics analysis showing changes in key glycolysis enzymes in human NP cells in response to TNF-α and glutamine stimulation. **b** Metabolomics analysis showing changes in the intermediate products of glycolysis in human NP cells stimulated with TNF-α and glutamine. The scale bar in (**a**) and (**b**) represent row minima/maxima of enrichment levels. **c** Western blot analysis showing the protein expression of HK2, PFKP, PKM2, and LDHA in SD rat NP cells stimulated with TNF-α and glutamine. **d** IHC staining of HK2, PFKP, PKM2, and LDHA expression in mildly and severely degenerated human NP tissues. Scale bar = 500 μm. **e** IHC staining of PFKP, PKM2, and LDHA expression in NP tissues from 3-month-old and 22-month-old SD rats. **f** Western blot analysis showing the protein expression of COL2A1, ACAN, ADAMTS5, and MMP3 in SD rat NP cells stimulated with TNF-α and the glycolysis inhibitor 2-DG. **g** Western blot analysis showing the protein expression of p53, p21, and p16 in SD rat NP cells in response to stimulation with TNF-α and 2-DG. **h** β-gal staining showing the level of senescence in SD rat NP cells in response to stimulation with TNF-α and 2-DG. Scale bar = 100 μm. **i** Western blot analysis showing the protein expression of p62 and LC3 II/I in SD rat NP cells in response to stimulation with TNF-α and 2-DG. **j** Semiquantitative analysis and statistical analysis of the Western blots in (**i**). **k** Yellow spots (GFP^+^mRFP^+^, autophagosomes) and red spots (GFP^-^mRFP^+^, autolysosomes) show autophagosomes and autolysosomes in SD rat NP cells in response to stimulation with TNF-α and 2-DG. Scale bar = 2 μm. **l** Statistical analysis showing the number of autophagosomes and autolysosomes in (**k**). ^*^p < 0.05.

To explore whether glutamine inhibited glycolysis, we used transcriptomics and metabolomics to examine the key enzymes involved in glycolysis. Our results (GEO: GSE226186) showed that HK2, PFKP, and LDHA were significantly increased in TNF-α-induced human NP cells (Fig. 2a, Supplementary Fig. 4). Moreover, TNF-α induced intermediate products corresponding to the key enzymes of glycolysis, such as fructose 1,6-diphosphate (corresponding to PFKP), pyruvate (corresponding to PKM2) and Dl-lactate (corresponding to LDHA) but not D-glucose 6-phosphate (corresponding to HK2), whereas glutamine treatment reversed the effect of TNF-α (Fig. 2b, Supplementary Fig. 5). The expression of PFKP, PKM2, and LDHA was significantly increased by TNF-α treatment and alleviated by glutamine (Fig. 2c). Correspondingly, in the NP tissues of the severely degenerated group and 22-month-old SD rats, the expression of PFKP, PKM2, and LDHA was markedly enhanced compared to that in mildly degenerated human NP tissues and 3-month-old SD rat NP tissues (Fig. 2d, e).

We next assessed the effect of glycolysis inhibition. We found that glycolysis inhibition by 2-DG markedly heightened the expression of COL2A1 and ACAN and diminished ADAMTS5 and MMP3 expression in TNF-α-treated NP cells (Fig. 2f). Meanwhile, 2-DG treatment neutralized the increase in senescence-associated proteins (p53, p21, and p16) and the increase in the population of SA-β-gal-positive cells induced by TNF-α (Fig. 2g, h). Additionally, we found that 2-DG inhibited the expression of p62 and augmented the LC3 II/I ratio (Fig. 2I, j). The decrease in autophagosomes and autolysosomes in TNF-α-treated NP cells was abrogated by 2-DG (Fig. 2k, l). These results indicate that glycolysis inhibition promotes cellular matrix synthesis and autophagy and reduces senescence.

### The effects of glutamine on NP cells were counteracted by lactate

**Fig. 3:**
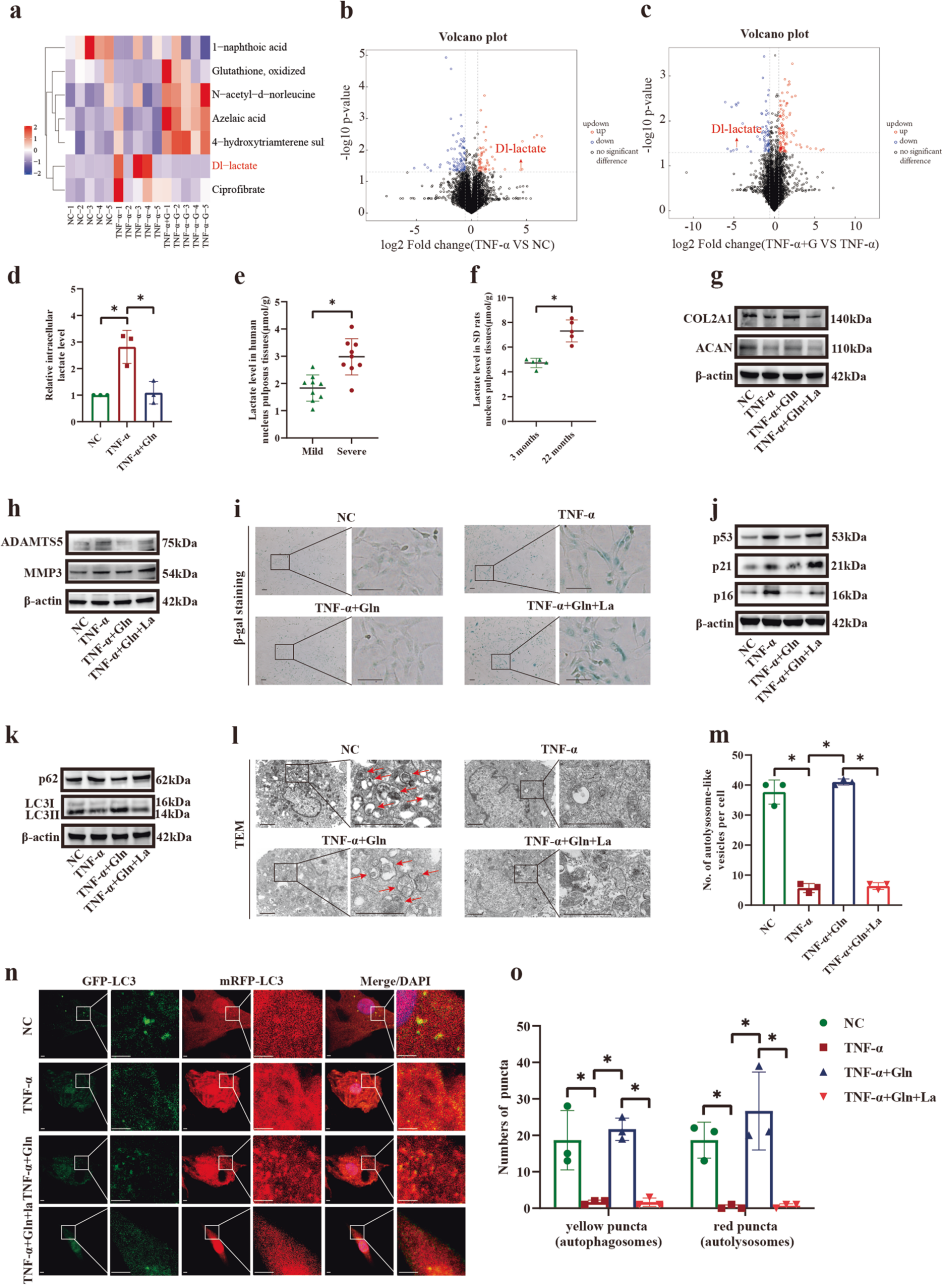
The effects of glutamine on NP cells were counteracted by lactate. **a** Negative ionization mode of hierarchical clustering analysis showing a significant change in metabolites. **b** The negative ionization mode of the volcano plot showing that Dl-lactate changed prominently in response to TNF-α stimulation and control conditions. **c** The negative ionization mode of the volcano plot showing that Dl-lactate changed prominently in response to glutamine and TNF-α. **d** Assessment of lactate levels in response to TNF-α, glutamine in rat NP cells. **e** Assessment of lactate levels in mildly and severely degenerated human NP tissues. **f** Assessment of lactate levels in NP tissues from 3-month-old and 22-month-old SD rats. **g** Western blot analysis showing the protein expression of COL2A1 and ACAN in SD rat NP cells stimulated with TNF-α, glutamine, and lactate. **h** Western blot analysis showing the protein expression of ADAMTS5 and MMP3 in SD rat NP cells stimulated with TNF-α, glutamine, and lactate. **i** β-gal staining showing the level of senescence in SD rat NP cells stimulated with TNF-α, glutamine, and lactate. Scale bar = 100 μm. **j** Western blot analysis showing the protein expression of p53, p21 and p16 in SD rat NP cells stimulated with TNF-α, glutamine, and lactate. **k** Western blot analysis showing the protein expression of p62 and LC3 II/I in SD rat NP cells stimulated with TNF-α, glutamine, and lactate. **l** TEM showing autolysosome-like vesicles in SD rat NP cells stimulated with TNF-α, glutamine, and lactate. Scale bar = 2 μm. **m** Statistical analysis showing the number of autolysosome-like vesicles in (**l**). **n** Yellow spots (GFP^+^mRFP^+^, autophagosomes) and red spots (GFP^-^mRFP^+^, autolysosomes) show autophagosomes and autolysosomes, respectively, in SD rat NP cells stimulated with TNF-α, glutamine, and lactate. Scale bar = 2 μm. **o** Statistical analysis showing the number of autophagosomes and autolysosomes in (**n**). ^*^p < 0.05.

To explore the polar metabolites involved in the regulatory effect of glutamine on human NP cells, nontargeted metabolomics was performed to detect changes in the metabolic microenvironment. The results showed the proportion of identified metabolites in each chemical classification (Supplementary Fig. 6a). In the negative ionization mode, the expression levels of 1-naphthoic acid, glutathione and oxidized, N-acetyl-d-norleucine, azelaic acid, 4-hydroxytriamterene sulfate, Dl-lactate, and ciprofibrate were significantly changed (Fig. 3a, Supplementary Fig. 7). Among these, only Dl-lactate was significantly increased by TNF-α and simultaneously reversed by glutamine (VIP > 1, −0.58 < log_2_Fold change < 0.58, *p* < 0.05; Fig. 3b, c). Some polar metabolites were significantly different after stimulation with TNF-α or glutamine in the positive ionization mode (Supplementary Fig. 6b and Supplementary Fig. 8). However, those metabolites were not changed simultaneously between the TNF-α and glutamine groups (VIP > 1, −0.58 < log_2_Fold change < 0.58, *p* < 0.05; Supplementary Fig. 6b–d). Then, the altered polar metabolite Dl-lactate was assessed for further study. Besides, the concentrations of L-Glutamine and L-Glutamate were decreased under the stimulation of TNF-α, which were reversed by high-level of glutamine, although these trends were not statistically significant (Supplementary Fig. 3).

We measured lactate levels and found a 2.8-fold increase in the TNF-α group compared to the control group and a 2.6-fold decrease in the glutamine group (Fig. 3d, *p* = 0.0053, *p* = 0.0068). Moreover, our results showed a 1.5-fold increase in the levels of lactate in severely degenerated human NP tissues compared with mildly degenerated human NP tissues (Fig. 3e, *p* = 0.0007). Similarly, the lactate level in the NP tissues of 22-month-old SD rats exhibited a 1.5-fold increase compared to that in 3-month-old SD rats (Fig. 3f, *p* = 0.0003). We also found that lactate could inhibit the effect of glutamine in vitro. Lactate significantly decreased the protein expression of COL2A1 and ACAN and significantly elevated the protein expression of ADAMTS5 and MMP3 compared with glutamine (Fig. 3g, h). Moreover, lactate treatment in the glutamine group markedly increased the SA-β-gal-positive cell population (Fig. 3i), the expression levels of senescence-associated proteins (p53, p21, and p16) (Fig. 3j), and the expression of autophagy markers (increased p62 expression and decreased ratio of LC3 II/I) (Fig. 3k) and significantly inhibited the production of autolysosomes (Fig. 3l, m). Meanwhile, the increased number of autophagosomes and autolysosomes in NP cells induced by glutamine treatment was reversed by lactate treatment (Fig. 3n, o). These results indicate that lactate markedly counteracts the protective effect of glutamine.

### Both glutamine and glycolysis inhibition suppressed lactylation, which was increased in IDD

**Fig. 4:**
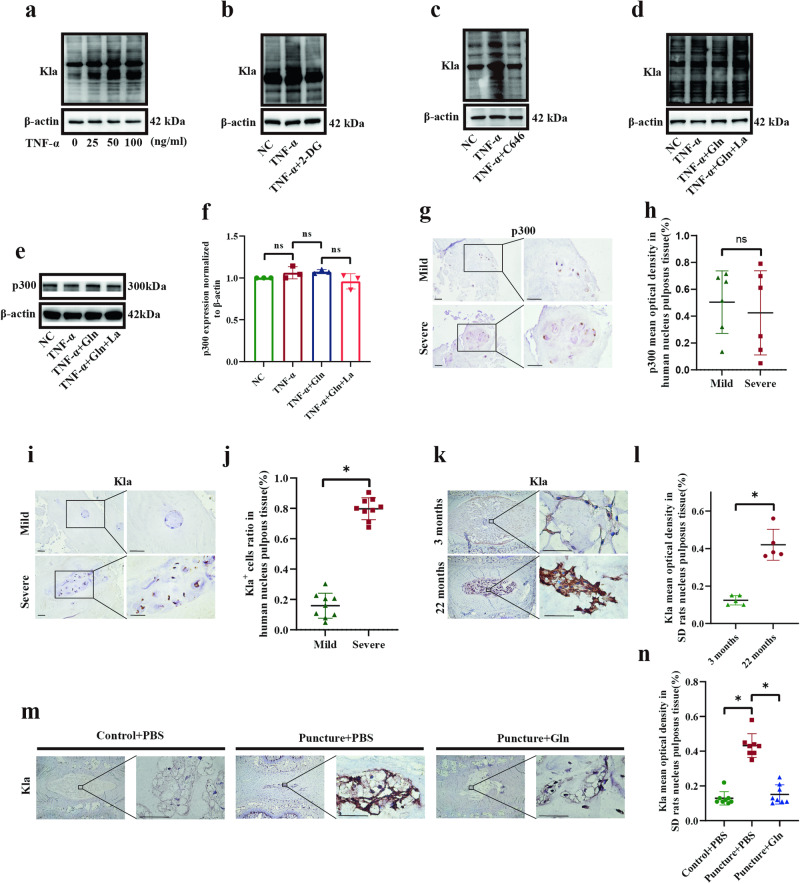
Both glutamine and glycolysis inhibition suppressed lactylation, which was increased in IDD. **a** Western blot analysis showing the protein expression of lactylation-Kla in SD rat NP cells in response to TNF-α stimulation. **b** Western blot analysis showing the protein expression of lactylation-Kla in SD rat NP cells in response to stimulation with TNF-α and 2-DG. **c** Western blot analysis showing the protein expression of lactylation-Kla in SD rat NP cells in response to stimulation with TNF-α and C646. **d** Western blot analysis showing the protein expression of lactylation-Kla in SD rat NP cells in response to stimulation with TNF-α, glutamine and lactate. **e** Western blot analysis showing the protein expression of lactylation transferase p300 in SD rat NP cells in response to stimulation with TNF-α, glutamine and lactate. **f** Semiquantitative analysis and statistical analysis of the Western blots in (**e**). **g** IHC staining of p300 expression in mildly and severely degenerated human NP tissues. Scale bar = 500 μm. **h** Semiquantitative analysis and statistical analysis of IHC in (**g**). **i** IHC staining of lactylation expression in mildly and severely degenerated human NP tissues. Scale bar = 500 μm. **j** Semiquantitative analysis and statistical analysis of IHC in (**i**). **k** IHC staining of lactylation in NP tissues from 3-month-old and 22-month-old SD rats. Scale bar = 500 μm. **l** Semiquantitative analysis and statistical analysis of IHC in (**k**). **m** IHC staining of lactylation in SD rat NP tissues after daily injection of PBS or glutamine. Scale bar = 500 μm. **n** Semiquantitative analysis and statistical analysis of IHC in (**m**). ^*^p < 0.05. (Kla was the symbol of lactylation).

We next examined the effect of TNF-α and glutamine on lactylation. TNF-α markedly increased lactylation (Fig. 4a), whereas a glycolysis inhibitor (2-DG) and a lactylation transferase p300 inhibitor (C646) inhibited this effect (Fig. 4b, c). In addition, the degree of lactylation decreased after glutamine treatment and was increased by lactate treatment (Fig. 4d). The level of lactylation transferase p300 was not significantly altered by TNF-α, glutamine, or lactate (Fig. 4e, f). The level of p300 was also unaltered in severely degenerated and mildly degenerated human NP tissues (Fig. 4g, h, *p* = 0.6272). Lactylation was notably higher in severely degenerated human NP tissues than in mildly degenerated human NP tissues (Fig. 4I, j), and lactylation in the NP tissues of 22-month-old SD rats was markedly higher than that in the NP tissues of 3-month-old SD rats (Fig. 4k, l). In addition, in vivo experiments demonstrated a notable increase in lactylation levels in the NP tissues of the IDD group compared with those in the control group, and this effect was inverted in the glutamine group (Fig. 4m, n). These results show that glutamine and glycolysis inhibition suppress lactylation, which is increased in IDD.

### Lactylation suppression promoted cellular autophagy and inhibited senescence in NP cells

**Fig. 5:**
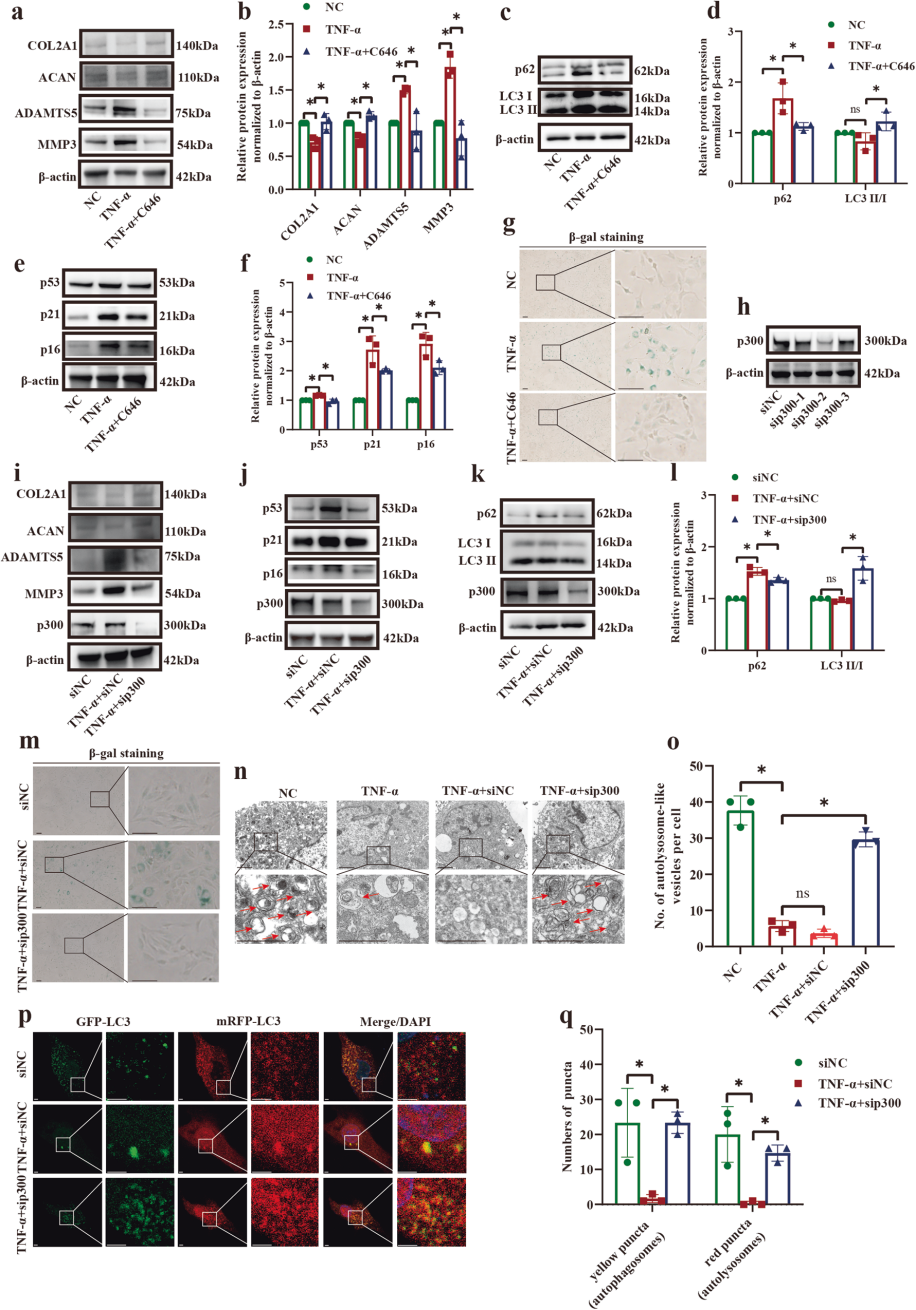
Lactylation inhibition promoted cellular matrix synthesis and autophagy and inhibited senescence. **a** Western blot analysis showing the protein expression of COL2A1, ACAN, ADAMTS5, and MMP3 in SD rat NP cells stimulated with TNF-α and C646. **b** Semiquantitative analysis and statistical analysis of the Western blots in (**a**). **c** Western blot analysis showing the protein expression of p62 and LC3 II/I in SD rat NP cells in response to stimulation with TNF-α and C646. **d** Semiquantitative analysis and statistical analysis of the Western blots in (**c**). **e** Western blot analysis showing the protein expression of p53, p21, and p16 in SD rat NP cells in response to stimulation with TNF-α and C646. **f** Semiquantitative analysis and statistical analysis of the Western blots in (**e**). **g** β-gal staining showing the level of senescence in SD rat NP cells in response to stimulation with TNF-α and C646. Scale bar = 100 μm. **h** Western blot analysis showing the effect of the three sequences of p300 knockdown. **i** Western blot analysis showing the protein expression of COL2A1, ACAN, ADAMTS5, and MMP3 in SD rat NP cells in response to TNF-α stimulation and p300 knockdown. **j** Western blot analysis showing the protein expression of p53, p21, and p16 in SD rat NP cells in response to TNF-α stimulation and p300 knockdown. **k** Western blot analysis showing the protein expression of p62 and LC3 II/I in SD rat NP cells in response to TNF-α stimulation and p300 knockdown. **l** Semiquantitative analysis and statistical analysis of the Western blots in (**k**). **m** β-gal staining showing the level of senescence in SD rat NP cells in response to TNF-α stimulation and p300 knockdown. Scale bar = 100 μm. **n** TEM showing autolysosome-like vesicles in SD rat NP cells in response to TNF-α stimulation and p300 knockdown. Scale bar = 2 μm. **o** Statistical analysis showing the number of autolysosome-like vesicles in (**n**). **p** Yellow spots (GFP^+^mRFP^+^, autophagosomes) and red spots (GFP^-^mRFP^+^, autolysosomes) show autophagosomes and autolysosomes in SD rat NP cells in response to TNF-α stimulation and p300 knockdown. Scale bar = 2 μm. **q** Statistical analysis showing the number of autophagosomes and autolysosomes in (**p**). ^*^p < 0.05.

According to previous results, we evaluated the effects of lactylation on cellular matrix synthesis, autophagy, and senescence. A lactylation inhibitor (C646) significantly enhanced the protein expression of COL2A1 and ACAN and inhibited the protein expression of ADAMTS5 and MMP3 (Fig. 5a, b). In addition, C646 suppressed the protein expression of p62 and increased the protein ratio of LC3 II/I (Fig. 5c, d). C646 also markedly decreased senescence-associated protein expression (p53, p21, and p16) (Fig. 5e, f) and senescence in NP cells (Fig. 5g). Moreover, we used p300 siRNA to downregulate p300 expression in rat NP cells and selected the sequence (sip300-2) with the highest inhibitory effect (Fig. 5h). p300 knockdown increased the expression of COL2A1 and ACAN and decreased the expression of ADAMTS5 and MMP3 (Fig. 5i). Meanwhile, the increase in the expression of senescence-associated proteins was markedly reversed by p300 knockdown (Fig. 5j). In addition, we found that p300 knockdown inhibited the expression of p62 and promoted the LC3 II/I ratio (Fig. 5k, l). β-gal staining showed that p300 knockdown downregulated the level of senescence in rat NP cells stimulated with TNF-α (Fig. 5m). p300 knockdown promoted the production of autolysosomes (Fig. 5n, o) and autophagosomes (Fig. 5p, q) to counteract the effect of TNF-α on NP cells. These results show that inhibiting lactylation promotes cellular matrix synthesis and autophagy and inhibits senescence.

### Glutamine reduced the lactylation of AMPKα and enhanced the phosphorylation of AMPKα

**Fig. 6:**
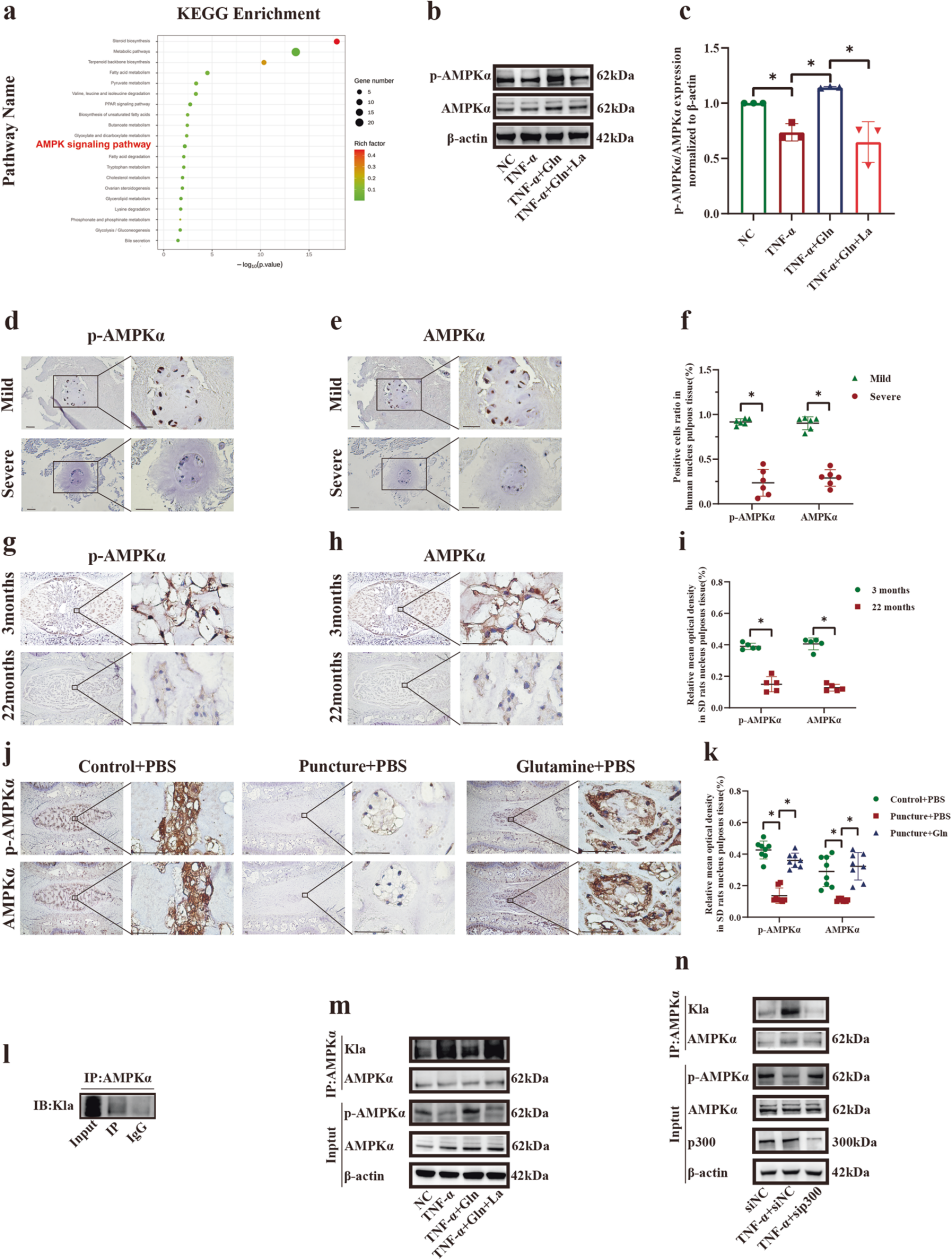
Glutamine reduced the lactylation of AMPKα and enhanced the phosphorylation of AMPKα. **a** KEGG enrichment analysis showing the different pathways in human NP cells in response to TNF-α and glutamine stimulation (TNF-α and TNF-α+glutamine). **b** Western blot analysis showing the protein expression of p-AMPKα/AMPKα in response to stimulation with TNF-α, glutamine, and lactate. **c** Semiquantitative analysis and statistical analysis of the Western blots in (**b**). **d** IHC staining showing the expression of p-AMPKα in mildly and severely degenerated human NP tissues. Scale bar = 500 μm. **e** IHC staining showing the expression of AMPKα in mildly and severely degenerated human NP tissues. Scale bar = 500 μm. **f** Semiquantitative analysis and statistical analysis of IHC in (**d**, **e**). **g** IHC staining showing the expression of p-AMPKα in NP tissues from 3-month-old and 22-month-old SD rats. Scale bar = 500 μm. **h** IHC staining showing the expression of AMPKα in NP tissues from 3-month-old and 22-month-old SD rats. Scale bar = 500 μm. **i** Semiquantitative analysis and statistical analysis of IHC in (**g**, **h**). **j** IHC staining of p-AMPKα and AMPKα expression in SD rat NP tissues after daily injection of PBS or glutamine. Scale bar = 500 μm. **k** Semiquantitative analysis and statistical analysis of IHC in (**j**). **l** Co-IP showing that lactylation interacted with AMPKα. **m** Co-IP showing the protein expression of lactylation-AMPKα/AMPKα and p-AMPKα/AMPKα in response to stimulation with TNF-α, glutamine, and lactate. **n** Co-IP showing the protein expression of lactylation-AMPKα/AMPKα and p-AMPKα/AMPKα in response to TNF-α stimulation and p300 knockdown. ^*^p < 0.05.

KEGG enrichment analysis was conducted to identify the pathways involved in the glutamine response, and the results showed that the AMPK pathway was significantly enriched in this process (Fig. 6a, Supplementary Fig. 2, Supplementary Table 4) and was validated as being upstream of autophagy and senescence. Although most previous studies have confirmed the protective effect of AMPK in IDD^18,35–38^, some studies hold the opposite view^39^. To validate the effect of AICAR in NP cells, we added western blotting to detect the expression of COL2A1, ACAN, ADAMTS5, MMP3, p62, LC3 II/1, and p21 and β-Gal staining to detect the level of senescence. In the results, activating AMPK with AICAR can induce autophagy, inhibit senescence and promote anabolism process, and inhibit catabolism process (Supplementary Fig. 1a–i). In addition, the ratio of p-AMPKα/AMPKα was markedly reduced by TNF-α, and this effect was reversed after glutamine treatment and then decreased following incubation with lactate (Fig. 6b, c, p = 0.0472, *p* = 0.0046, *p* = 0.0013). Furthermore, significantly lower expression of p-AMPKα and AMPKα was observed in severely degenerated human NP tissues than in mildly degenerated tissues (Fig. 6d–f). Correspondingly, the expression levels of p-AMPKα and AMPKα were markedly reduced in 22-month-old SD rat NP tissues compared with 3-month-old SD rat NP tissues (Fig. 6g–i). The in vivo experiments showed significantly lower expression of p-AMPKα and AMPKα in the IDD group than in the control group, and this effect was reversed in the glutamine group (Fig. 6j, k). The coimmunoprecipitation results showed that lactylation was combined with AMPKα (Fig. 6l). Notably, the heightened ratio of lactylation-AMPKα/AMPKα following treatment with TNF-α was markedly reversed after glutamine treatment and enhanced again with lactate (Fig. 6m). Simultaneously, the change in the p-AMPKα/AMPKα level was in contrast to the change in the ratio of lactylation-AMPKα/AMPKα (Fig. 6m). Moreover, the lactylation-AMPKα/AMPKα level was downregulated, while the p-AMPKα/AMPKα level was upregulated after p300 knockdown compared with TNF-α treatment (Fig. 6n). Taken together, the results suggest that glutamine reduced the lactylation of AMPKα and enhanced the phosphorylation of AMPKα.

### Glutamine exerted its effects by activating AMPKα

**Fig. 7:**
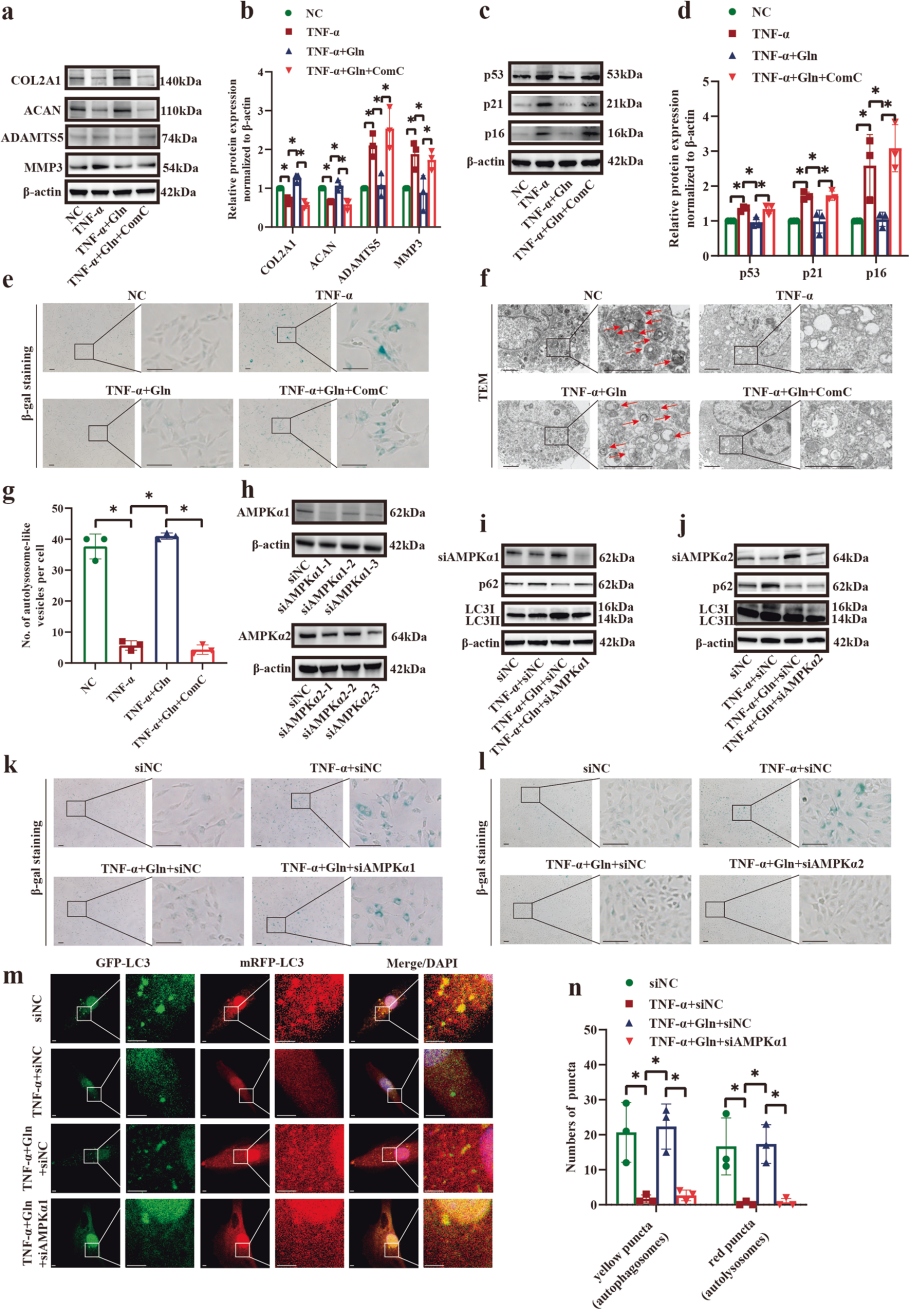
Glutamine exerted its effects by activating AMPKα. **a** Western blot analysis showing the protein expression of COL2A1, ACAN, ADAMTS5, and MMP3 in SD rat NP cells in response to stimulation with TNF-α, glutamine, and compound C. **b** Semiquantitative analysis, and statistical analysis of the Western blots in (**a**). **c** Western blot analysis showing the protein expression of p53, p21, and p16 in SD rat NP cells in response to stimulation with TNF-α, glutamine, and compound C. **d** Semiquantitative analysis and statistical analysis of the Western blots in (**c**). **e** β-gal staining showing the level of senescence in SD rat NP cells in response to stimulation with TNF-α, glutamine and compound C. Scale bar = 100 μm. **f** TEM showing autolysosome-like vesicles in SD rat NP cells in response to stimulation with TNF-α, glutamine, and compound C. Scale bar = 2 μm. **g** Statistical analysis showing the number of autolysosome-like vesicles in (**f**). **h** Western blot analysis showing the effect of the three sequences on AMPKα1 and AMPKα2 knockdown. **i** Western blot analysis showing the protein expression of p62 and LC3 II/I in SD rat NP cells in response to TNF-α stimulation, glutamine, and AMPKα1 knockdown. **j** Western blot analysis showing the protein expression of p62 and LC3 II/I in SD rat NP cells in response to TNF-α stimulation, glutamine, and AMPKα2 knockdown. **k** β-gal staining showing the level of senescence in SD rat NP cells in response to TNF-α stimulation, glutamine, and AMPKα1 knockdown. Scale bar = 100 μm. **l** β-gal staining showing the level of senescence in SD rat NP cells in response to TNF-α stimulation, glutamine, and AMPKα2 knockdown. Scale bar = 100 μm. **m** Yellow spots (GFP^+^mRFP^+^, autophagosomes) and red spots (GFP^-^mRFP^+^, autolysosomes) show autophagosomes and autolysosomes in SD rat NP cells in response to TNF-α stimulation, glutamine and AMPKα1 knockdown. Scale bar = 2 μm. **n** Statistical analysis showing the number of autophagosomes and autolysosomes in (**m**). ^*^p < 0.05.

To determine whether AMPKα functions synergistically with the effect of glutamine-induced cellular matrix synthesis promotion, autophagy induction, and senescence inhibition, the AMPK inhibitor compound C and AMPKα knockdown were used. The substantial reductions in the expression levels of COL2A1 and ACAN were reversed by glutamine and reversed again following compound C treatment, while the opposite change occurred in the expression of matrix-degrading enzymes (Fig. 7a, b). Compound C markedly heightened the protein expression of p53, p21, and p16 compared with glutamine (Fig. 7c, d). Furthermore, the senescent cell population in the glutamine group was profoundly increased after compound C treatment (Fig. 7e). Compound C significantly inhibited the production of autolysosomes, which was increased by glutamine (Fig. 7f, g). For AMPKα knockdown, the first sequence of siAMPKα1 and the third sequence of siAMPKα2 had the most profound effects (Fig. 7h). For the autophagy markers, AMPKα1 knockdown significantly enhanced the protein expression of p62 and decreased the LC3 II/I ratio compared to glutamine, while the consequence of AMPKα2 knockdown was minimal (Fig. 7I, j). AMPKα1 knockdown also notably boosted the population of SA-β-gal-positive cells in the glutamine group (Fig. 7k). However, the effect of AMPKα2 knockdown was not significant (Fig. 7l). Moreover, the diminished numbers of autophagosomes and autolysosomes with TNF-α treatment were reversed by glutamine and decreased by AMPKα1 knockdown (Fig. 7m, n). These results indicate that glutamine exerts its function by activating AMPKα.

### Glutamine promoted cellular matrix synthesis and autophagy but inhibited senescence in rats

**Fig. 8:**
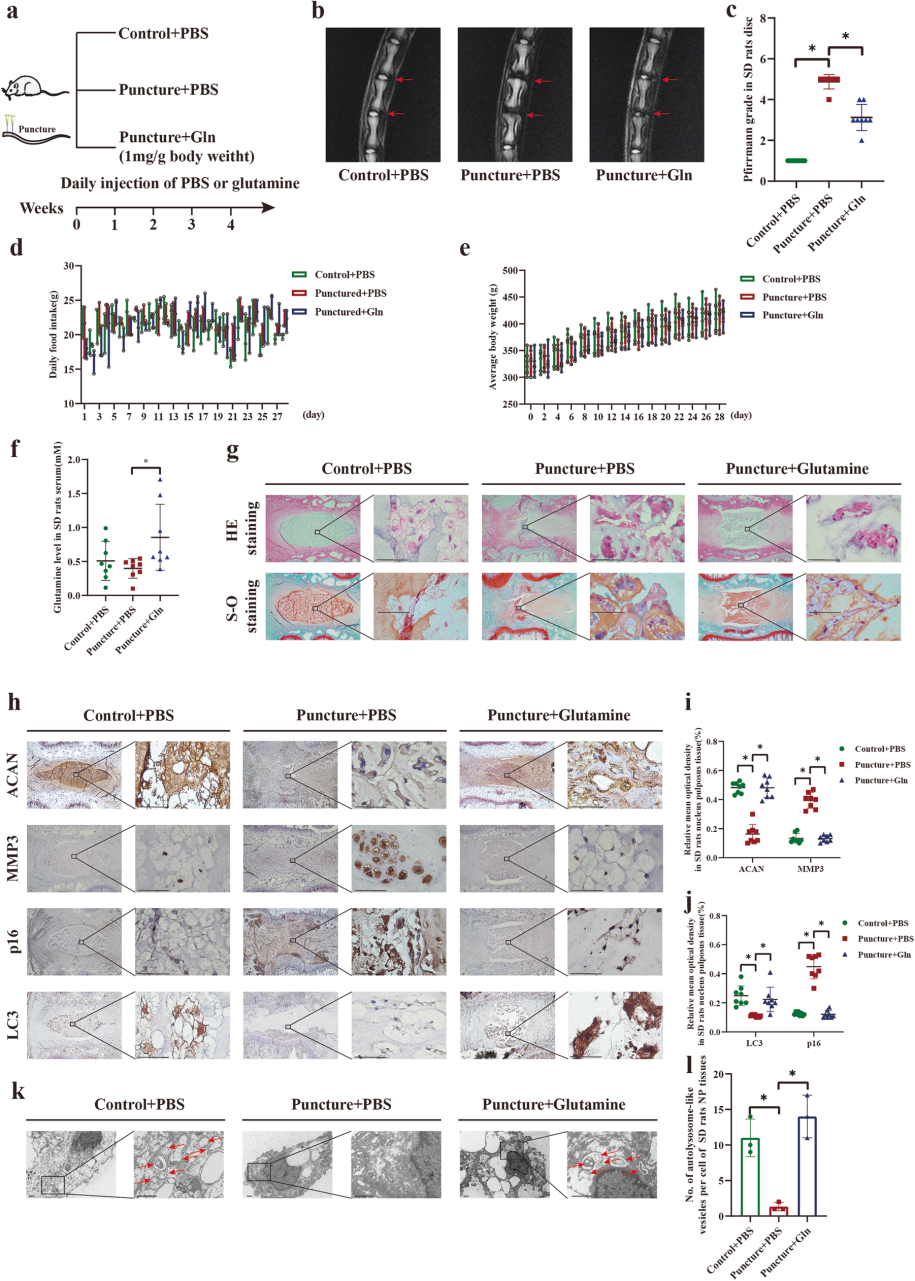
Glutamine promoted cellular matrix synthesis and autophagy but inhibited senescence in rats. **a** An illustration showing the drug administration process in the animal model. **b** T2-weighted MRI scans of SD rat NP tissues after daily injections of PBS or glutamine after puncture. **c** Pfirrmann grades of SD rat NP tissues in (**b**). **d** Daily food intake of SD rats after daily injections of PBS or glutamine (3, 3, and 2 SD rats in 3 cages and the average dietary intake of rats in each cage were measured). **e** Average body weights of SD rats after daily injections of PBS or glutamine. **f** Glutamine levels in the serum of SD rats after daily injections of PBS or glutamine. **g** HE and S-O staining of SD rat tissues after daily injections of PBS or glutamine. Scale bar = 500 μm. **h** IHC staining of ACAN, MMP3, p16, and LC3 expression in SD rats after daily injections of PBS or glutamine. Scale bar = 500 μm. **i**, **j** Semiquantitative analysis and statistical analysis of the IHC staining in (**h**). **k** TEM showing autolysosome-like vesicles in SD rats after daily injections of PBS or glutamine. Scale bar = 1 μm. **l** Statistical analysis showing autolysosome-like vesicles in the SD rats in (**k**). ^*^p < 0.05.

To assess the effect of glutamine in vivo, we established a caudal vertebra puncture model in rats and intraperitoneally injected PBS or glutamine daily for four weeks after surgery (Fig. 8a). We found a decrease in the T2-weighted signal in the IDD group, and these alterations were partially reversed by glutamine administration. The Pfirrmann grade was consistent with these qualitative observations (Fig. 8b, c). Daily food intake and average body weight showed no significant differences between the control, IDD, and glutamine groups (Fig. 8d, e). Serum glutamine concentrations in the glutamine group were considerably higher than those in the IDD group (Fig. 8f, *p* = 0.0336).

The collagen arrangement in the annulus fibrosus (AF) was significantly disordered, the boundary between the NP and AF was unclear, and the area of the NP was reduced in the IDD group. In contrast, glutamine administration partially alleviated these IDD changes (Fig. 8g). Compared with the control group, the level of ACAN was decreased and MMP3 was increased in the IDD group, and these effects were reversed by glutamine treatment (Fig. 8h, i). Likewise, glutamine reversed the high expression of p16 and the decreased LC3 expression in the IDD group (Fig. 8h, j) and increased the number of autolysosomes (Fig. 8k, l). These results indicate that glutamine can prevent NP cells from undergoing senescence and cellular degradation while promoting autophagy and cellular matrix synthesis.

## Discussion

The present study highlights the relationship between glutamine and IDD from the perspective of material metabolism disorders in IDD. The data revealed that severely degenerated human NP tissues and aging SD rat NP tissues had decreased glutamine levels but increased accumulation of lactate and lactylation. Glutamine suppressed glycolysis and reduced lactate production to downregulate AMPKα lactylation and upregulate AMPKα phosphorylation, resulting in decreased senescence and enhanced autophagy and matrix synthesis. These findings improve our understanding of the metabolic microenvironment in IDD and provide strategies and ideas for the prevention and treatment of IDD.

The effects of glutamine have been described in several cell types, such as adipose cells and cartilage cells^22,30^, and glutamine is widely used in several metabolic and inflammatory diseases, such as type 2 diabetes, postinfectious irritable bowel syndrome, and sepsis^40–43^. However, its effects on rats or human NP cells have not been studied. In the present study, we found significantly lower glutamine levels in severely degenerated NP tissues than in mildly degenerated NP tissues in both humans and SD rats. Furthermore, our results demonstrated that glutamine supplementation enhanced autophagy and cellular matrix synthesis and inhibited senescence in vitro and in vivo. This suggests that glutamines could also be a therapeutic medicine for IDD.

The intervertebral disc is an avascular structure consisting of the inner NP, upper and lower cartilage end-plates, and outer annulus fibrosus surrounding the NP. The diffusion rate of oxygen and nutrients from the synovial fluid is limited, and NP cells typically undergo glycolysis^44–46^. Glycolysis disorders are widely involved in the occurrence of many diseases, such as osteoarthritis, Alzheimer’s disease, and rheumatoid arthritis^47–49^. In the case of IDD, the end products of glycolysis, UDP-GlcNAc, and lactate, are closely related to senescence, autophagy, and pyroptosis in NP cells^15,50^. However, whether excessive glycolysis is involved in IDD and whether it is a target of glutamine are unclear. We found that the expression of key glycolytic enzymes was upregulated in degenerative NP cells and that glutamine downregulated the expression of glycolytic enzymes and the production of glycolytic intermediates. In addition, our results showed that inhibiting glycolysis promoted autophagy and cellular matrix synthesis and inhibited senescence.

Glutamine alters various metabolic processes, including the TCA cycle and urea cycle^20^. Several studies have shown that glutamine plays antitumor and anti-inflammatory roles through α-ketoglutarate and UDP-GlcNAc^22,29^. Metabonomic analysis was performed to analyze polar metabolites that may influence the phenotype after exposure to TNF-α and glutamine. The earliest metabonomic studies on NP cells showed that the absence of MCT4 induced lactate accumulation and changes in pyruvate metabolism and TCA cycle metabolites^22^. In the present study, we found alterations in the glycolytic pathway and glycolytic products in human NP cells stimulated by TNF-α and glutamine, and glutamine significantly reversed the increase in lactate accumulation induced by TNF-α.

In human adipocytes, a high-level of glutamine inhibits glycolysis and decreases the level of the glycolytic product UDP-GlcNAc, inhibiting adipose inflammation^22^. Meanwhile, glutamine can inhibit glycolysis in osteocytes under microgravity, thereby rescuing subsequent Ca^2+^ oscillations, restoring the mechanical sensitivity of osteocytes, and promoting the recovery of disuse osteoporosis^26^. Here, our data show that glutamine attenuates glycolysis and lactate accumulation to benefit the local metabolic environment in NP cells.

Recently, posttranslational modifications (PTMs) have been shown to be involved in IDD^50,51^. Lactylation, which is one of the most decisive PTMs, is widely involved in multitudinous diseases, such as hepatocellular carcinoma and Alzheimer’s disease^32,34^, and is mediated by the metabolic substrate lactate and the transferase p300. Decreases in lactate and p300 could alleviate lactylation, relieving disease progression^52,53^. Our study showed that lactylation was involved in the progression of IDD and that reducing lactylation could promote cellular matrix synthesis and autophagy and inhibit senescence. However, p300 expression was not altered by TNF-α, glutamine, or lactate in NP cells. Furthermore, p300 levels were not different between mildly degenerated human NP tissues and severely degenerated human NP tissues. These results show that lactate but not p300 is responsible for the process of lactylation in NP cells and tissues and the effect of glutamine on NP. Meanwhile, glutamine is equivalent to glycolysis inhibitors and lactylation inhibitors in hampering lactylation in NP cells.

Glutamine, mTOR and autophagy were connected in many diseases, and glutaminolysis enhancement could activate mTOR^54–56^. To explore the effect of glutamine to mTOR, we supplied western blot experiment and found that glutamine have no influence to mTOR (Supplementary Fig. 1d). To investigate the influence of lactylation and identify the pathway involved in the regulation of glutamine on NP cells, we performed transcriptomic KEGG enrichment analysis and found significant changes in multiple pathways. Among these pathways, the AMPK pathway is recognized as the upstream regulator of cellular matrix metabolism, autophagy and senescence of NP cells^18,35,38^, and anabolism escalation, autophagy promotion, and senescence inhibition have been demonstrated to be effective in the treatment of IDD^50,57–59^. AMPK is a conserved serine/threonine protein kinase called the cellular “energy receptor” and is a key molecule in the pathway associated with material and energy metabolism^60,61^. AMPK has become an essential target for the treatment of metabolic diseases, such as type 2 diabetes and obesity, and it is also involved in IDD^39,62,63^. AMPKα is the core component of AMPK, and its phosphorylation is the key to AMPK activation^64^. Similar to a previous report that glutamine could activate AMPKα in piglet intestinal inflammation^65^, our findings demonstrated that glutamine-induced the phosphorylation of AMPKα in vitro and in vivo, and the phosphorylation of AMPKα was inhibited in 22-month-old SD rat NP tissues and severely degenerated human NP tissues. While phosphorylation is the activated form of AMPKα^66^, the lactylation of AMPKα has not been previously reported. Our data showed that the lactylation of AMPKα occurred in NP cells and that glutamine could retard the lactylation of AMPKα. Furthermore, inhibiting AMPKα lactylation could induce the phosphorylation of AMPKα, activating the AMPK pathway. These results indicated the presence of competitive inhibition between the lactylation and phosphorylation of AMPKα. AMPKα has extensive metabolic relationships, such as O-GlcNAcylation and oxidative modifications, which could affect its activation^67,68^. This relationship between the lactylation and phosphorylation of AMPKα has not been previously reported. Our findings reveal that glutamine contributed to the lactylation of AMPKα inhibition, which escalated the phosphorylation of AMPKα.

Autophagy is a lysosomal-dependent metabolic process, which is necessary for a range of biological activities, not only as an adaptive response to pathological stress, but also to maintain cellular homeostasis under physiological conditions^69^. The role of autophagy in IDD is controversial, with difference from protection to destruction, and targeting autophagy remains an important direction for treating IDD^50,70–72^. Maybe, the balance between treating IDD is to neither suppress autophagy nor overactivate autophagy. In our early experiments and other articles, autophagy could support anabolic process and inhibit catabolic process^18,58,71,73,74^.

However, there still remains limitations in our study. First, the sites of lactylation of AMPKα were not clear now. Second, the experimental results will be more convincing if we supplement animal experiments with 2-DG, C646, and lactate treatments.

**Fig. 9:**
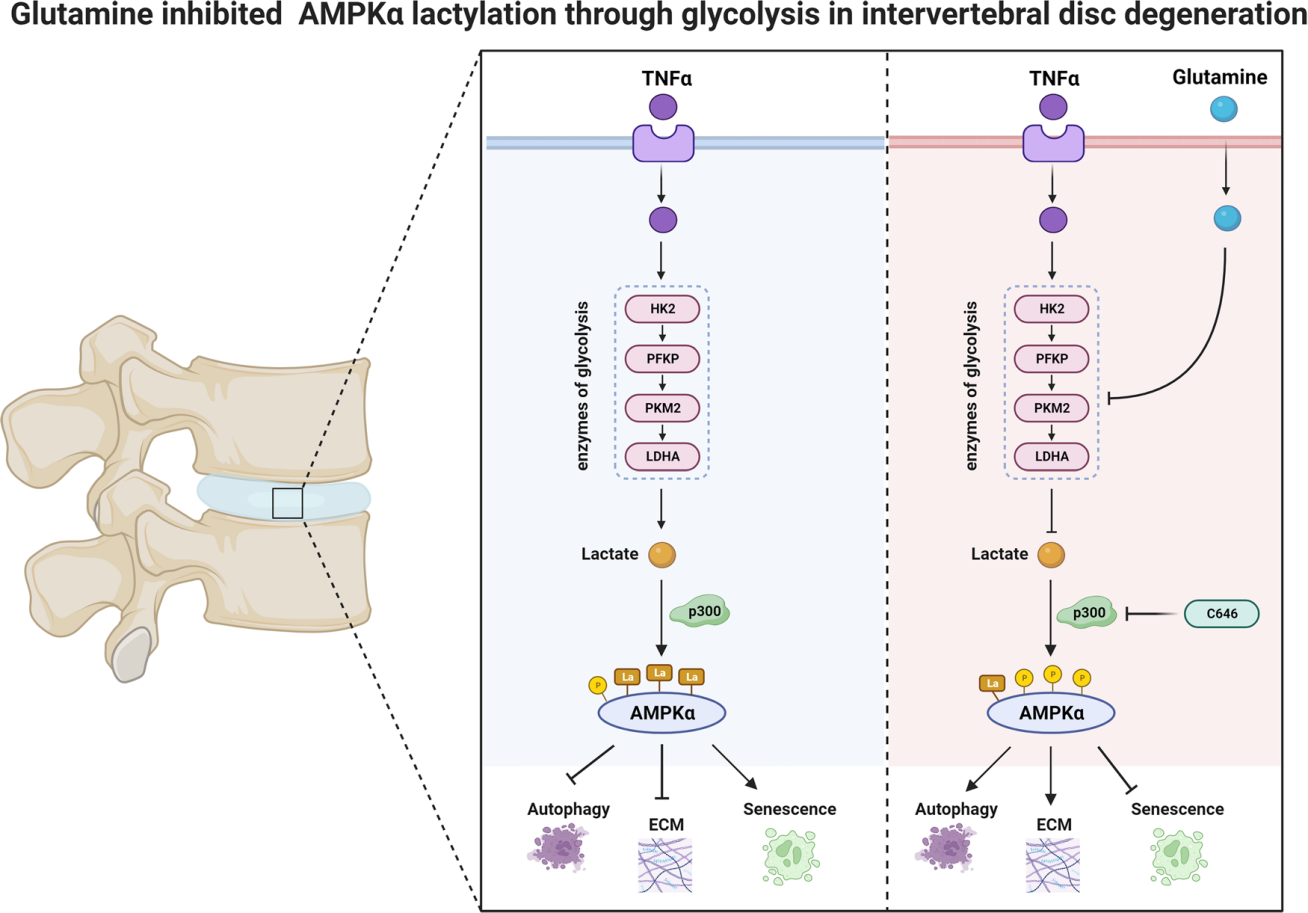
An illustration summarizing the mechanisms linking glutamine-induced autophagy and cellular matrix synthesis and inhibition of senescence in NP cells. Increased levels of TNF-α have been observed in degenerative NP tissues, which intensifies the glycolysis process within NP cells. However, low concentrations of glutamine, a naturally occurring metabolite in vivo, are insufficient to inhibit this upsurge in the glycolysis pathway. The heightened glycolysis prompts a steep rise in lactate levels, triggering the lactylation of AMPKα, thereby inhibiting its phosphorylation. This sequence of events deactivates the AMPK pathway, leading to decreased matrix synthesis in NP cells, escalated degradation, reduced autophagy, and exacerbated senescence. Conversely, the supplementation of glutamine augments intracellular glutamine concentration, which inhibits glycolysis and activates the AMPK pathway. As a result, there is a surge in matrix synthesis, mitigated degradation, enhanced autophagy, and the deceleration of senescence in NP cells. This counteracts and prevents the progression of IDD. Throughout this process, the glycolysis inhibitor 2-DG and the lactylation inhibitor C646 prevent IDD due to their respective repressive effects.

In total, a strong link between glutamine and IDD was established in the current study by analyzing clinical samples, animal models, and cellular mechanisms and systematically elucidated the mechanism by which glutamine inhibits glycolysis and regulates the lactylation of AMPKα in matrix metabolism, senescence and autophagy in NP cells (Fig. 9). The identification of the linkages between glutamine, glycolysis, and lactylation in IDD provides insights into the pathogenesis of IDD and offers potential strategies for IDD prevention and treatment based on regulating metabolic disorders.

## Materials and methods

### Patient samples

This study involved the collection of 24 NP tissue samples from 24 patients (10 males and 14 females, aged 34 to 71 years) at Sun Yat-sen Memorial Hospital. The Pfirrmann grade of discs was used to evaluate the degree of NP tissue degeneration. Samples with a Pfirrmann grade ≤ III were the mildly degenerated group, while samples with a Pfirrmann grade ≥ IV were the severely degenerated group. Part of the NP tissue was embedded in paraffin to prepare tissue sections, and the other part was used for glutamine and lactate analysis. The study protocol was approved by the Ethics Committee of Sun Yat-sen Memorial Hospital [(No. (2022) 056)]. All ethical regulations relevant to human research participants were followed.

### Cell culture

After routine disinfection with 70% alcohol and complex iodine, the tail of SD rat (male, 3 months old) was removed at the root, the skin and muscle tissue were quickly separated, the fibrous ring was cut open, and the NP tissue was removed and immediately placed in Dulbecco’s modified Eagle’s medium (DMEM) (Gibco, USA). After digestion, the NP cells were cultured in DMEM with 10% fetal bovine serum (FBS) (Gibco, USA)^13^. When NP cells were treated with relevant stimulations, DMEM with 1% FBS was used. Normal-level glutamine(4 mM) and high-level glutamine (16 mM) were concocted on the base of glutamine-deficient DMEM (Glutamine(Sigma, G3126), glutamine-deficient DMEM(Sigma, 11960044)). L-lactate (6 mM) was added to the cells for further study. The study protocol was approved by the Institutional Research Ethical Committee of Sun Yat-sen University. We have complied with all relevant ethical regulations for animal use.

Some human NP cells were purchased from ScienCell (ScienCell, USA), and others were isolated from Pfirrmann grade II human NP tissues. After cut into pieces and washed, the pieces of NP tissues were digested using type II collagenase at 37 °C. After centrifuged, the NP cells were incubated in DMEM with 10% FBS at 37 °C under 5% CO_2_^75^. The study protocol was approved by the Ethics Committee of Sun Yat-sen Memorial Hospital [(No. (2022) 056)]. All ethical regulations relevant to human research participants were followed.

### Western blot analysis

Sodium dodecyl sulfate‒polyacrylamide gel electrophoresis (SDS‒PAGE) gels (ACE, China) and poly(vinylidene fluoride) (PVDF) membranes (Merck, USA) were used to separate and electrotransfer the protein samples. The blots were incubated with the following primary antibodies (Supplementary Table 1). After an overnight incubation, the membranes were washed and incubated with secondary antibodies (1:5000, ABclonal, China) for further testing.

### Nontargeted metabolomics and transcriptomics analysis of human NP cells

Human primary NP cells were divided into three groups: NC group, TNF-α group, and TNF-α plus glutamine group. Metabolite identification and transcriptome analysis were conducted by Shanghai Applied Protein Technology Co., Ltd. (Shanghai, China). Variable importance for the project (VIP) > 1 and *p* < 0.05 were the standards to determine significantly different metabolites. “The negative ionization mode” and “the positive ionization mode” represent two modes of data collection in metabolomics. The transcriptome data have been uploaded to the GEO database (GEO: GSE226186).

### Measurement of glutamine and lactate levels

Glutamine levels were measured using the EnzyChrom Glutamine Kit (BioAssay Systems, USA). Glutamine was extracted from 40–60 mg of NP tissue from humans and SD rats. Rat serum was separated from whole blood, and the glutamine levels were tested according to the manufacturer’s protocol. Lactate was extracted from 50–250 mg of NP tissue and was measured using a specific test kit (Solarbio, China) and stimulated NP cells with L-Lactate (Sigma–Aldrich, USA).

### β-gal staining

After treatment, the cells were fixed using β-gal fix solution (Beyotime, China) for 15 min and washed with PBS. Subsequently, the fixed cells were incubated with β-gal staining working solution (Beyotime, China) overnight at 37 °C and then observed and imaged.

### siRNA transfection

For RNA interference of p300, AMPKα1 and AMPKα2, rat NP cells were transfected with siRNA using JetPRIME transfection reagent (Polyplus, France). The siRNA sequences were designed by IGEBIO (IGEBIO, China) (Supplementary Table 2).

### Transmission electron microscopy (TEM)

After treatment, rat NP cells were collected and fixed overnight using 2.5% glutaraldehyde at 4 °C. The fixed cells were washed with PBS, treated at 4 °C with 1% ozone tetroxide for 2 h, washed again in the buffer, and dehydrated with an increasing series of ethanol. Then, the samples were thoroughly embedded in Epon 812. A Leica UC7 ultrafine slicer was used to cut the samples into ultrafine slices approximately 100 nm thick, which were then steeped in dioxyuranium acetate for 20 min and lead citrate for 12 min and observed using a transmission electron microscope (FEI, USA).

### mRFP-GFP-LC3

The mRFP-GFP-LC3 adenoviral vector (HanBio, China) was introduced into NP cells according to the manufacturer’s instructions. After 24 h of transfection, NP cells with fluorescent spots were observed under a laser confocal scanning microscope (Zeiss, Germany) and semiautomatic fluorescence microscope (Olympus, Japan). Yellow spots (GFP^+^ and mRFP^+^) indicated autophagosomes; red spots (GFP^−^ and mRFP^+^) corresponded to autolysosomes.

### Coimmunoprecipitation (CoIP)

The whole cell extract was collected with cell lysis buffer (Beyotime, China). The mixed liquid was centrifuged, and the supernatant was incubated with normal rabbit IgG (2729 S, CST, RRID: AB_1031062) or anti-AMPKα (1:50, 2532 S, CST, RRID: AB_330331). After overnight incubation at 4 °C, the immunocomplex and magnetic beads (10003D, Invitrogen, USA) were incubated at room temperature for 2 h. Then, the magnetic beads were collected and washed 3 times with IP washing buffer and mixed with loading buffer, and we separated the magnetic beads and proteins for further analysis.

### Immunohistochemical staining (IHC)

The sections were dewaxed with diethanol, rehydrated with decreasing concentrations of ethanol, and repaired with ethylene diamine tetraacetic acid (EDTA) recovery buffer. Endogenous peroxidase activity was quenched with 3% hydrogen peroxide. Subsequently, the slides were sealed with goat serum for 30 min and incubated overnight at 4 °C with PBS or with the following primary antibodies (Supplementary Table 3). The slides were individually covered with secondary antibodies and HRP-conjugated streptavidin for 30 min and then incubated with DAB solution (Zhongshan Jinqiao Biotechnology Co., Ltd., China).

### Animal model

The animal experiments were approved by the Institutional Research Ethical Committee of Sun Yat-sen University (SYSU-IACUC-2022-000365 and SYSU-IACUC-2022-001999). We have complied with all relevant ethical regulations for animal use.

Twenty-four male Sprague-Dawley (SD) rats (3 months old, 320–350 g) were randomly divided into three groups (eight rats in each group): control with intraperitoneal injection of phosphate buffer solution (PBS) (control group), puncture with intraperitoneal injection of PBS (IDD group), and puncture with intraperitoneal injection of glutamine (glutamine group).

General anesthesia was administered by an intraperitoneal injection of 3% pentobarbital sodium (0.1 ml/100 g). After disinfection with 75% ethanol, a 20-gauge needle was used to vertically penetrate the midpoint of the intervertebral discs of Co7/8 and Co8/9. The needle was inserted 5 mm, rotated steadily clockwise and pulled out after being held for 30 s. Glutamine (1 g/kg) was injected intraperitoneally every day in the glutamine group, and the same volume of PBS was injected in the other groups. The daily body weight and daily food intake of the rats were recorded. Four weeks after the operation, the caudal disc was removed for immunohistochemical staining (discs at Co7/8) and TEM (discs at Co8/9) (Fig. 8a).

Ten male SD rats (3 months: 5, 22 months: 5) were obtained, and discs at Co7/8 were used for histological staining, while the other discs were used for glutamine and lactate analysis.

### Statistics and reproducibility

The data were collected from at least three independent experiments and expressed as the mean ± standard deviation (SD). Unpaired double-tailed Student’s *t*-test was used to compare differences between two groups, while one-way ANOVA was used to evaluate differences between multiple groups. A value of *p* < 0.05 was considered statistically significant.

### Reporting summary

Further information on research design is available in the Nature Portfolio Reporting Summary linked to this article.

### Supplementary information

Supplementary Information

Description of Additional Supplementary Files

Supplementary Data

Reporting Summary

## Data Availability

The sequencing data (RNA) has been uploaded in GEO (GEO: GSE226186). The uncropped western blotting images were exhibited in Supplementary Fig. 9. The source data of the graphs in figures were exhibited in Supplementary Data.
