# Supplementary material for: Glutamine suppresses senescence and promotes autophagy through glycolysis inhibition-mediated AMPKα lactylation in intervertebral disc degeneration
Source: Commun Biol. 2024 Mar 14;7:325. doi: 10.1038/s42003-024-06000-3 (PMC10940657; doi:10.1038/s42003-024-06000-3)
Supplement: Supplementary file 1 — Supplementary Information [file 42003_2024_6000_MOESM1_ESM.pdf]

Supplementary Figure 1. **AICAR induced autophagy, inhibited senescence and promoted anabolism process and inhibited catabolism process.**

(a) Western blot analysis showing the protein expression of COL2A1, ACAN, ADAMTS5, and MMP3 in SD rat NP cells stimulated with TNF-α and AICAR. (b) Semiquantitative analysis and statistical analysis of the Western blots in (a). (c) Western blot analysis showing the protein expression of p21 in SD rat NP cells stimulated with TNF-α and AICAR. (d) Semiquantitative analysis and statistical analysis of the Western blots in (c). (e) Western blot analysis showing the protein expression of p62 in SD rat NP cells stimulated with TNF-α and AICAR. (f) Semiquantitative analysis and statistical analysis of the Western blots in (e). (g) Western blot analysis showing the protein expression of LC3 II/I in SD rat NP cells stimulated with TNF-α and AICAR. (h) Semiquantitative analysis and statistical analysis of the Western blots in (g). (i) β-gal staining showing the level of senescence in SD rat NP cells in response to stimulation with TNF-α and AICAR. Scale bar =100 μm. (j) Western blot analysis showing the protein expression of p-mTOR and mTOR in SD rat NP cells stimulated with TNF-α and glutamine. (k) Semiquantitative analysis and statistical analysis of the Western blots in (j).

Supplementary Figure 2. KEGG Enrichment Analysis Showing the Different Pathways in Human NP cells in Response to TNF-α and Glutamine stimulation (TNF-α and TNF-α+glutamine).

Supplementary Figure 3. Intracellular glutaminolysis of NP cells under the stimulation of TNF-α and glutamine of metabolomics analysis.

Supplementary Figure 4. Changes in key glycolysis enzymes in human NP cells in response to TNF-α and glutamine stimulation of transcriptomics analysis.

Supplementary Figure 5. Changes in the intermediate products of glycolysis in human NP cells stimulated with TNF-α and glutamine of metabolomics analysis.

Supplementary Figure 6. The identified metabolites in positive ionization mode of metabolomics

analysis.

(a)The proportion of identified metabolites in each chemical classification. (b)The positive ionization mode of hierarchical clustering analysis showing other changes in metabolites. (c) The positive ionization mode of the volcano plot showing other changes in metabolites in response to TNF-α stimulation and control conditions. (d) The positive ionization mode of the volcano plot showing other changes in metabolites in response to stimulation with glutamine and TNF-α.

Supplementary Figure 7. A significant change in metabolites of negative ionization mode of hierarchical clustering analysis.

Supplementary Figure 8. A significant change in metabolites of positive ionization mode of hierarchical clustering analysis.

Supplementary Figure 9. The full, unedited blots in Figures.

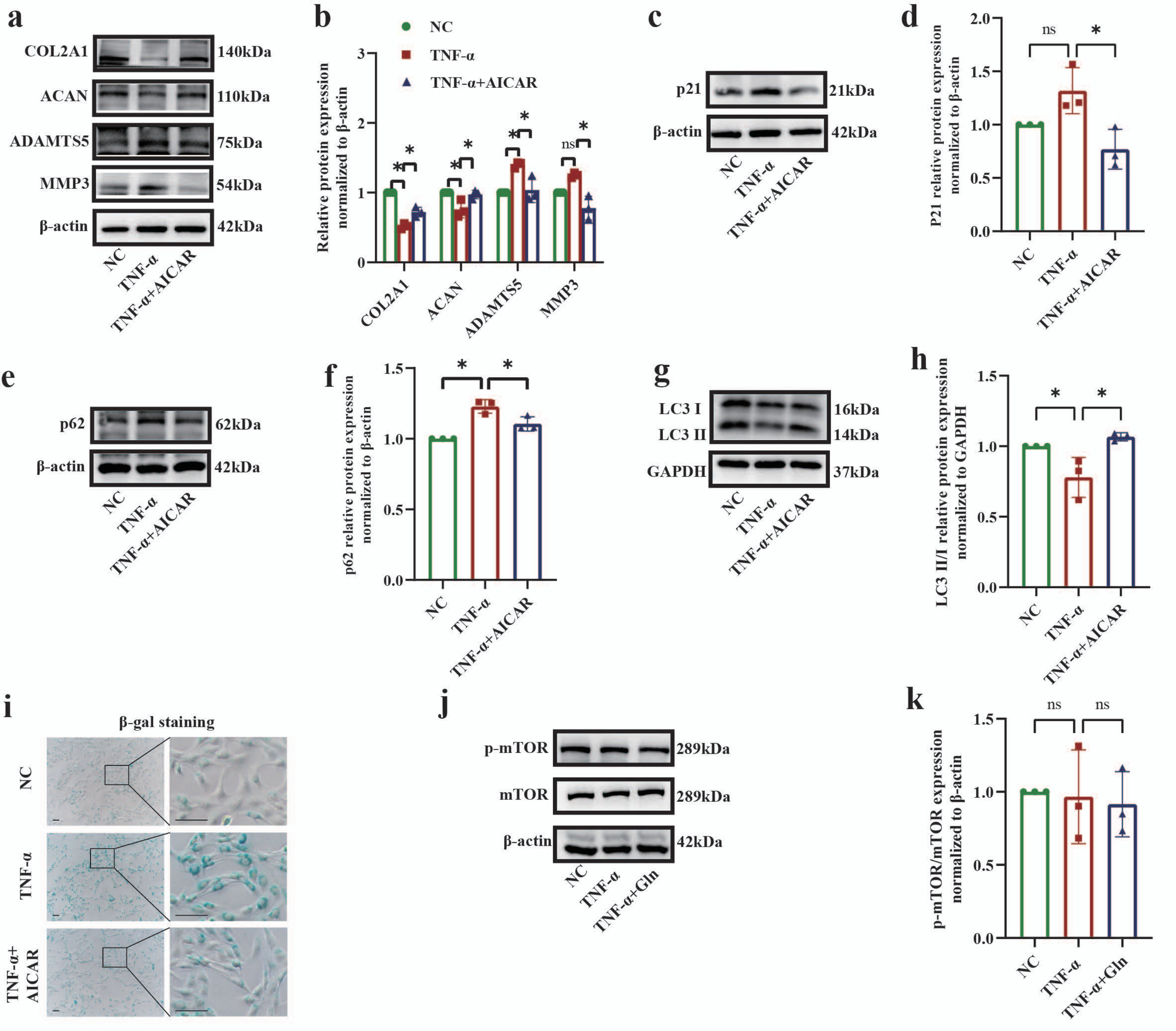
a
COL2A1
140kDa
ACAN
110kDa
ADAMTS5
75kDa
MMP3
54kDa
β-actin
42kDa
NC
TNF-α
TNF-α+AICAR
b
NC
TNF-α
TNF-α+AICAR
Relative protein expression normalized to β-actin
COL2A1
ACAN
ADAMTS5
MMP3
ns
c
p21
21kDa
β-actin
42kDa
d
P21 relative protein expression normalized to β-actin
ns
e
p62
62kDa
β-actin
42kDa
f
p62 relative protein expression normalized to β-actin
g
LC3 I
16kDa
LC3 II
14kDa
GAPDH
37kDa
h
LC3 II/I relative protein expression normalized to GAPDH
i
β-gal staining
NC
TNF-α
TNF-α+ AICAR
j
p-mTOR
289kDa
mTOR
289kDa
β-actin
42kDa
NC
TNF-α
TNF-α+Gln
k
p-mTOR/mTOR expression normalized to β-actin
ns
ns
TNF-α+Gln

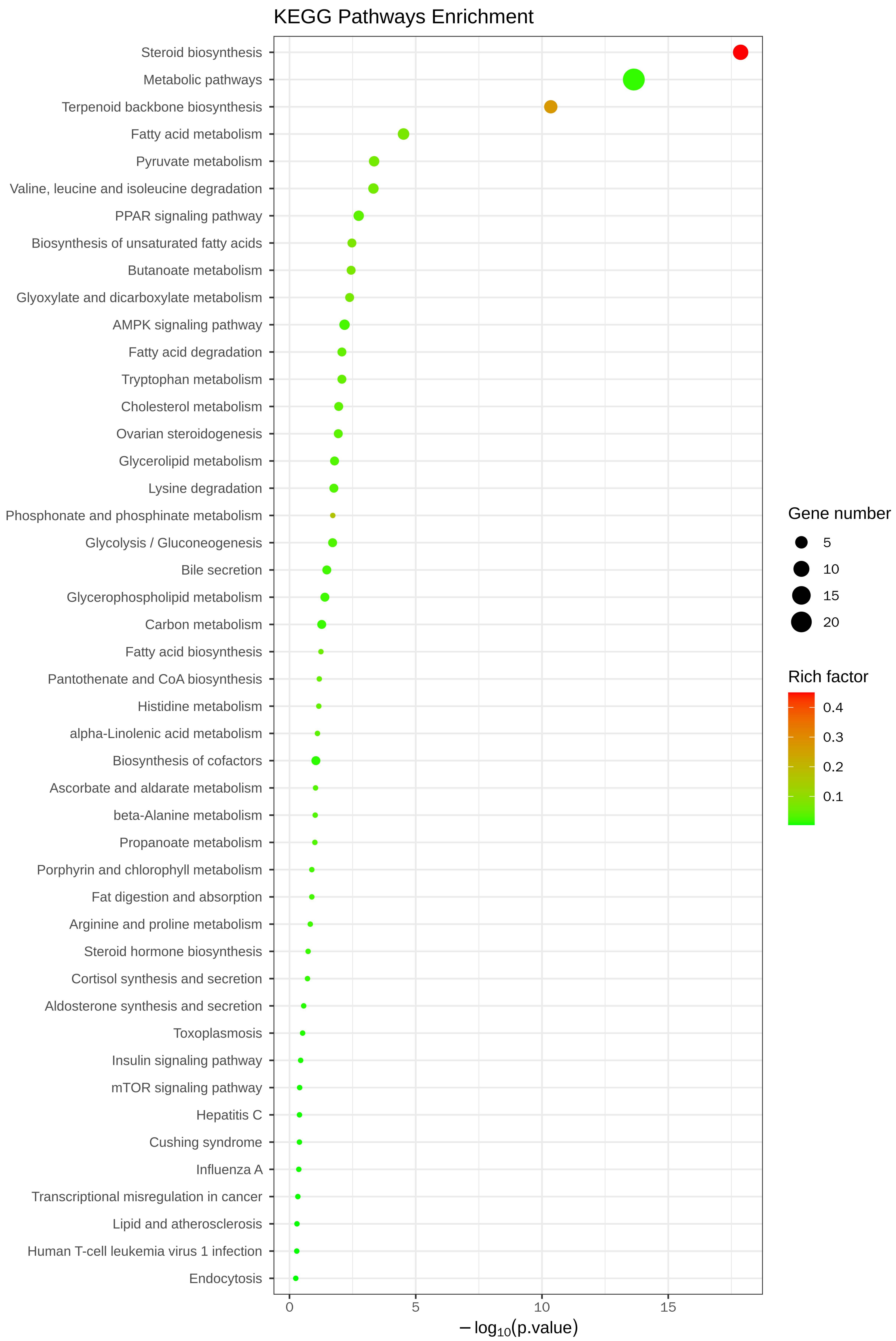

KEGG Pathways Enrichment
Steroid biosynthesis
Metabolic pathways
Terpenoid backbone biosynthesis
Fatty acid metabolism
Pyruvate metabolism
Valine, leucine and isoleucine degradation
PPAR signaling pathway
Biosynthesis of unsaturated fatty acids
Butanoate metabolism
Glyoxylate and dicarboxylate metabolism
AMPK signaling pathway
Fatty acid degradation
Tryptophan metabolism
Cholesterol metabolism
Ovarian steroidogenesis
Glycerolipid metabolism
Lysine degradation
Phosphonate and phosphinate metabolism
Glycolysis / Gluconeogenesis
Bile secretion
Glycerophospholipid metabolism
Carbon metabolism
Fatty acid biosynthesis
Pantothenate and CoA biosynthesis
Histidine metabolism
alpha-Linolenic acid metabolism
Biosynthesis of cofactors
Ascorbate and aldarate metabolism
beta-Alanine metabolism
Propanoate metabolism
Porphyrin and chlorophyll metabolism
Fat digestion and absorption
Arginine and proline metabolism
Steroid hormone biosynthesis
Cortisol synthesis and secretion
Aldosterone synthesis and secretion
Toxoplasmosis
Insulin signaling pathway
mTOR signaling pathway
Hepatitis C
Cushing syndrome
Influenza A
Transcriptional misregulation in cancer
Lipid and atherosclerosis
Human T-cell leukemia virus 1 infection
Endocytosis
0
5
10
15
$-\log_{10}(\text{p.value})$
Gene number
5
10
15
20
Rich factor
0.4
0.3
0.2
0.1

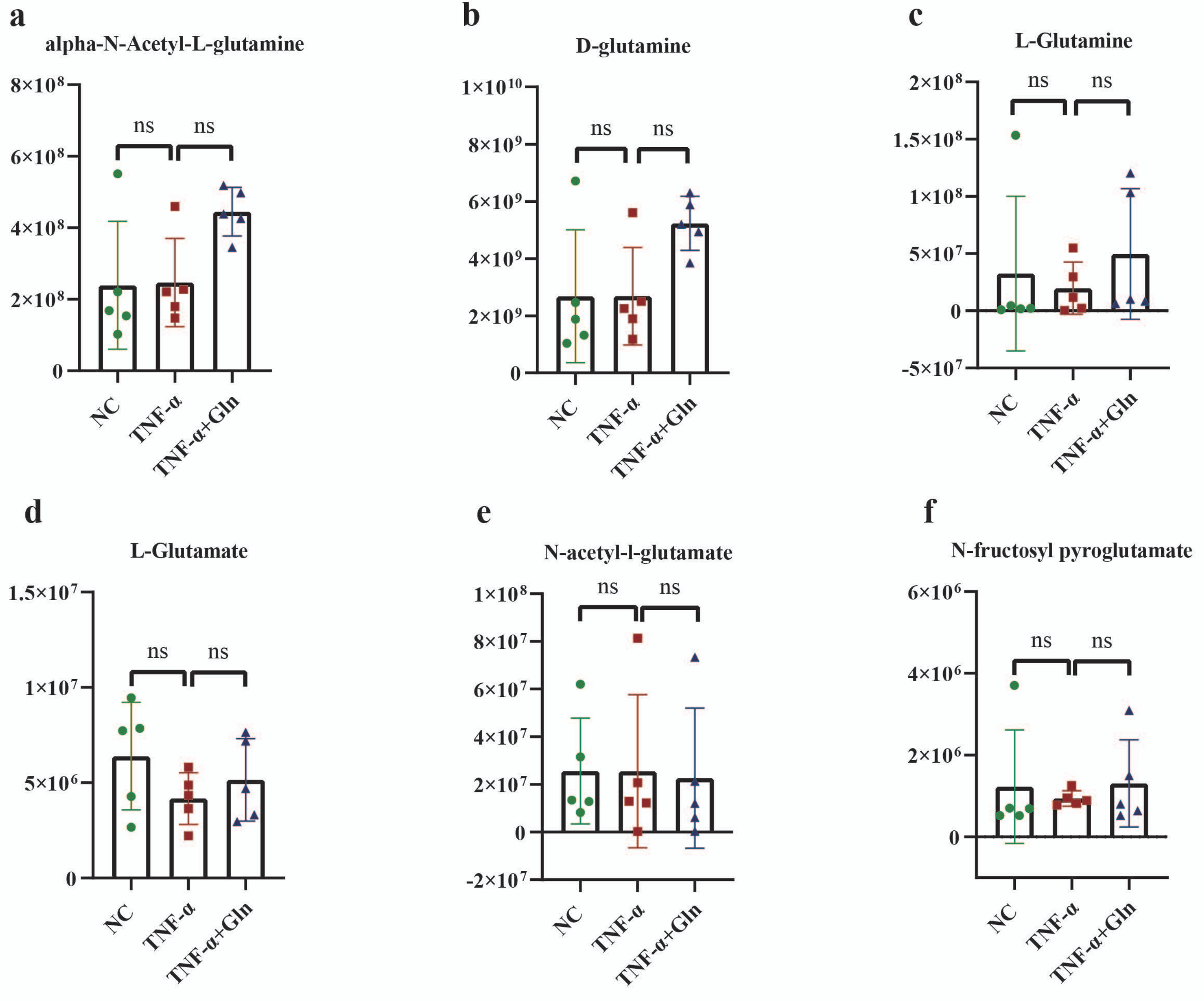
a
alpha-N-Acetyl-L-glutamine
8×10^8
6×10^8
4×10^8
2×10^8
0
ns
ns
NC
TNF-α
TNF-α+Gln
b
D-glutamine
1×10^10
8×10^9
6×10^9
4×10^9
2×10^9
0
ns
ns
NC
TNF-α
TNF-α+Gln
c
L-Glutamine
2×10^8
1.5×10^8
1×10^8
5×10^7
0
-5×10^7
ns
ns
NC
TNF-α
TNF-α+Gln
d
L-Glutamate
1.5×10^7
1×10^7
5×10^6
0
ns
ns
NC
TNF-α
TNF-α+Gln
e
N-acetyl-l-glutamate
1×10^8
8×10^7
6×10^7
4×10^7
2×10^7
0
-2×10^7
ns
ns
NC
TNF-α
TNF-α+Gln
f
N-fructosyl pyroglutamate
6×10^6
4×10^6
2×10^6
0
ns
ns
NC
TNF-α
TNF-α+Gln

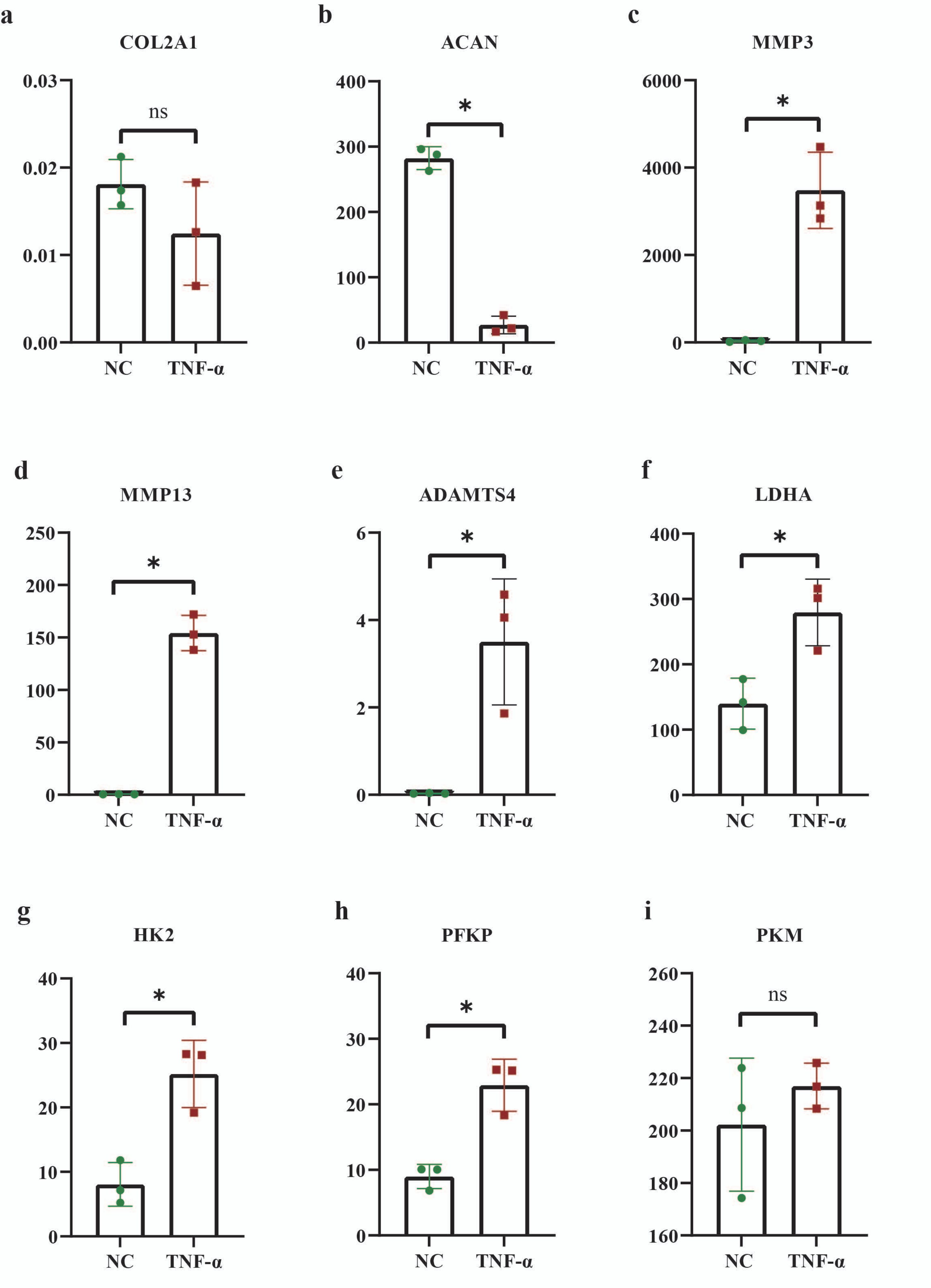

a
COL2A1
ns
0.03
0.02
0.01
0.00
NC
TNF-α
b
ACAN
*
400
300
200
100
0
NC
TNF-α
c
MMP3
*
6000
4000
2000
0
NC
TNF-α
d
MMP13
*
250
200
150
100
50
0
NC
TNF-α
e
ADAMTS4
*
6
4
2
0
NC
TNF-α
f
LDHA
*
400
300
200
100
0
NC
TNF-α
g
HK2
*
40
30
20
10
0
NC
TNF-α
h
PFKP
*
40
30
20
10
0
NC
TNF-α
i
PKM
ns
260
240
220
200
180
160
NC
TNF-α

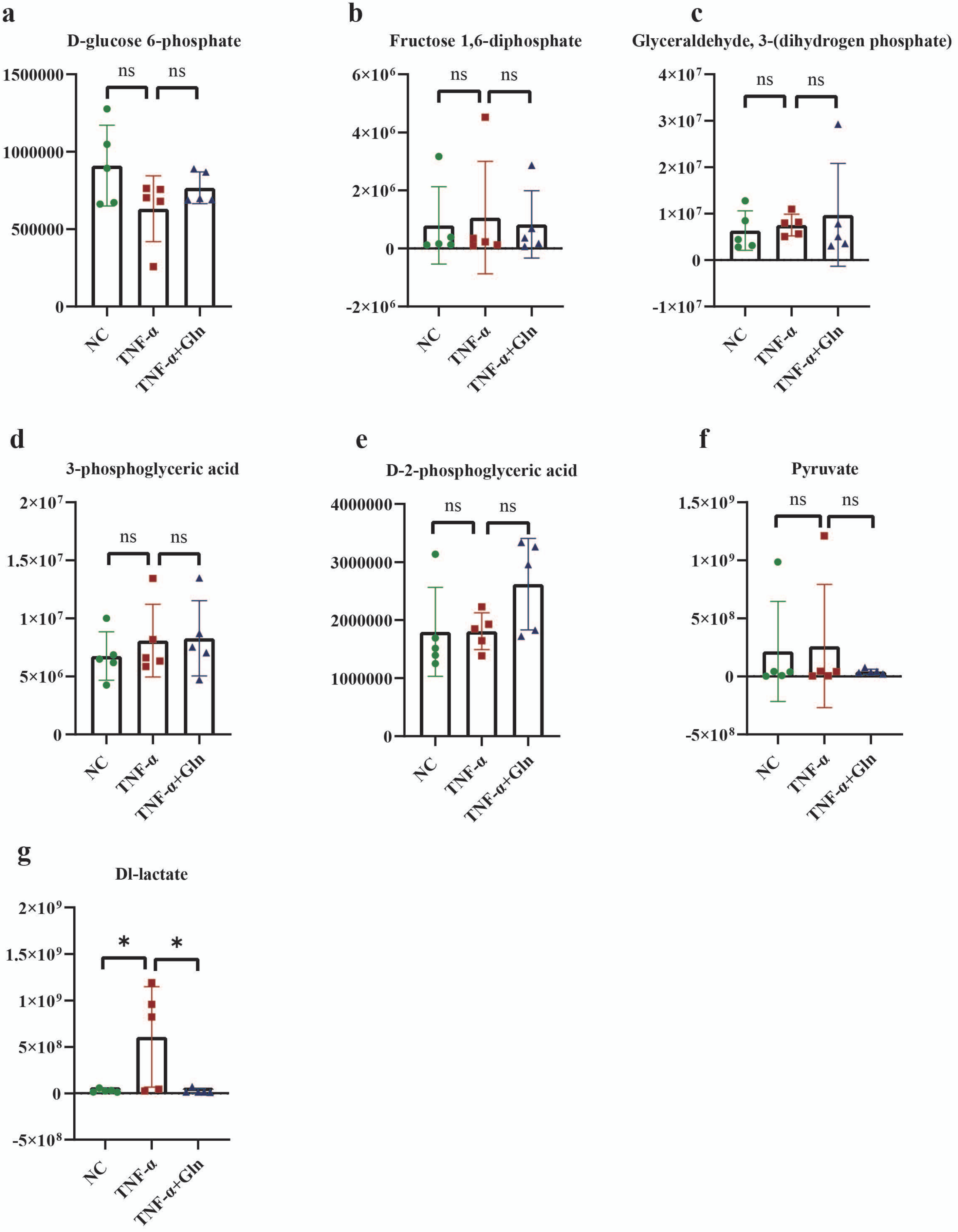

a
D-glucose 6-phosphate
1500000
1000000
500000
0
ns
ns
NC
TNF-α
TNF-α+Gln
b
Fructose 1,6-diphosphate
6×10^6
4×10^6
2×10^6
0
-2×10^6
ns
ns
NC
TNF-α
TNF-α+Gln
c
Glyceraldehyde, 3-(dihydrogen phosphate)
4×10^7
3×10^7
2×10^7
1×10^7
0
-1×10^7
ns
ns
NC
TNF-α
TNF-α+Gln
d
3-phosphoglyceric acid
2×10^7
1.5×10^7
1×10^7
5×10^6
0
ns
ns
NC
TNF-α
TNF-α+Gln
e
D-2-phosphoglyceric acid
4000000
3000000
2000000
1000000
0
ns
ns
NC
TNF-α
TNF-α+Gln
f
Pyruvate
1.5×10^9
1×10^9
5×10^8
0
-5×10^8
ns
ns
NC
TNF-α
TNF-α+Gln
g
Dl-lactate
2×10^9
1.5×10^9
1×10^9
5×10^8
0
-5×10^8
*
*
NC
TNF-α
TNF-α+Gln

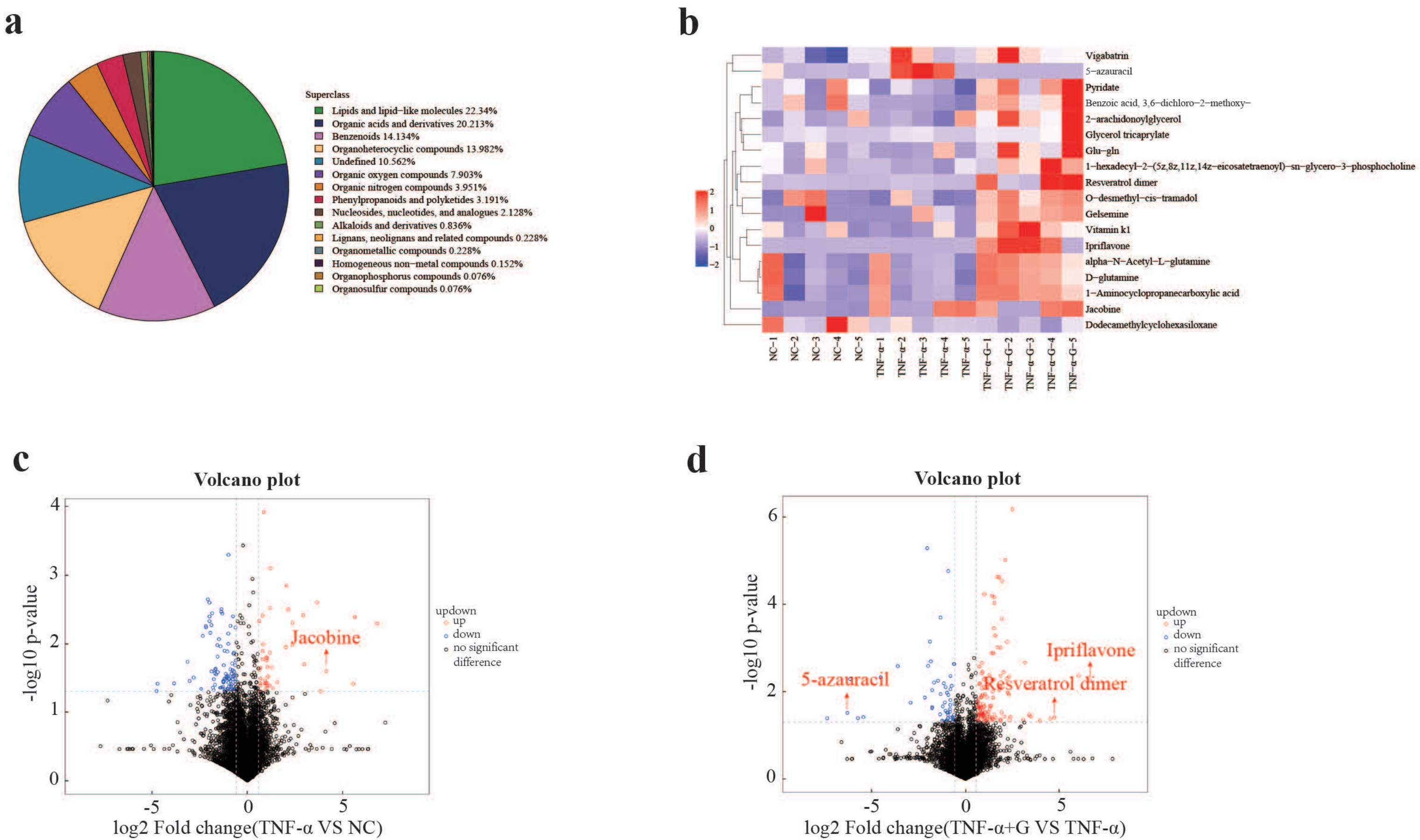

a
Superclass
Lipids and lipid−like molecules 22.34%
Organic acids and derivatives 20.213%
Benzenoids 14.134%
Organoheterocyclic compounds 13.982%
Undefined 10.562%
Organic oxygen compounds 7.903%
Organic nitrogen compounds 3.951%
Phenylpropanoids and polyketides 3.191%
Nucleosides, nucleotides, and analogues 2.128%
Alkaloids and derivatives 0.836%
Lignans, neolignans and related compounds 0.228%
Organometallic compounds 0.228%
Homogeneous non−metal compounds 0.152%
Organophosphorus compounds 0.076%
Organosulfur compounds 0.076%
b
Vigabatrin
5−azauracil
Pyridate
Benzoic acid, 3,6−dichloro−2−methoxy−
2−arachidonoylglycerol
Glycerol tricaprylate
Glu−gln
1−hexadecyl−2−(5z,8z,11z,14z−eicosatetraenoyl)−sn−glycero−3−phosphocholine
Resveratrol dimer
O−desmethyl−cis−tramadol
Gelsemine
Vitamin k1
Ipriflavone
alpha−N−Acetyl−L−glutamine
D−glutamine
1−Aminocyclopropanecarboxylic acid
Jacobine
Dodecamethylcyclohexasiloxane
NC−1 NC−2 NC−3 NC−4 NC−5 TNF−α−1 TNF−α−2 TNF−α−3 TNF−α−4 TNF−α−5 TNF−α−G−1 TNF−α−G−2 TNF−α−G−3 TNF−α−G−4 TNF−α−G−5
c
Volcano plot
-log10 p-value
log2 Fold change(TNF-α VS NC)
Jacobine
updown
up
down
no significant difference
d
Volcano plot
-log10 p-value
log2 Fold change(TNF-α+G VS TNF-α)
5-azauracil
Ipriflavone
Resveratrol dimer
updown
up
down
no significant difference

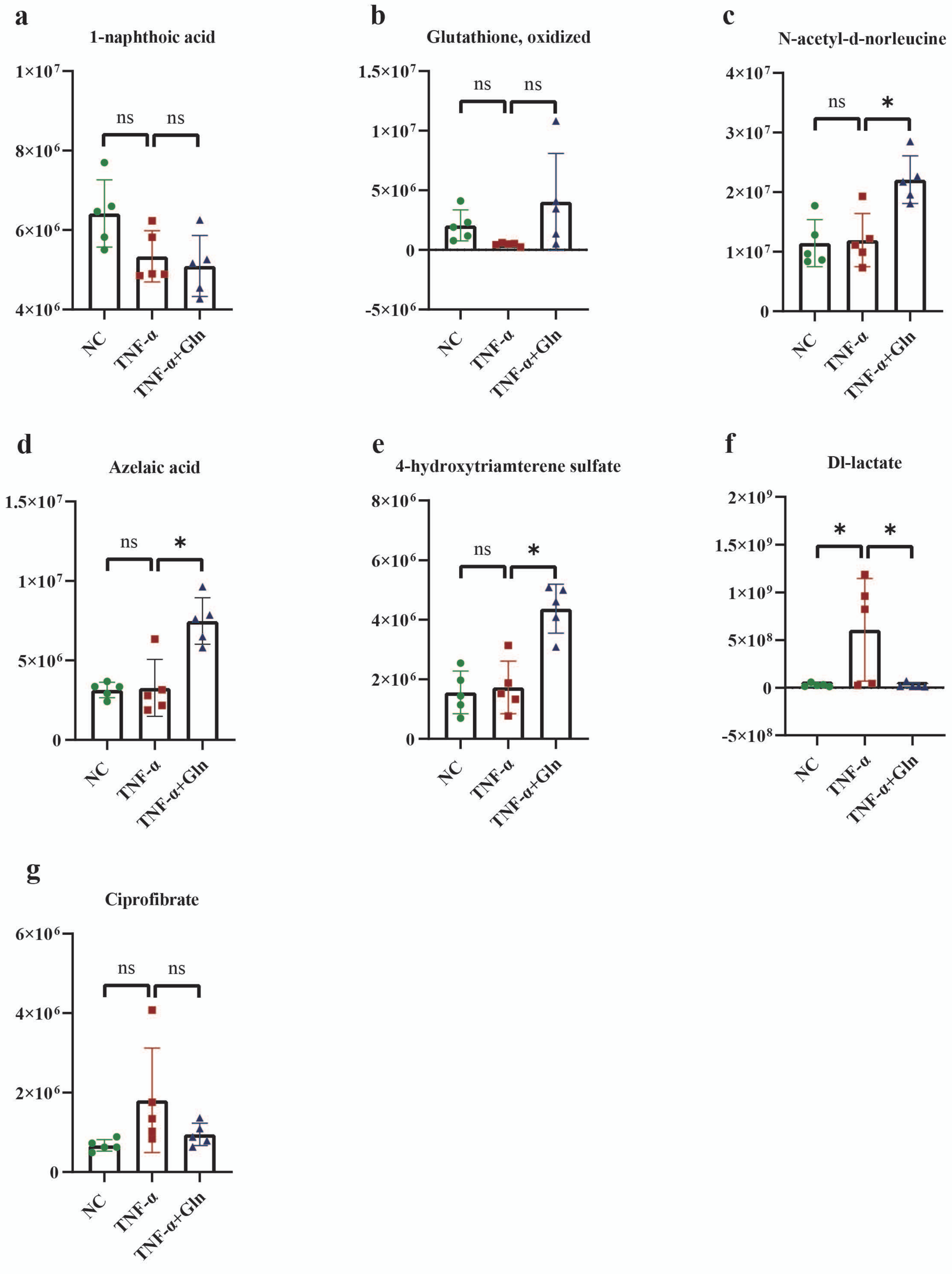

a
1-naphthoic acid
1×10^7
8×10^6
6×10^6
4×10^6
ns
ns
NC
TNF-α
TNF-α+Gln
b
Glutathione, oxidized
1.5×10^7
1×10^7
5×10^6
0
-5×10^6
ns
ns
NC
TNF-α
TNF-α+Gln
c
N-acetyl-d-norleucine
4×10^7
3×10^7
2×10^7
1×10^7
0
ns
*
NC
TNF-α
TNF-α+Gln
d
Azelaic acid
1.5×10^7
1×10^7
5×10^6
0
ns
*
NC
TNF-α
TNF-α+Gln
e
4-hydroxytriamterene sulfate
8×10^6
6×10^6
4×10^6
2×10^6
0
ns
*
NC
TNF-α
TNF-α+Gln
f
Dl-lactate
2×10^9
1.5×10^9
1×10^9
5×10^8
0
-5×10^8
*
*
NC
TNF-α
TNF-α+Gln
g
Ciprofibrate
6×10^6
4×10^6
2×10^6
0
ns
ns
NC
TNF-α
TNF-α+Gln

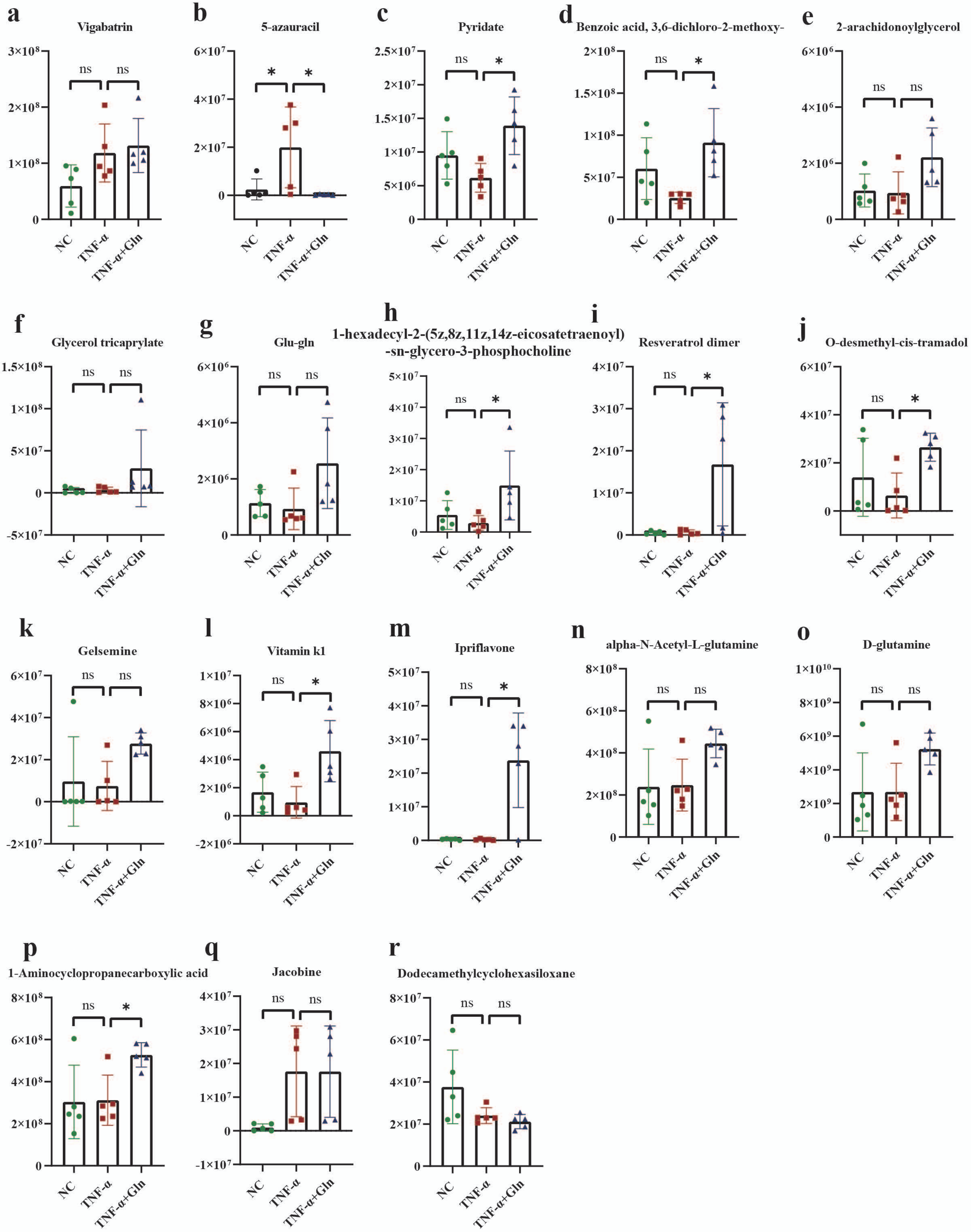

a
Vigabatrin
b
5-azauracil
c
Pyridate
d
Benzoic acid, 3,6-dichloro-2-methoxy-
e
2-arachidonoylglycerol
f
Glycerol tricaprylate
g
Glu-gln
h
1-hexadecyl-2-(5z,8z,11z,14z-eicosatetraenoyl)-sn-glycero-3-phosphocholine
i
Resveratrol dimer
j
O-desmethyl-cis-tramadol
k
Gelsemine
l
Vitamin k1
m
Ipriflavone
n
alpha-N-Acetyl-L-glutamine
o
D-glutamine
p
1-Aminocyclopropanecarboxylic acid
q
Jacobine
r
Dodecamethylcyclohexasiloxane
NC
TNF-α
TNF-α+Gln
ns
*

Fig 1h

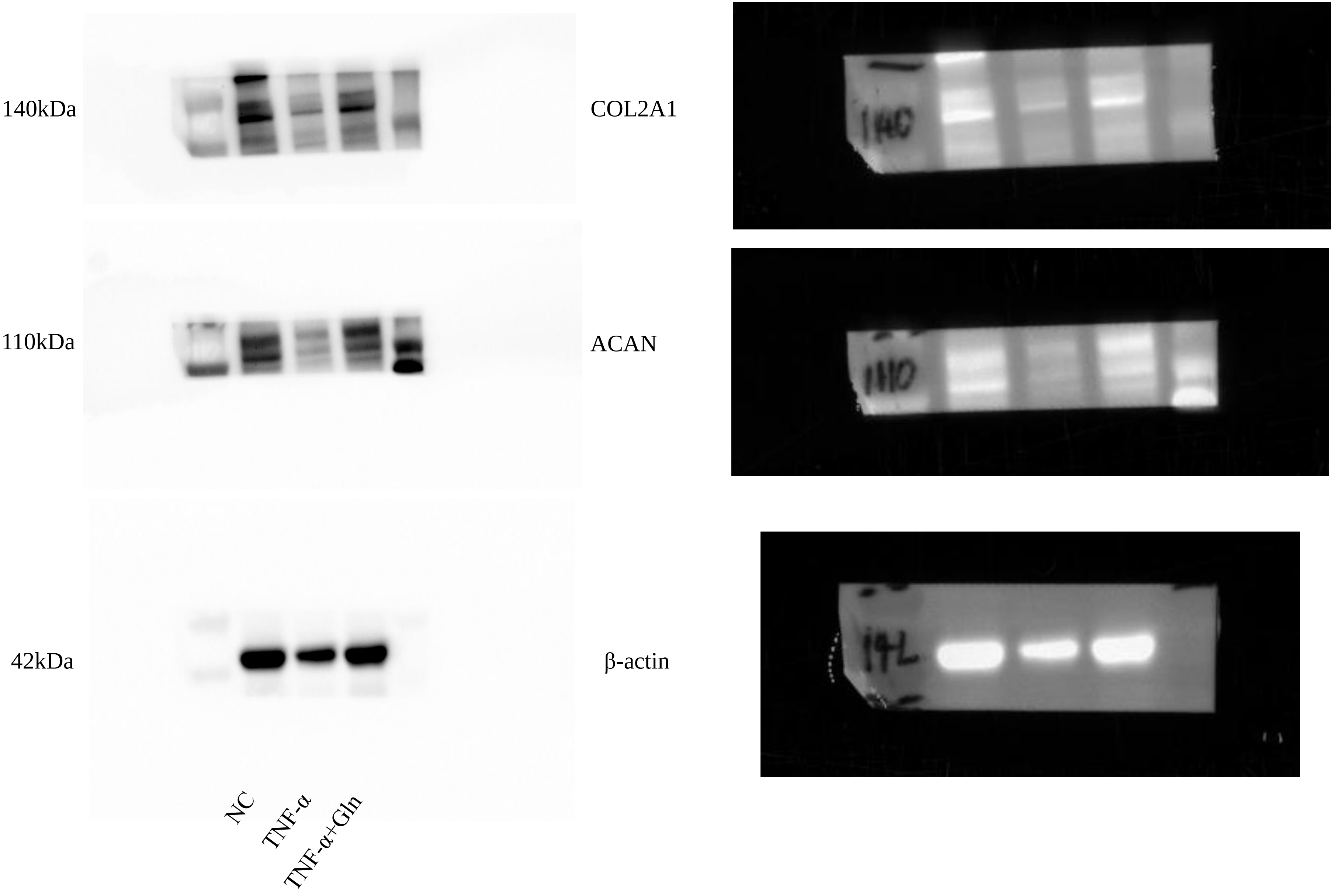

Fig 1h

75kDa

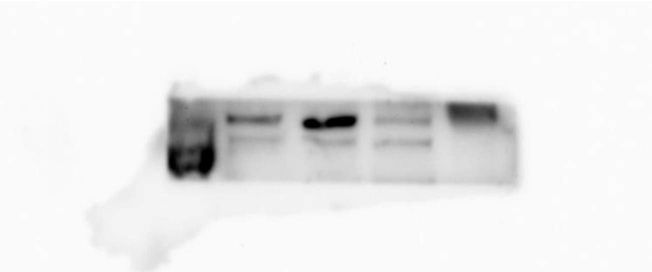

ADAMTS5

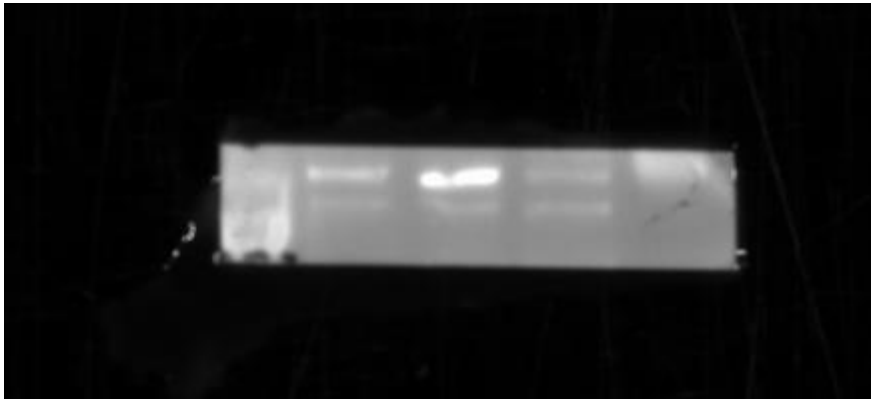

54kDa

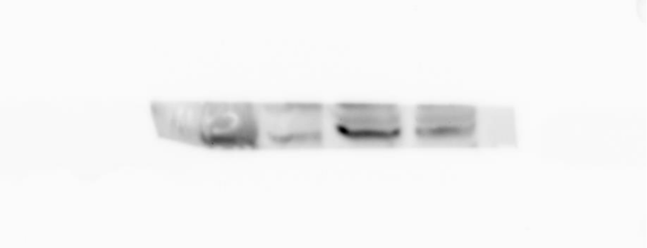

MMP3

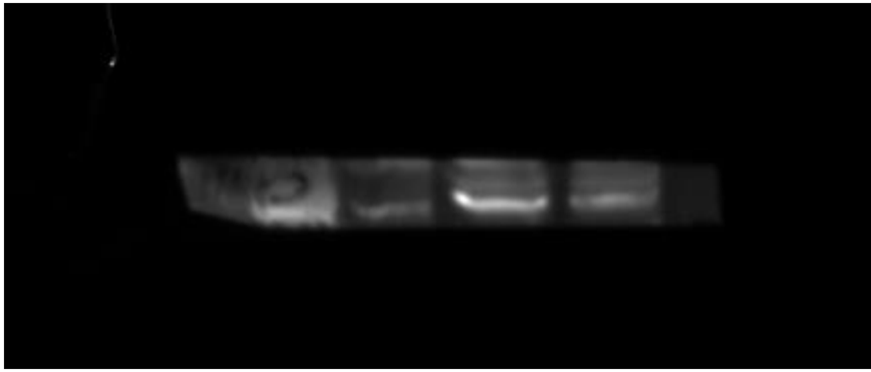

42kDa

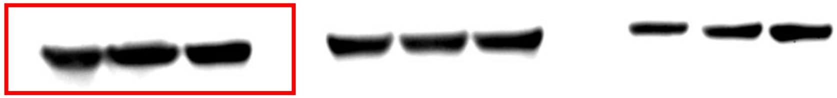

β-actin

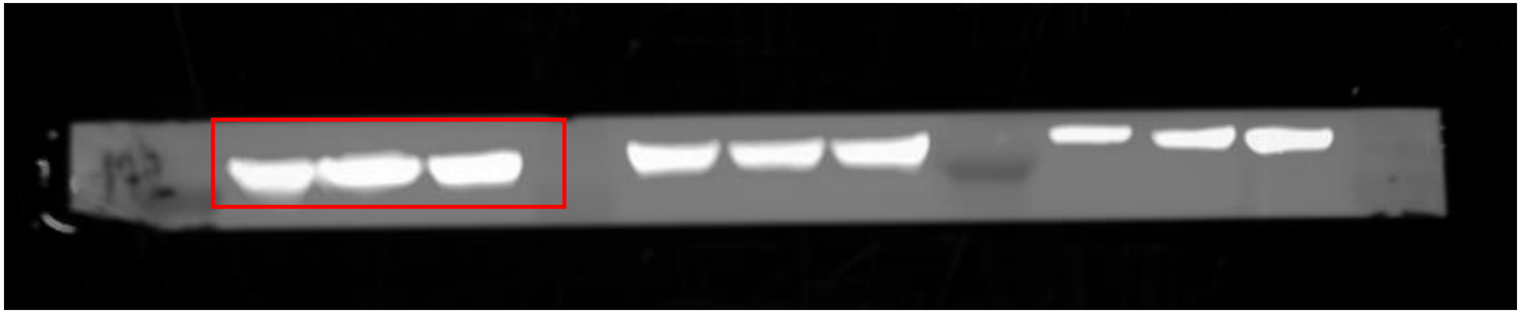

Fig 1j

62kDa

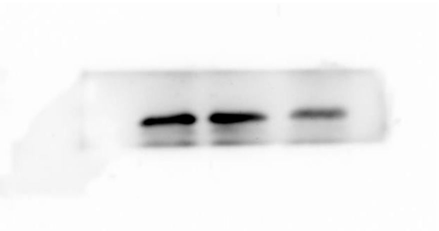

p62

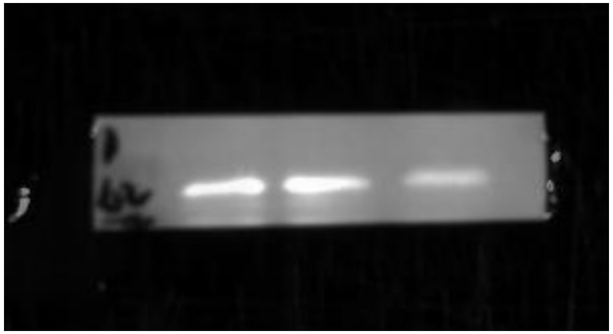

16kDa
14kDa

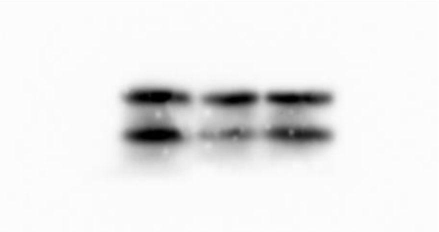

LC3 I
LC3 II

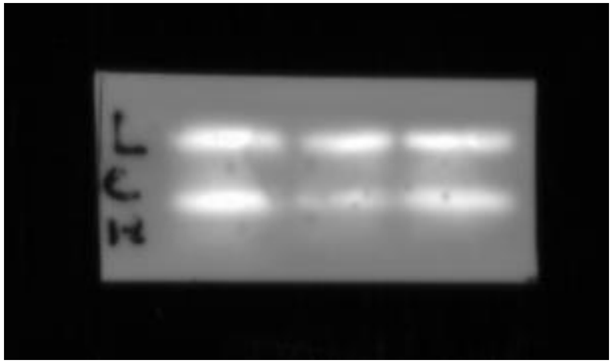

42kDa

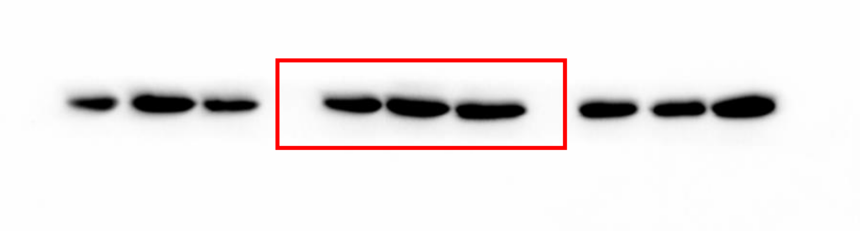

β-actin

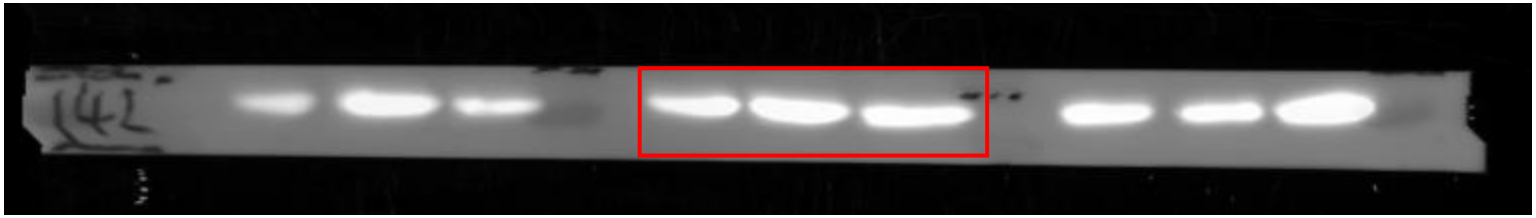

Fig 1j

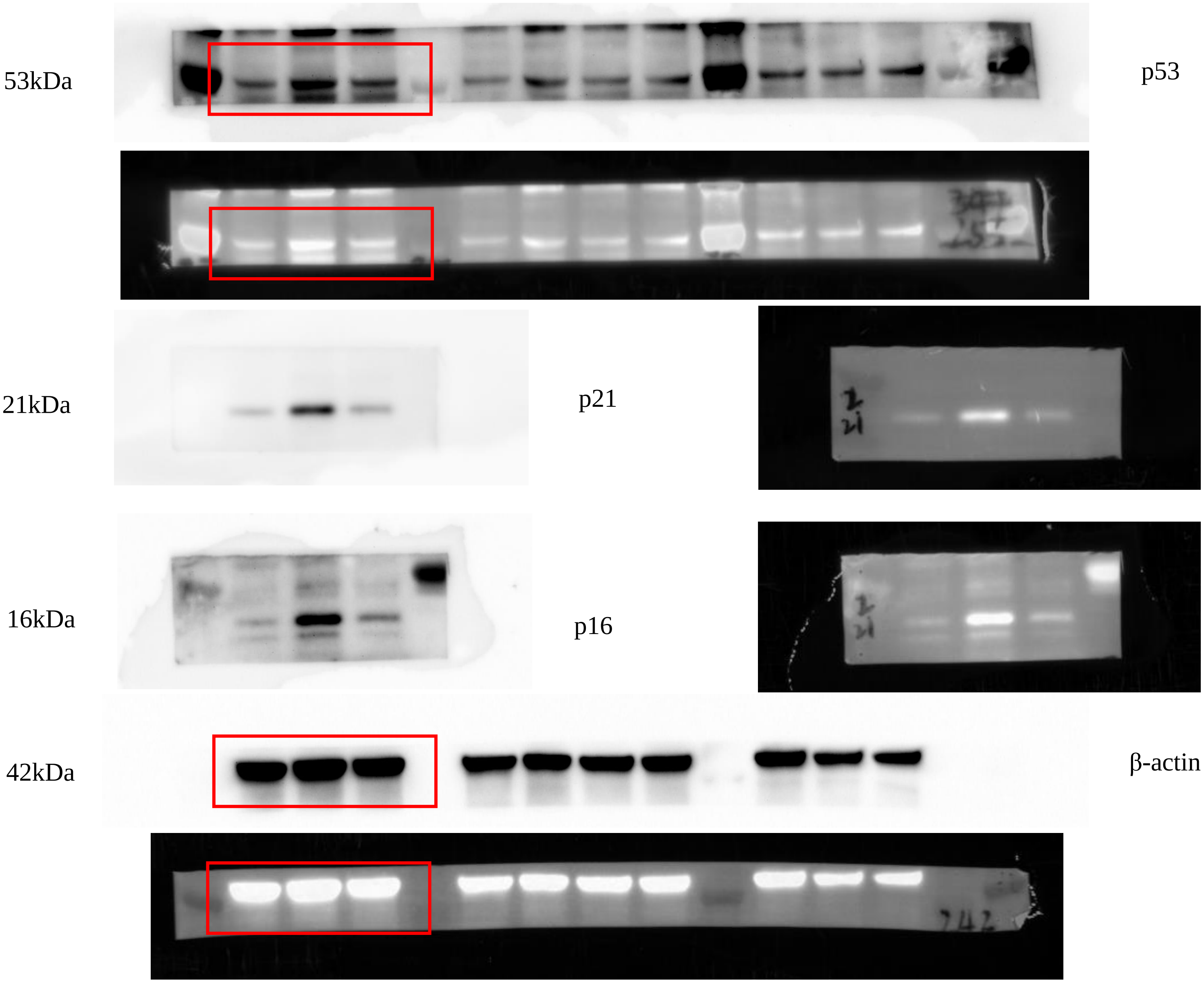

Fig 2c

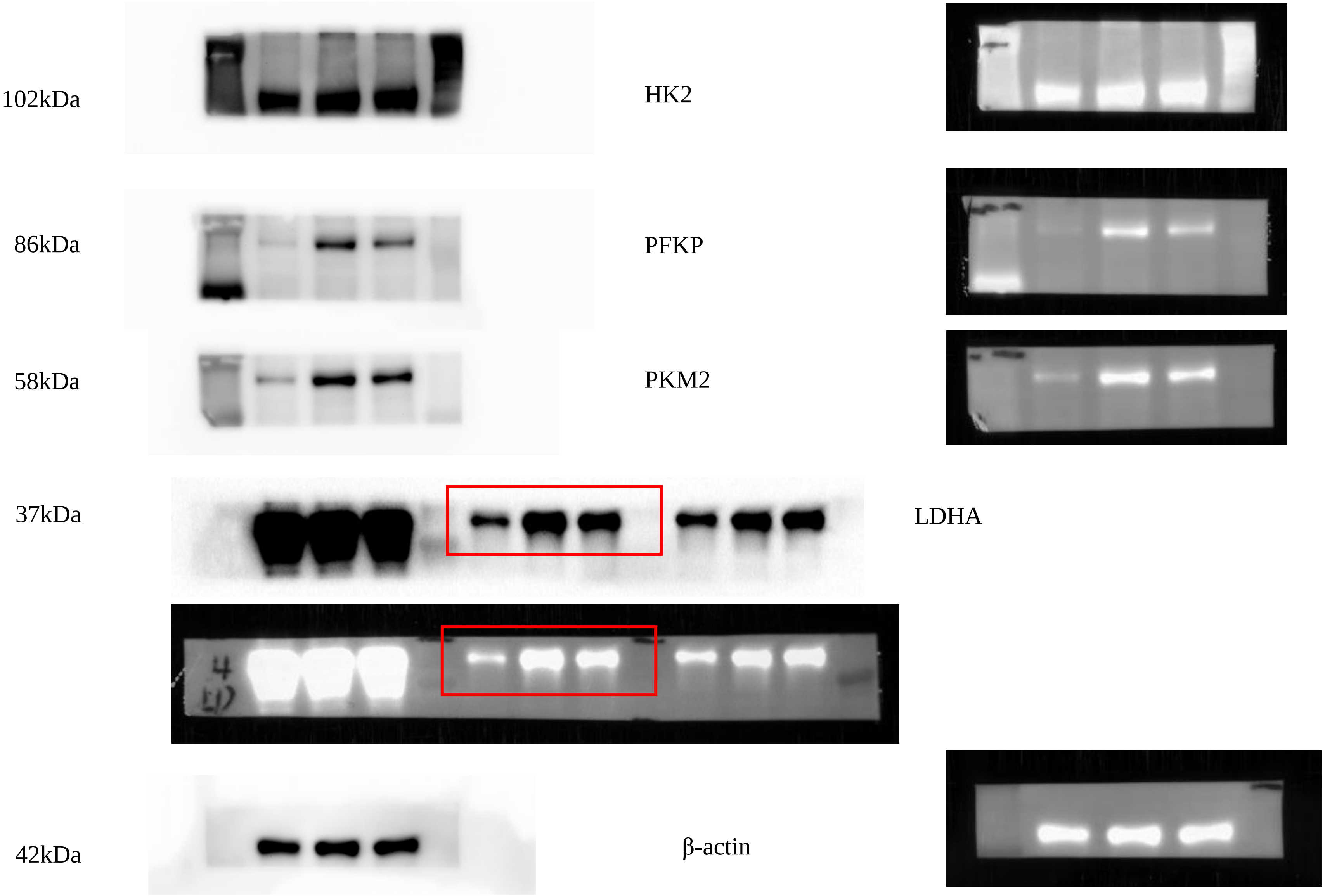

Fig 2f

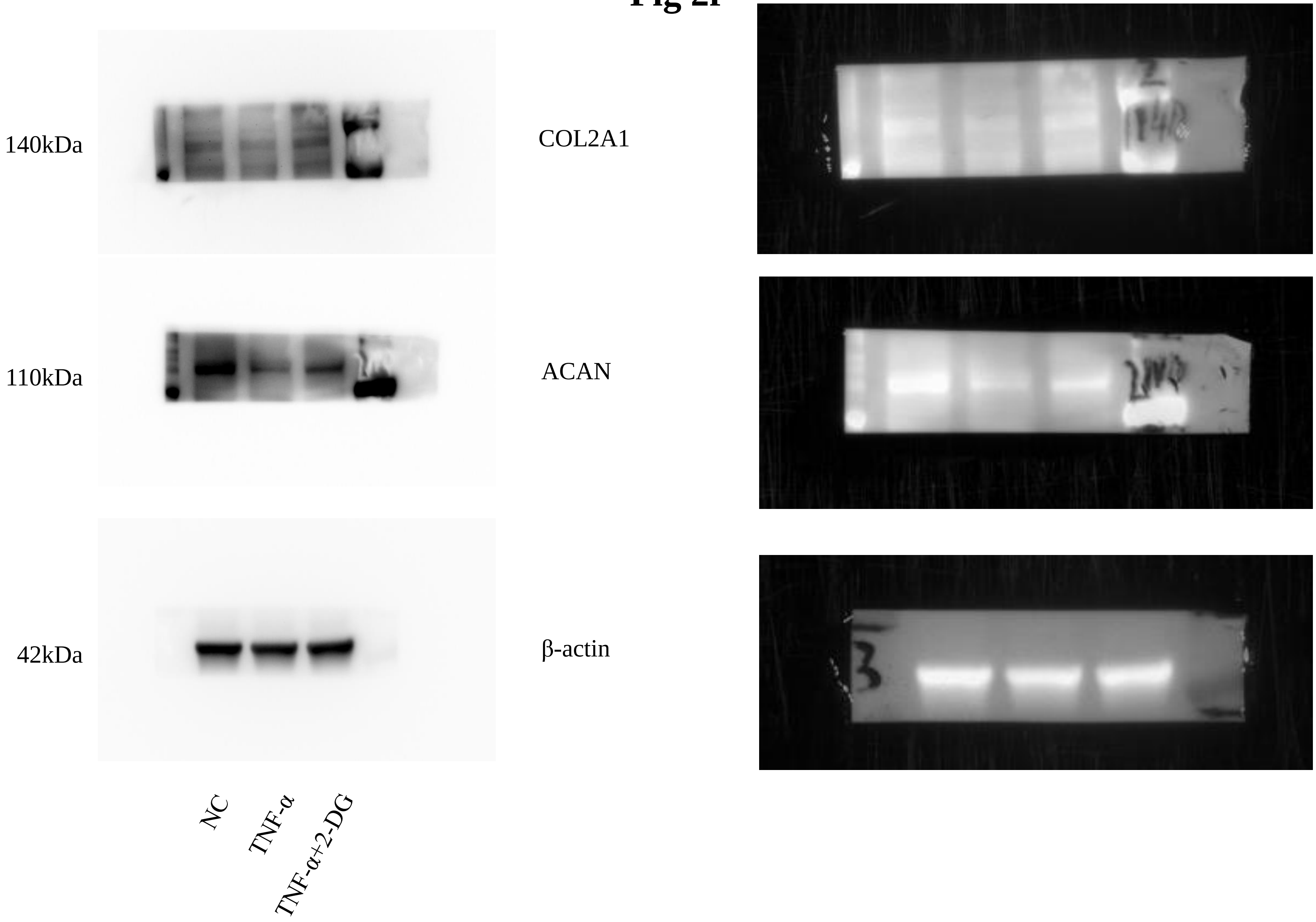

Fig 2f

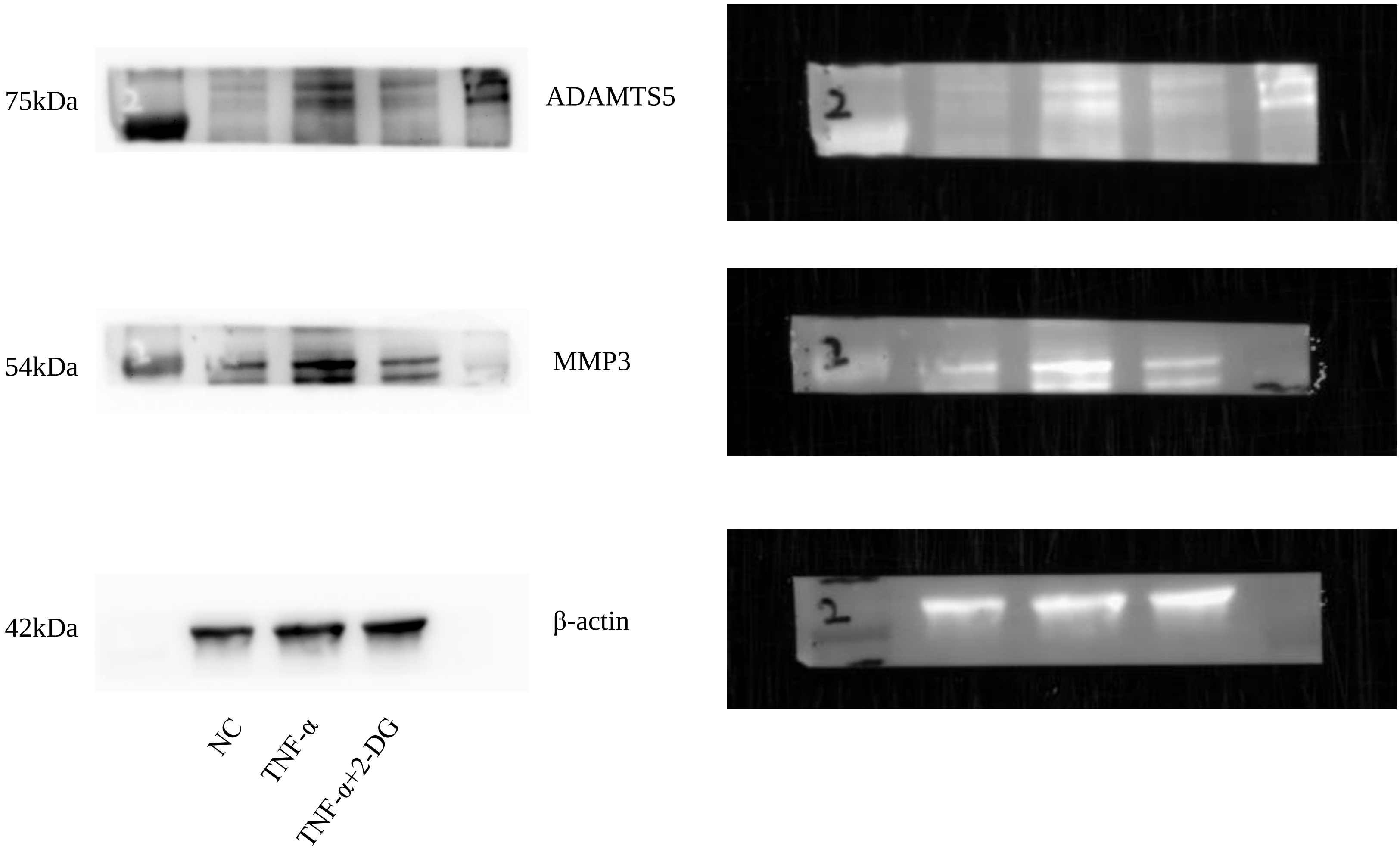

Fig 2g

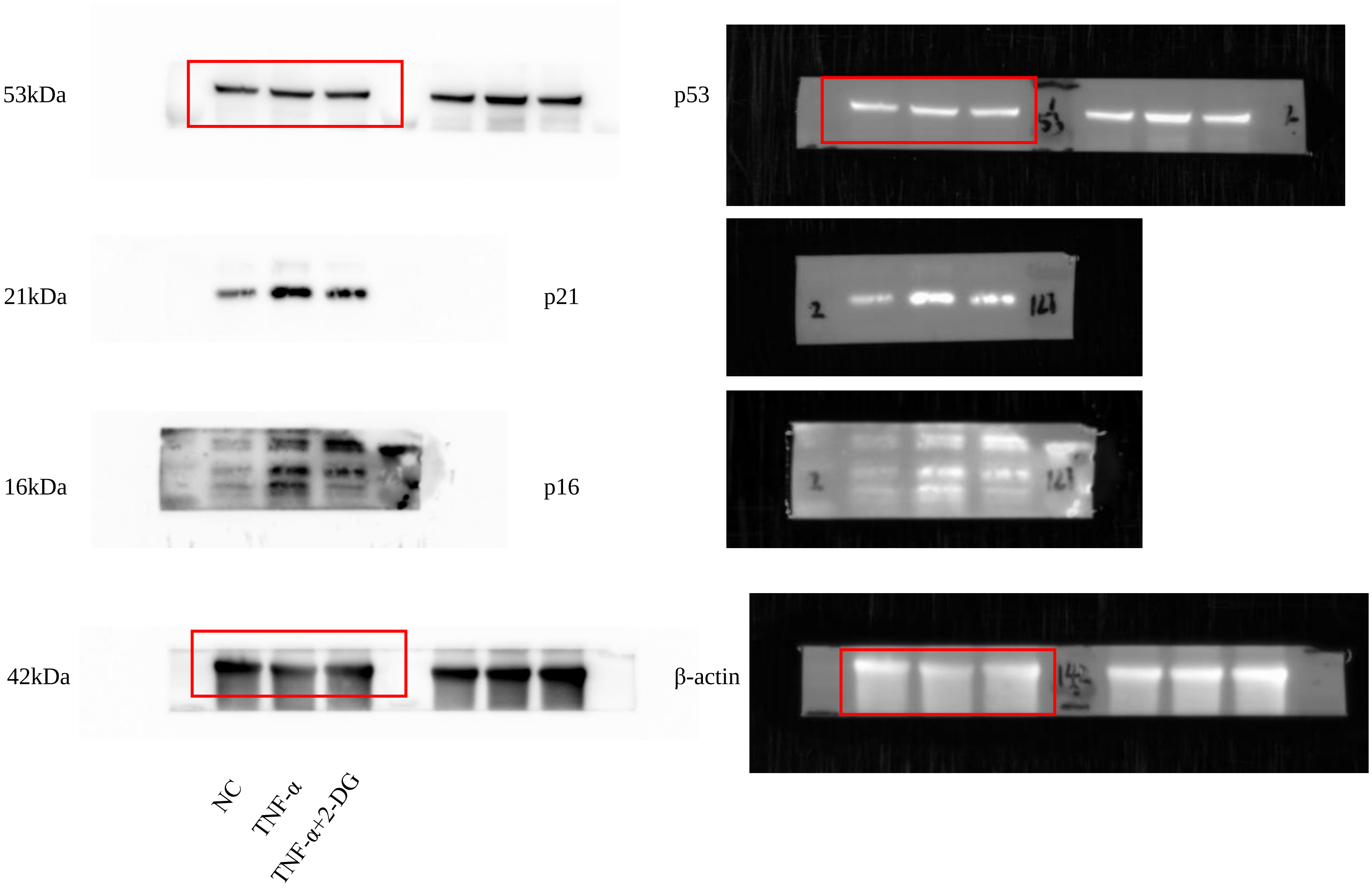

Fig 2i

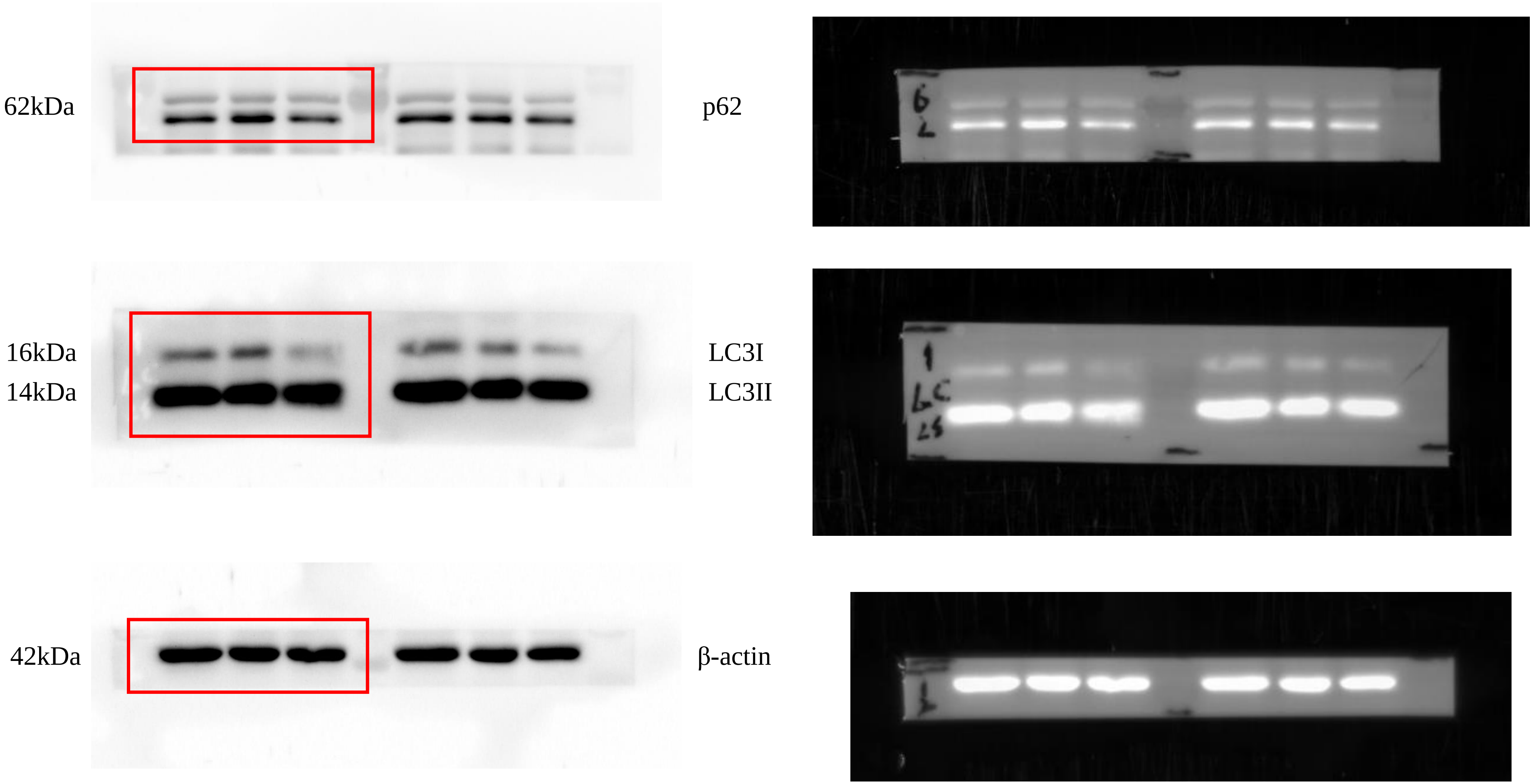

Fig 3g

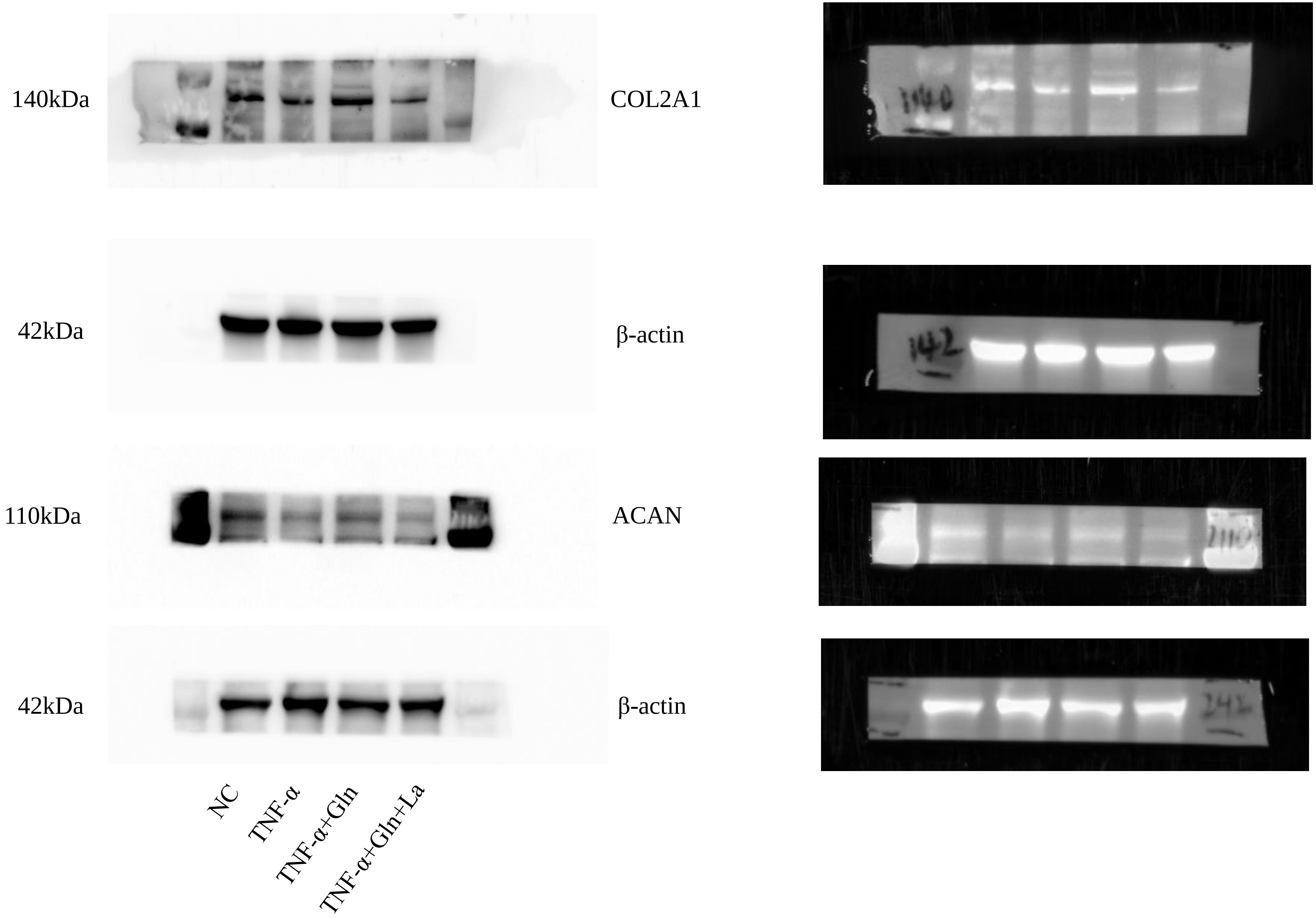

140kDa
COL2A1
42kDa
β-actin
110kDa
ACAN
42kDa
β-actin
NC
TNF-α
TNF-α+Gln
TNF-α+Gln+La

Fig 3h

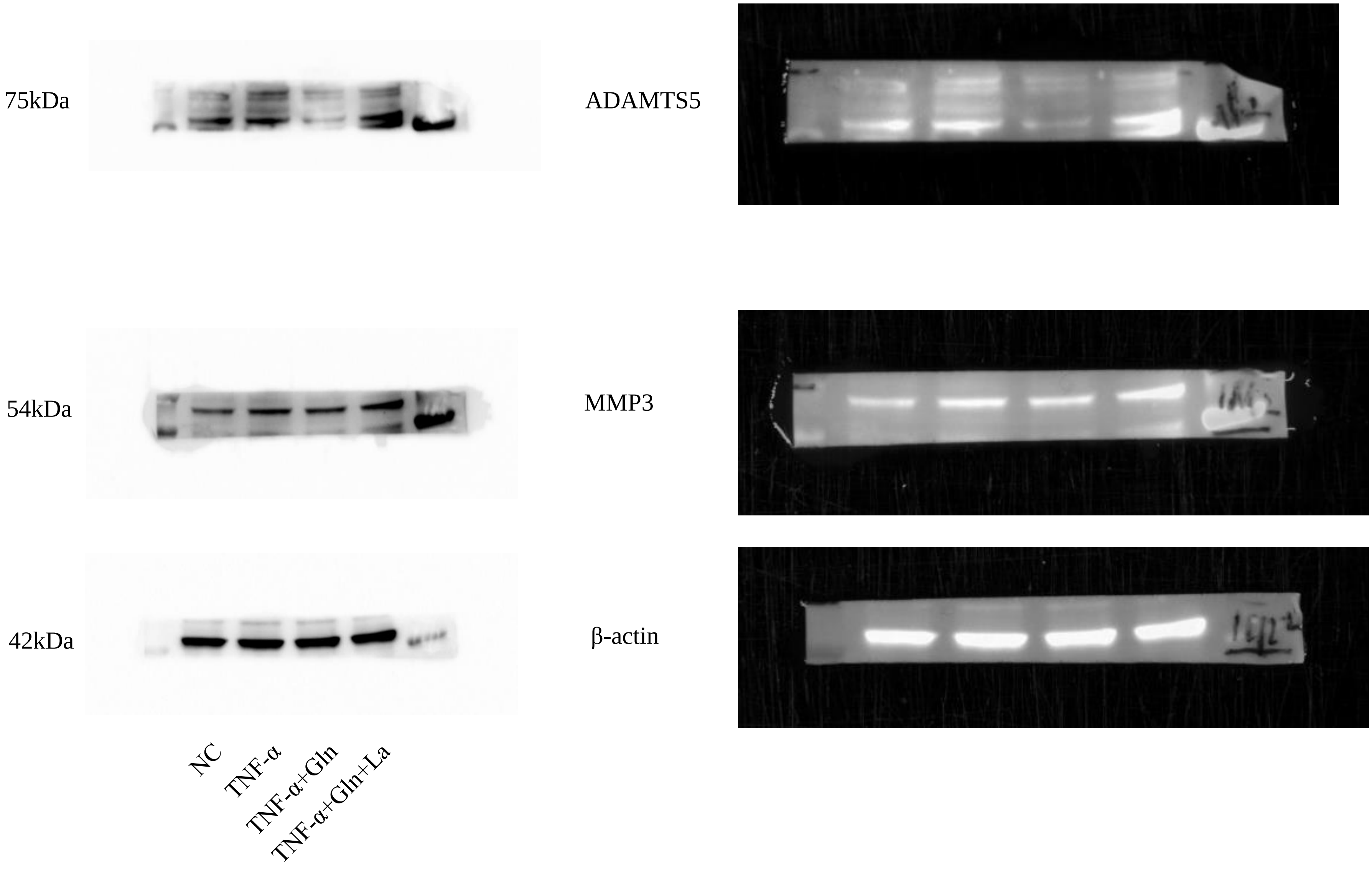
75kDa
ADAMTS5
54kDa
MMP3
42kDa
β-actin
NC
TNF-α
TNF-α+Gln
TNF-α+Gln+La

Fig 3j

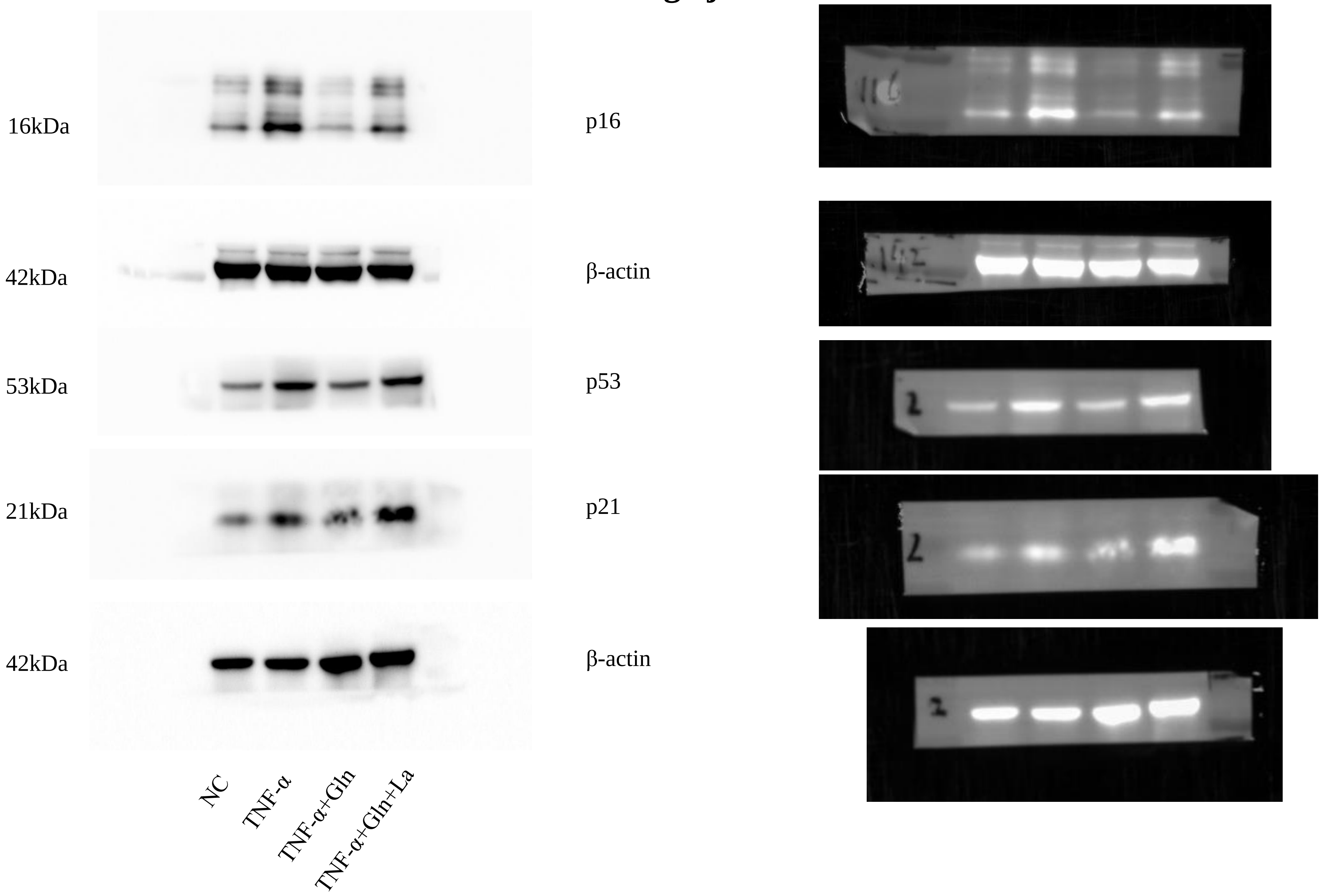
16kDa
p16
42kDa
β-actin
53kDa
p53
21kDa
p21
42kDa
β-actin
NC
TNF-α
TNF-α+Gln
TNF-α+Gln+La

Fig 3k

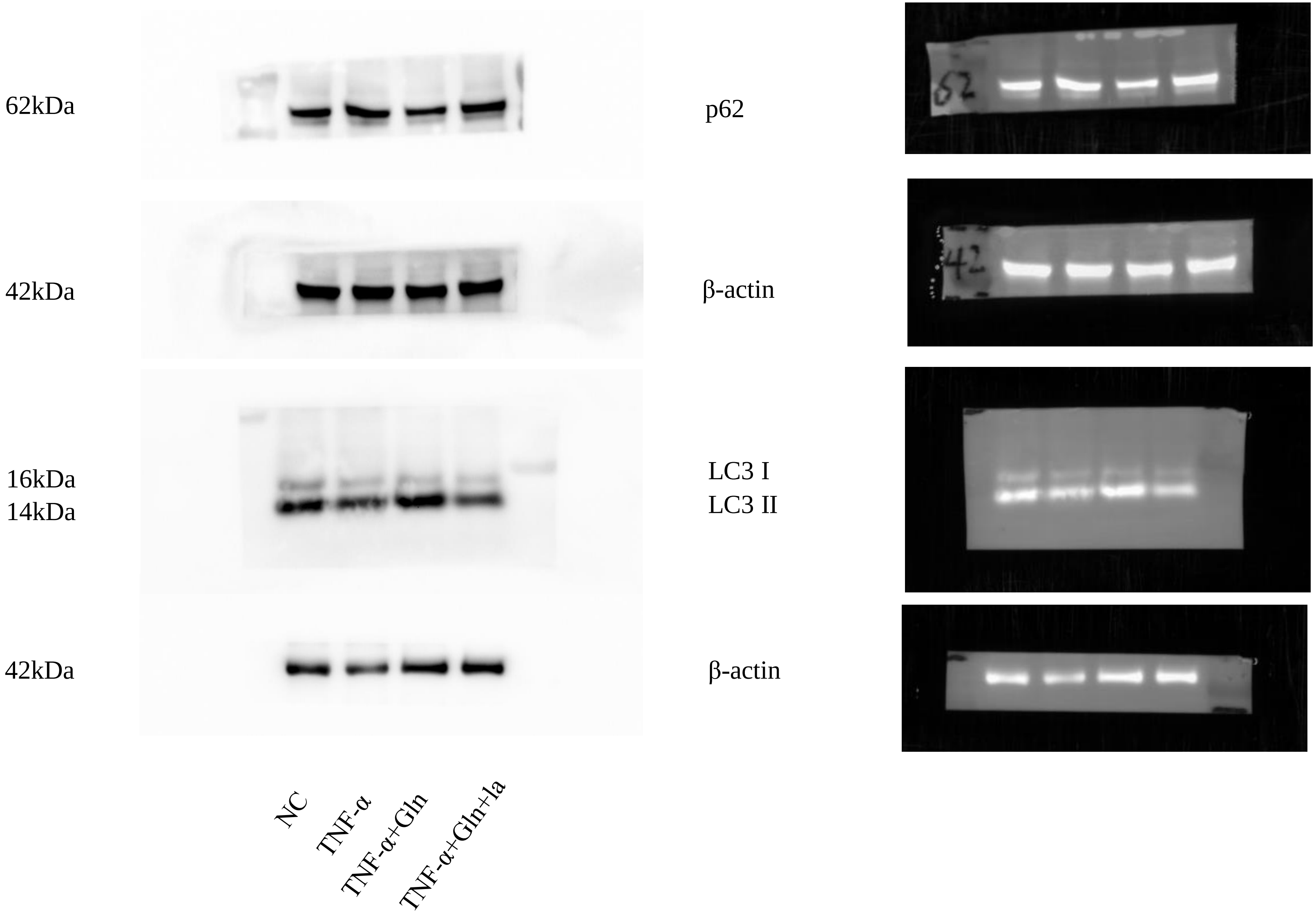
62kDa
p62
42kDa
β-actin
16kDa
14kDa
LC3 I
LC3 II
42kDa
β-actin
NC
TNF-α
TNF-α+Gln
TNF-α+Gln+la

Fig 4a

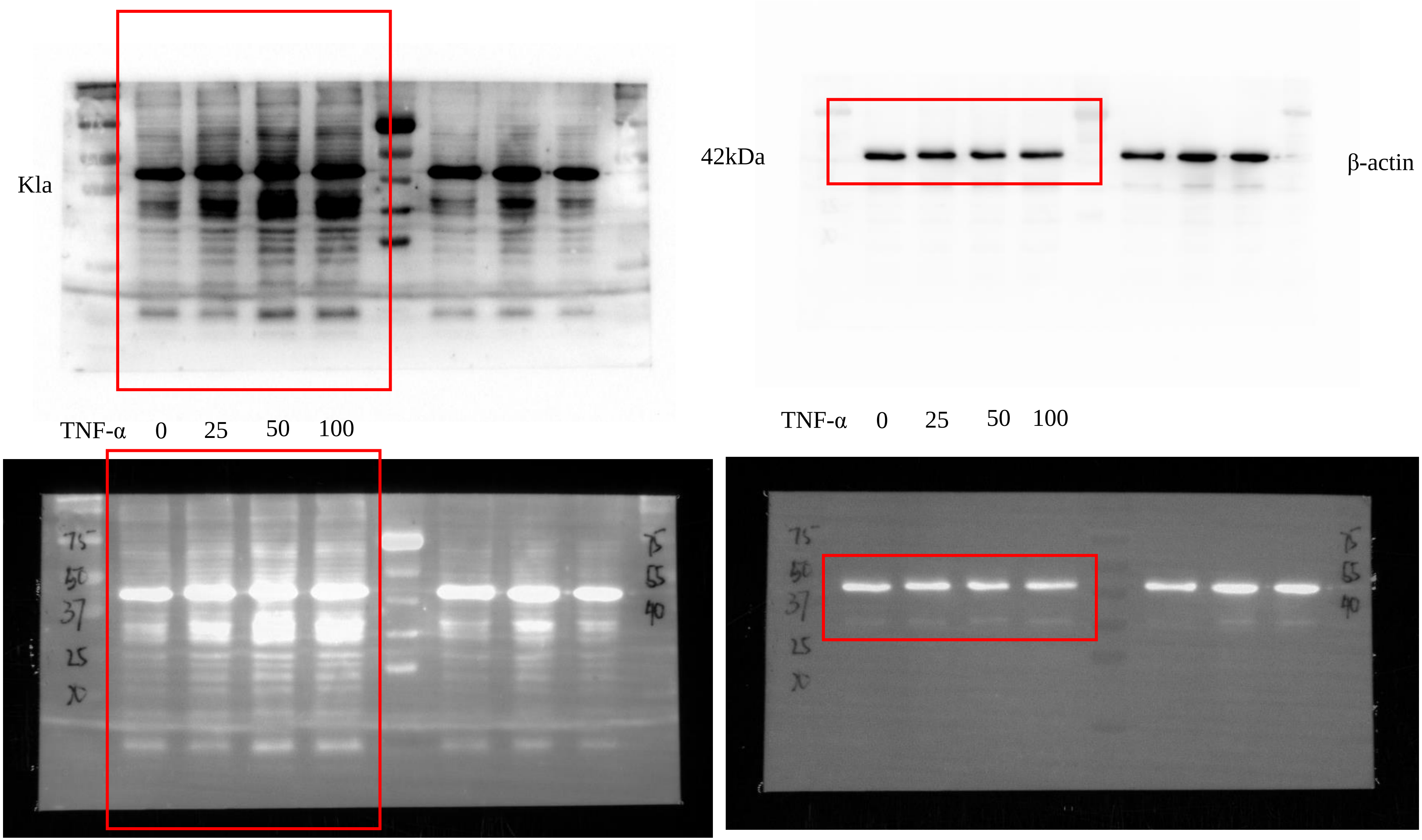

Fig 4b

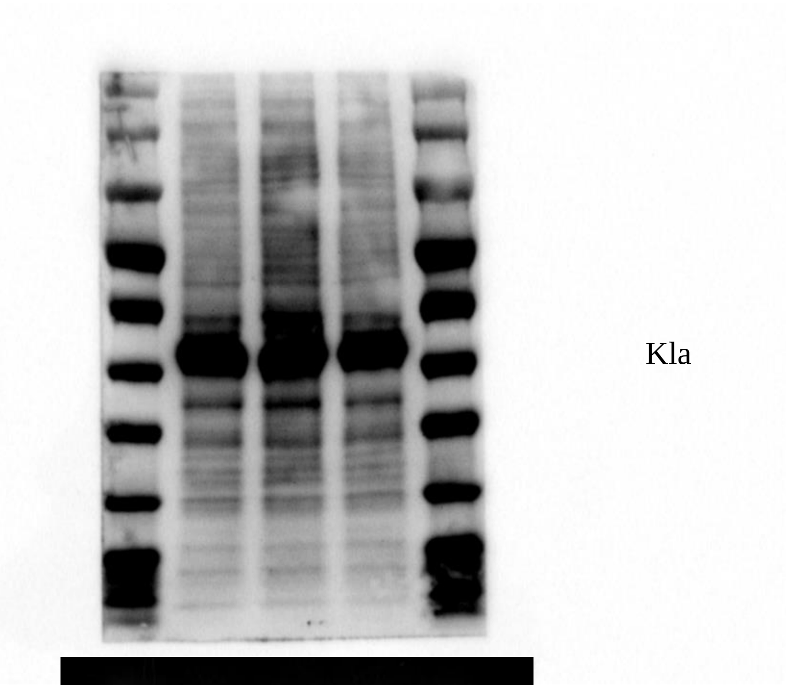

Kla

42kDa

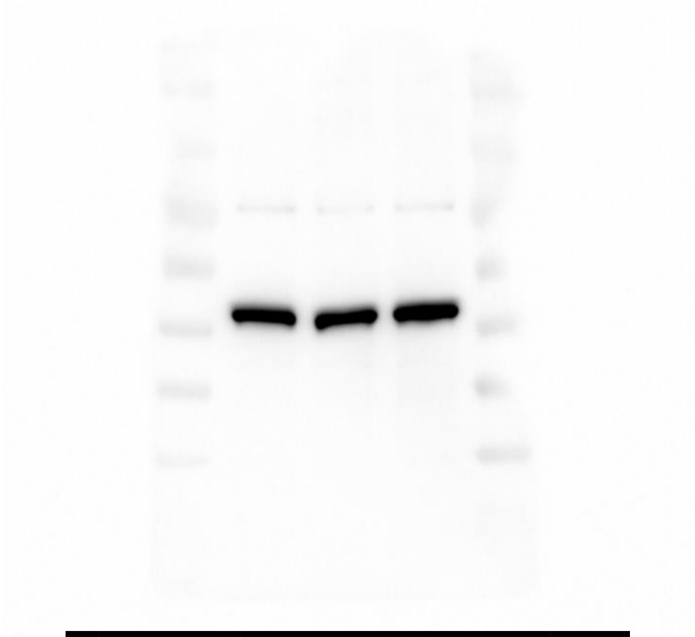

β-actin

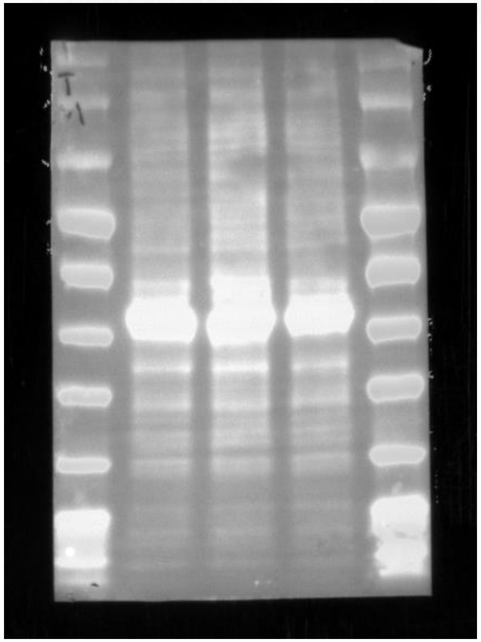

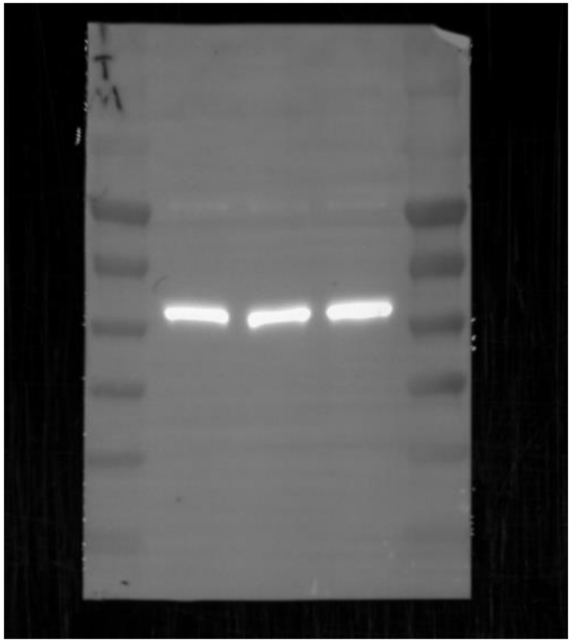

Fig 4c

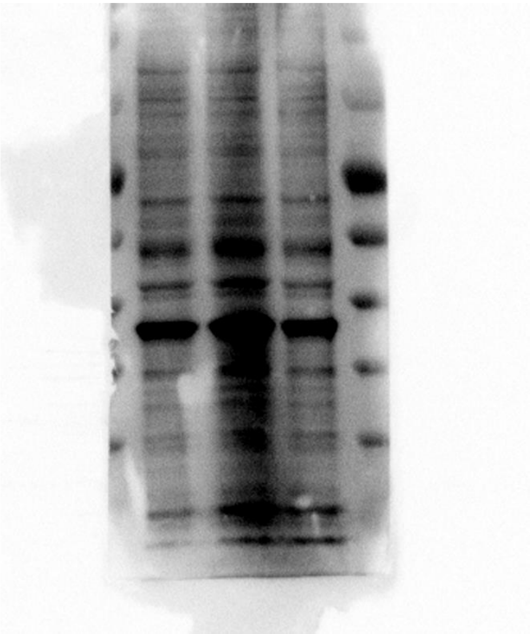

Kla

42kDa

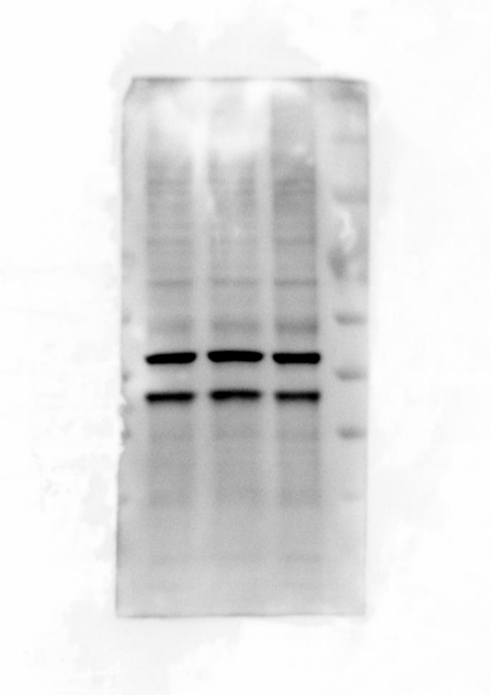

β-actin

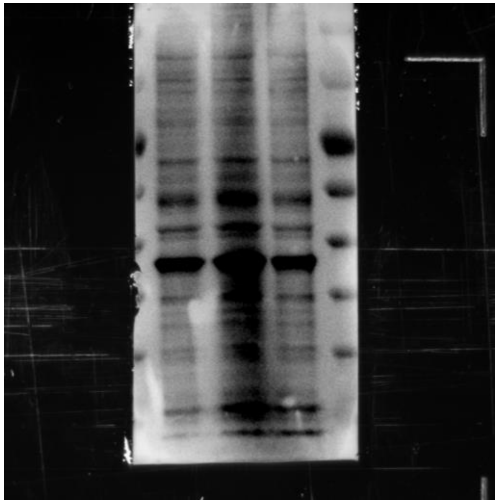

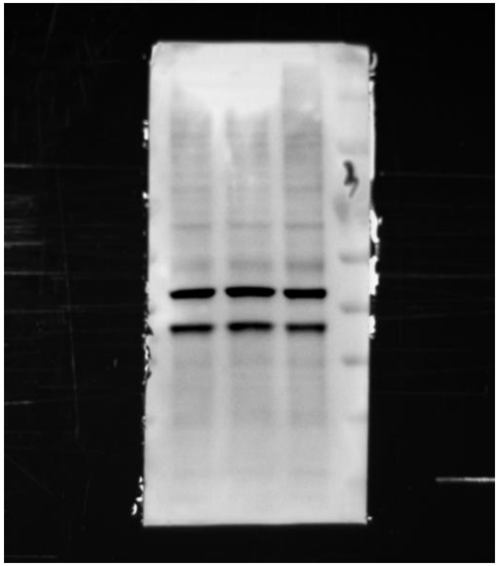

Fig 4d

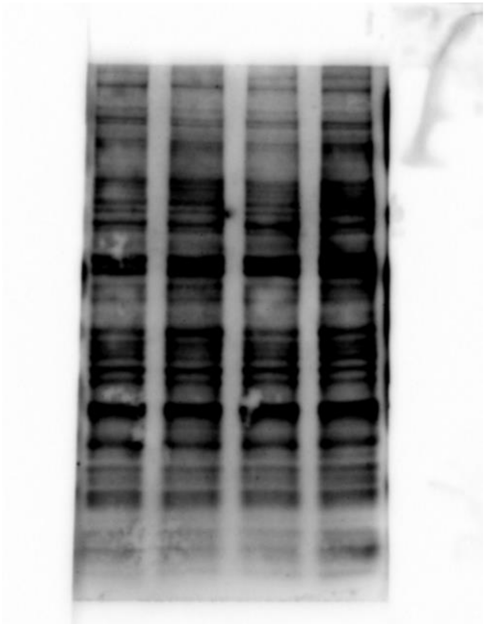

Kla

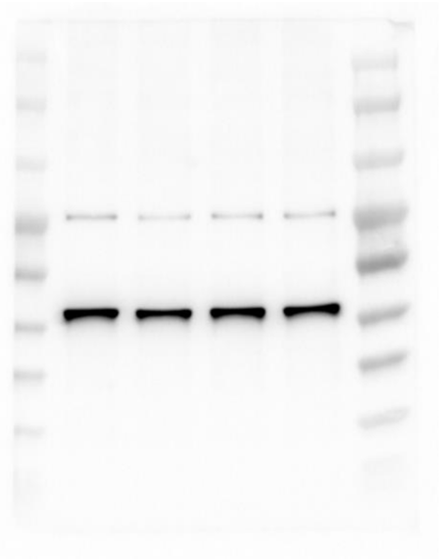

42kDa

β-actin

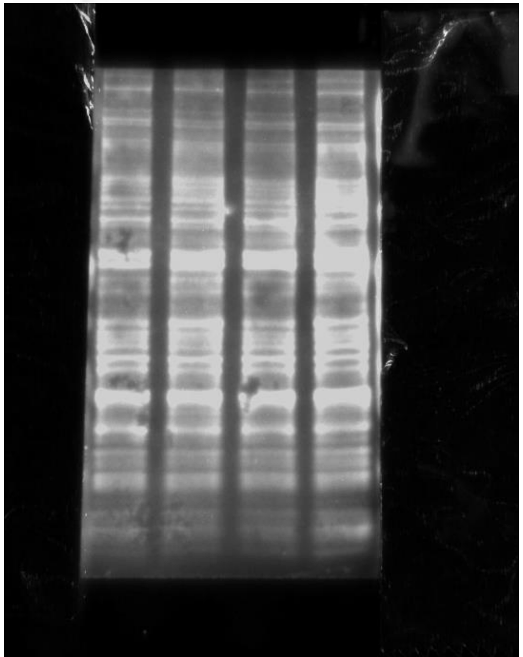

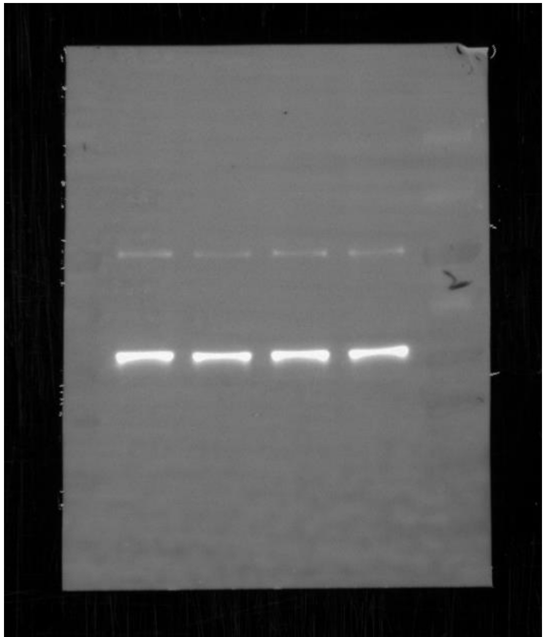

Fig 4d

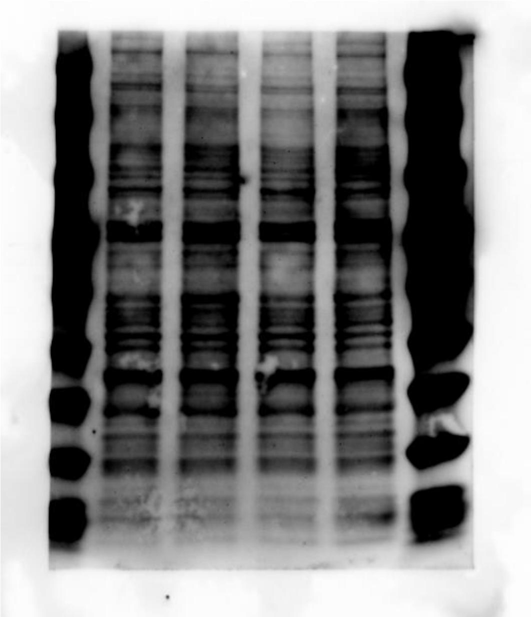

Kla

42kDa

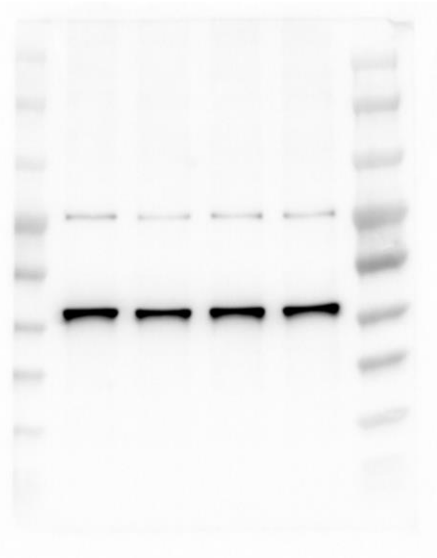

β-actin

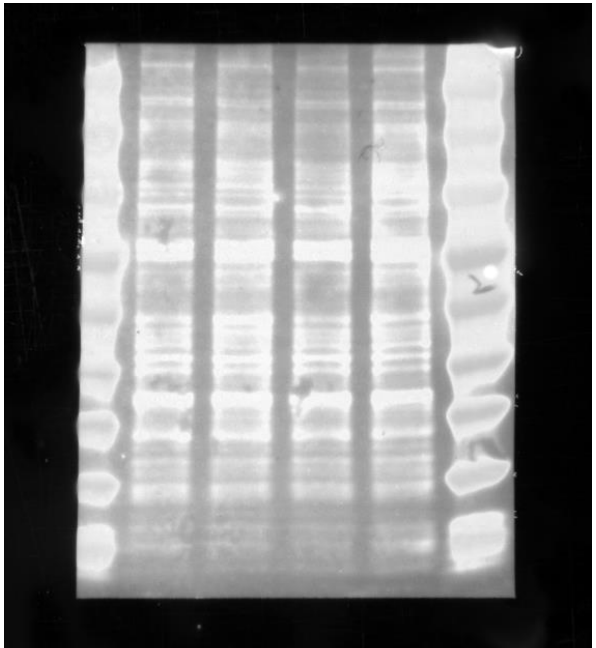

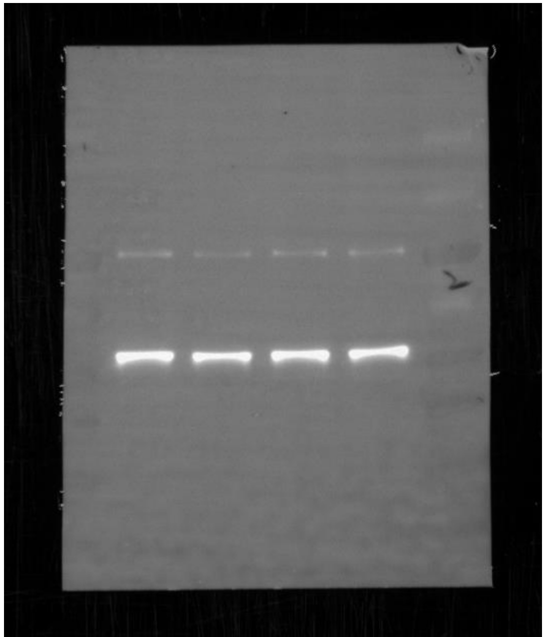

Fig 4e

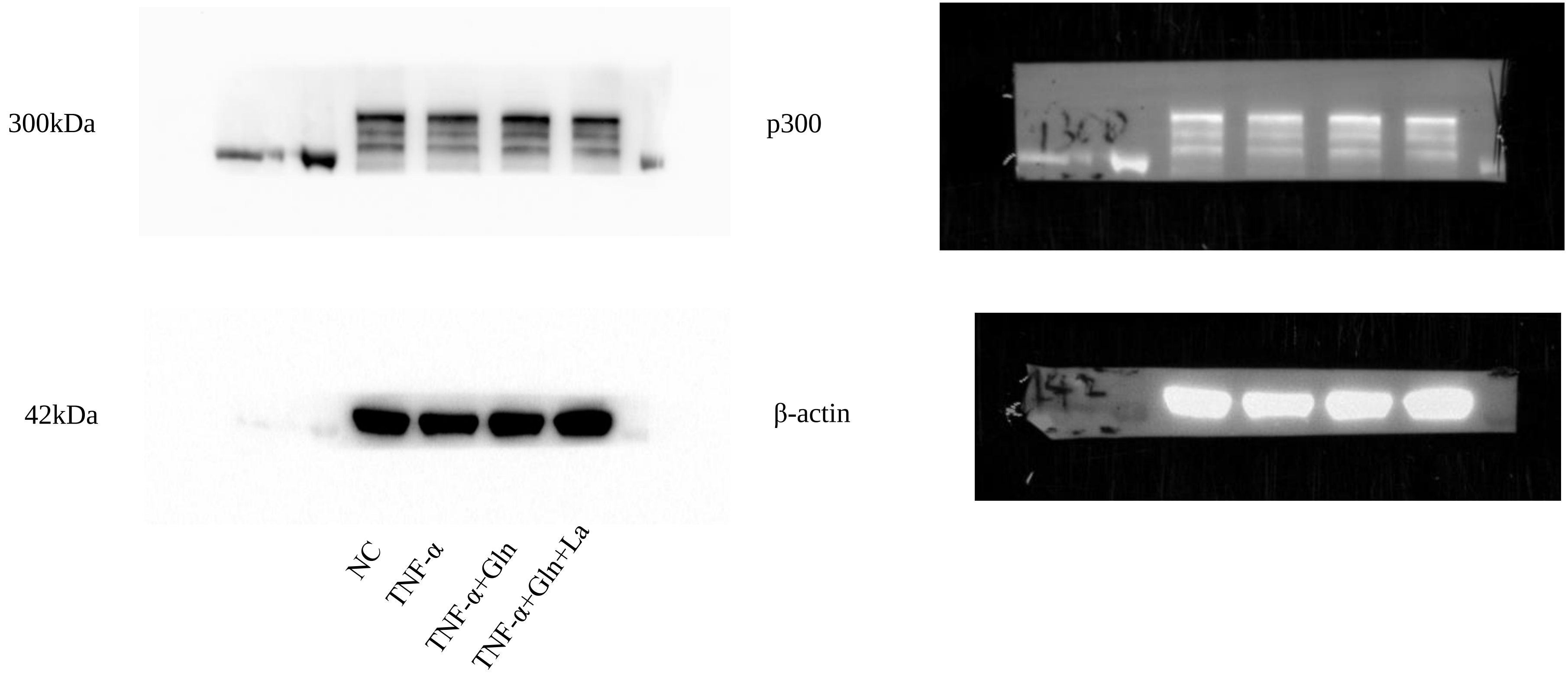

Fig 5a

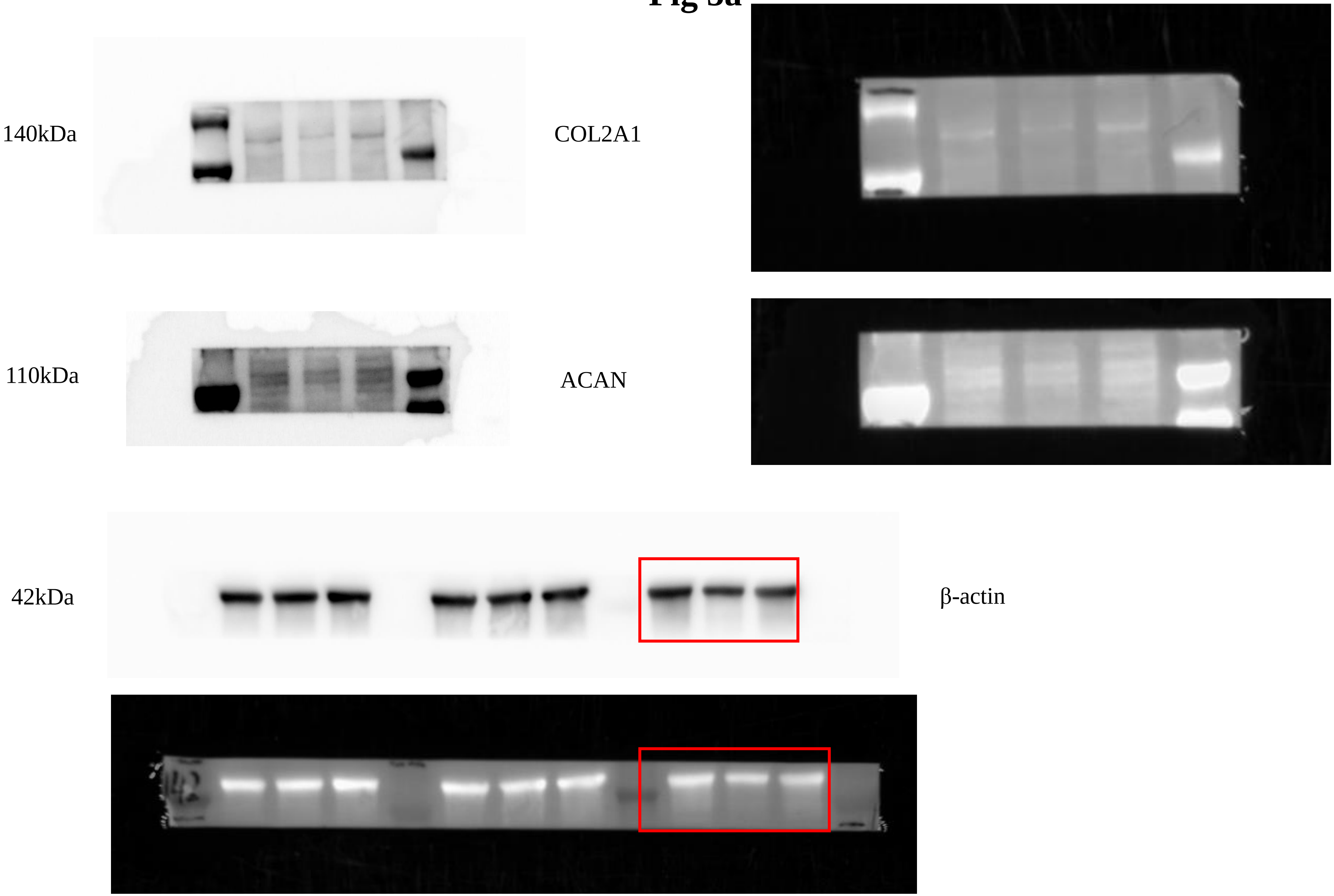

Fig 5a

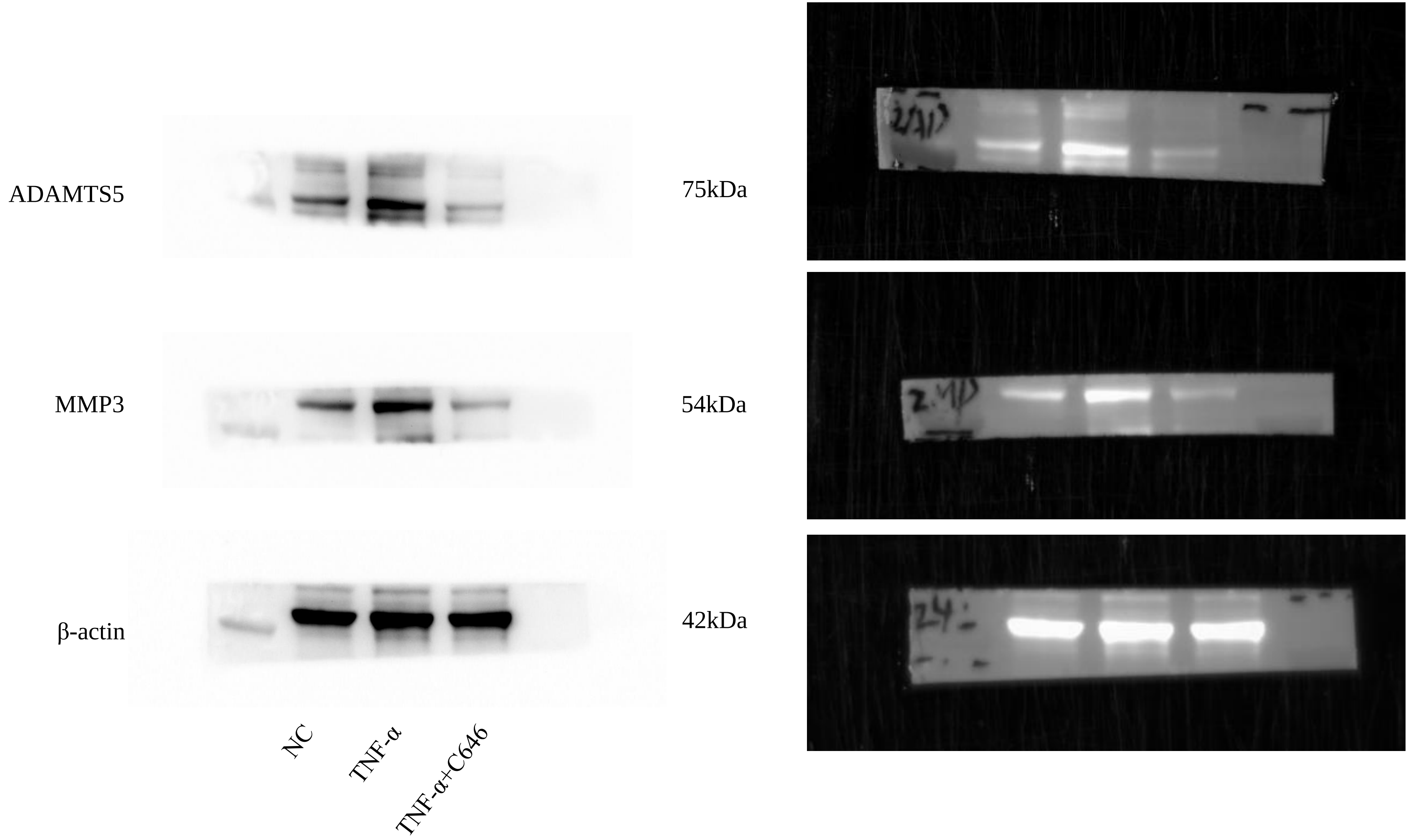

Fig 5c

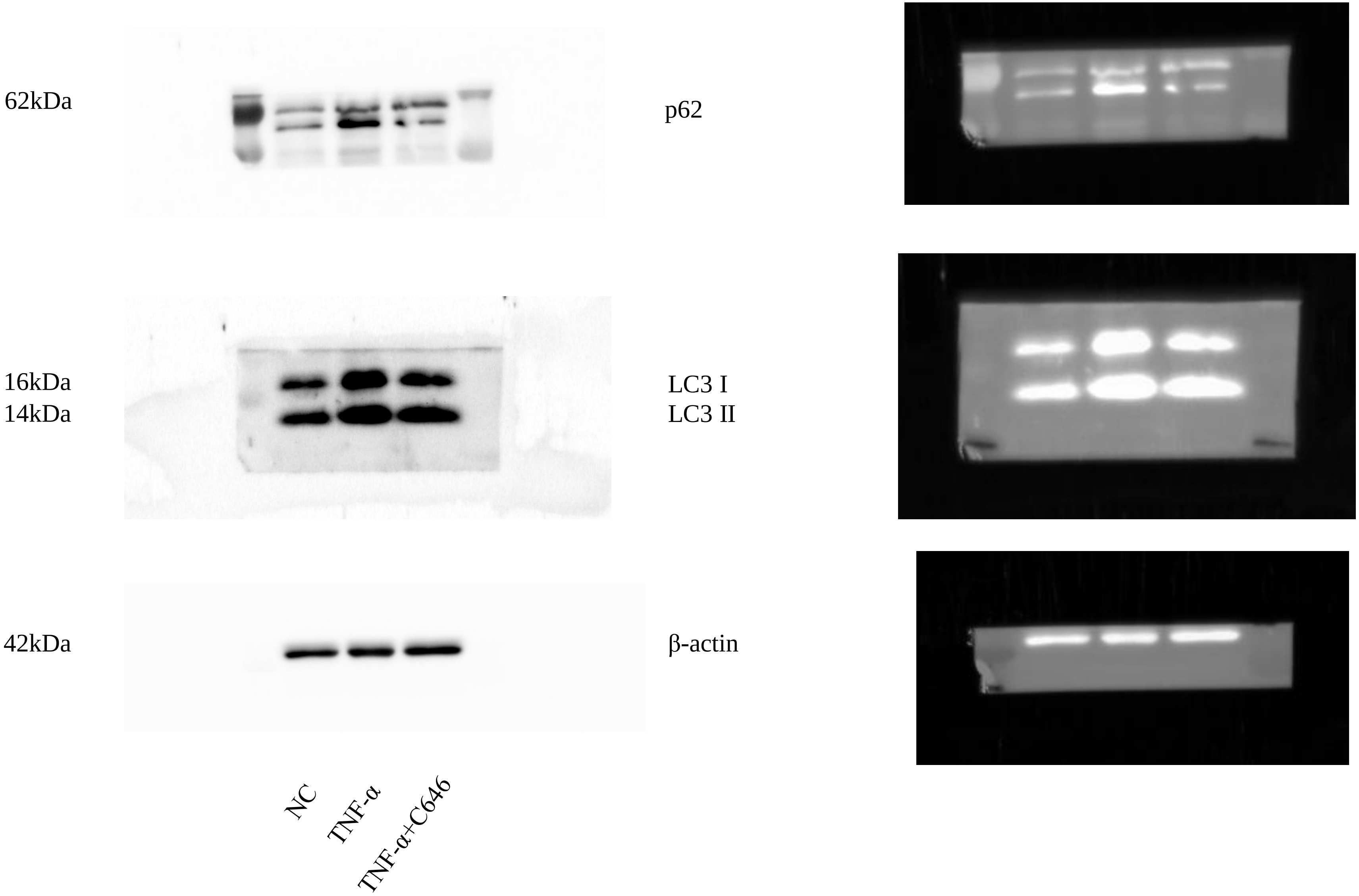
62kDa
p62
16kDa
14kDa
LC3 I
LC3 II
42kDa
β-actin
NC
TNF-α
TNF-α+C646

Fig 5e

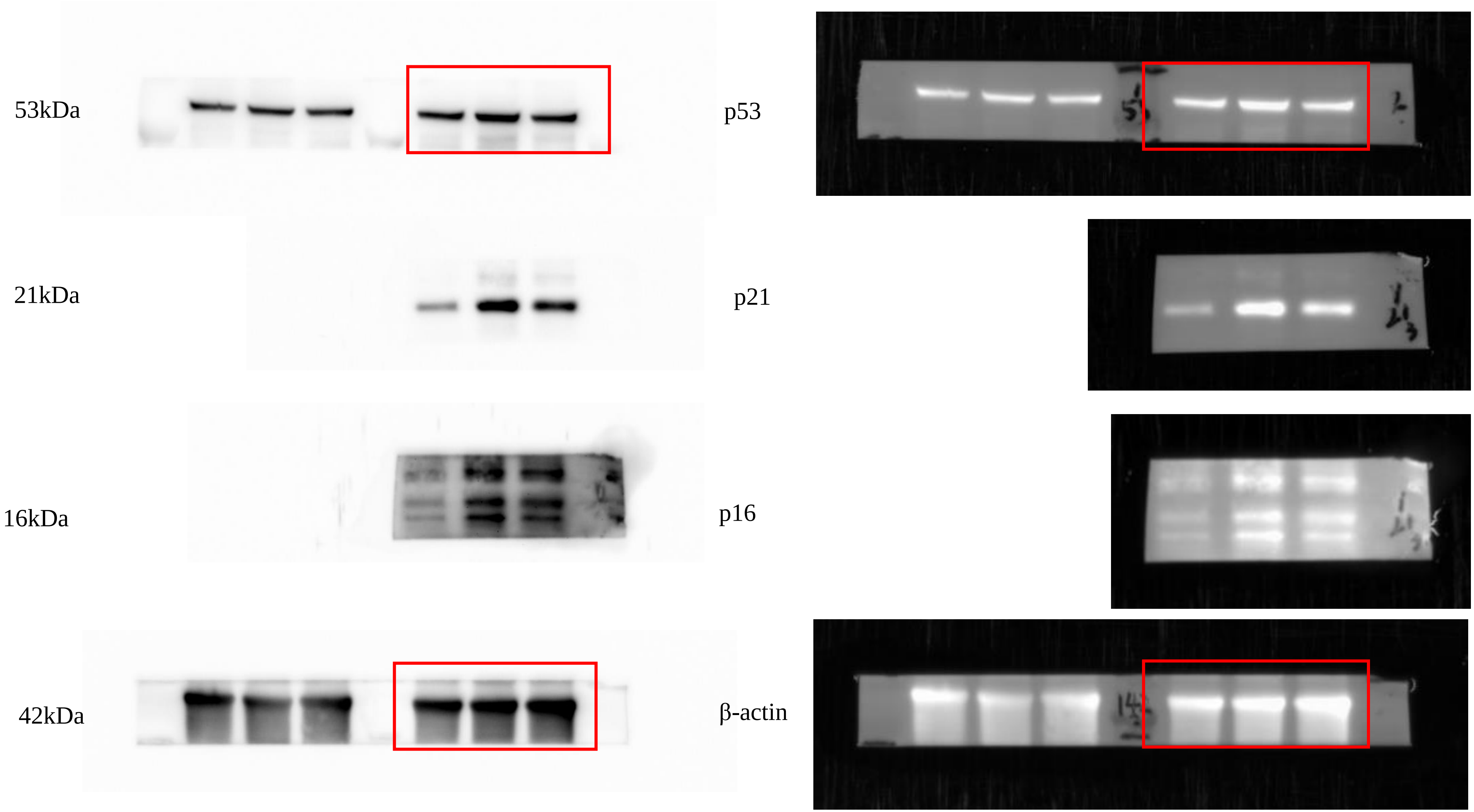

Fig 5h

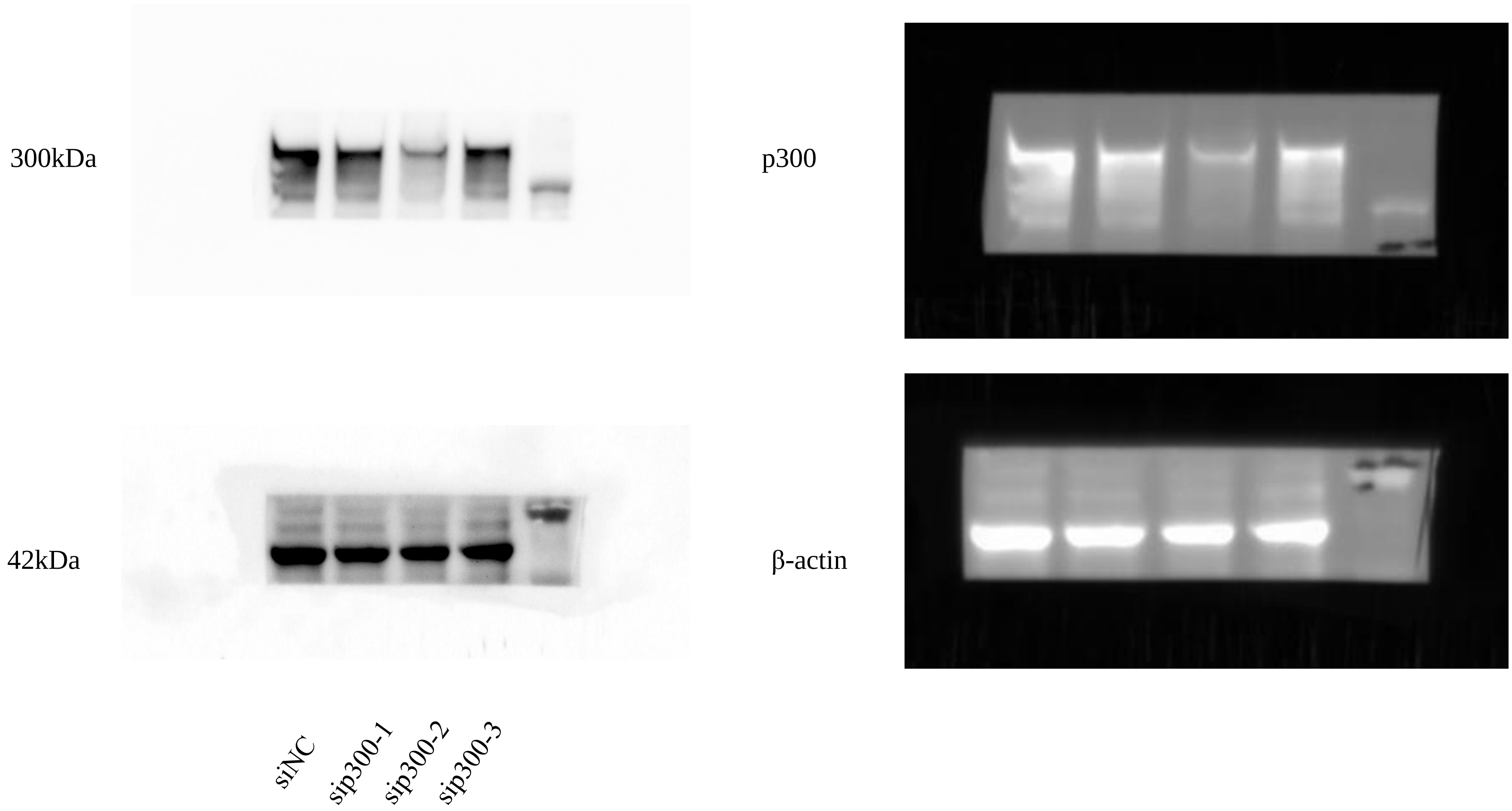

Fig 5i

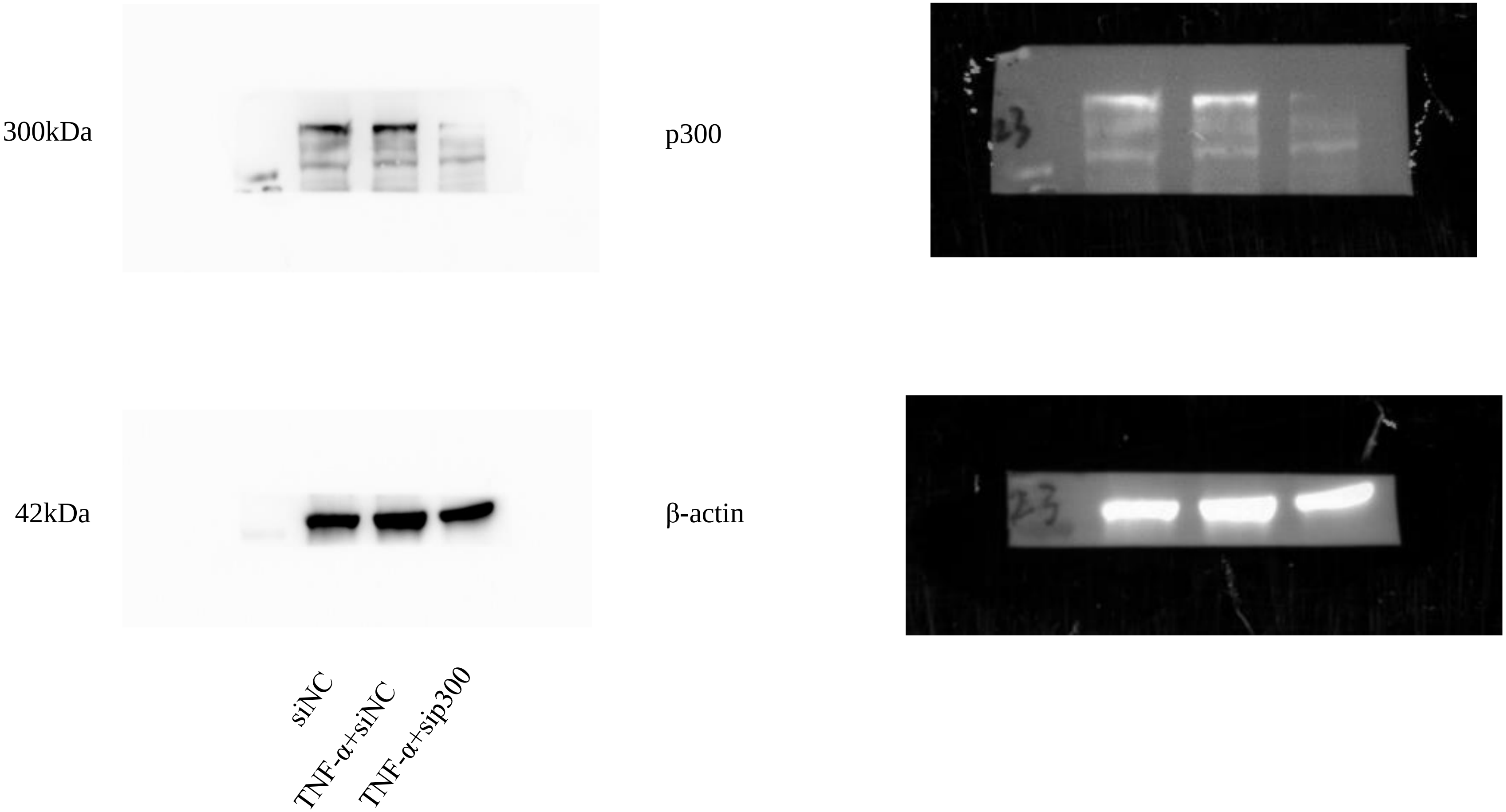

Fig 5i

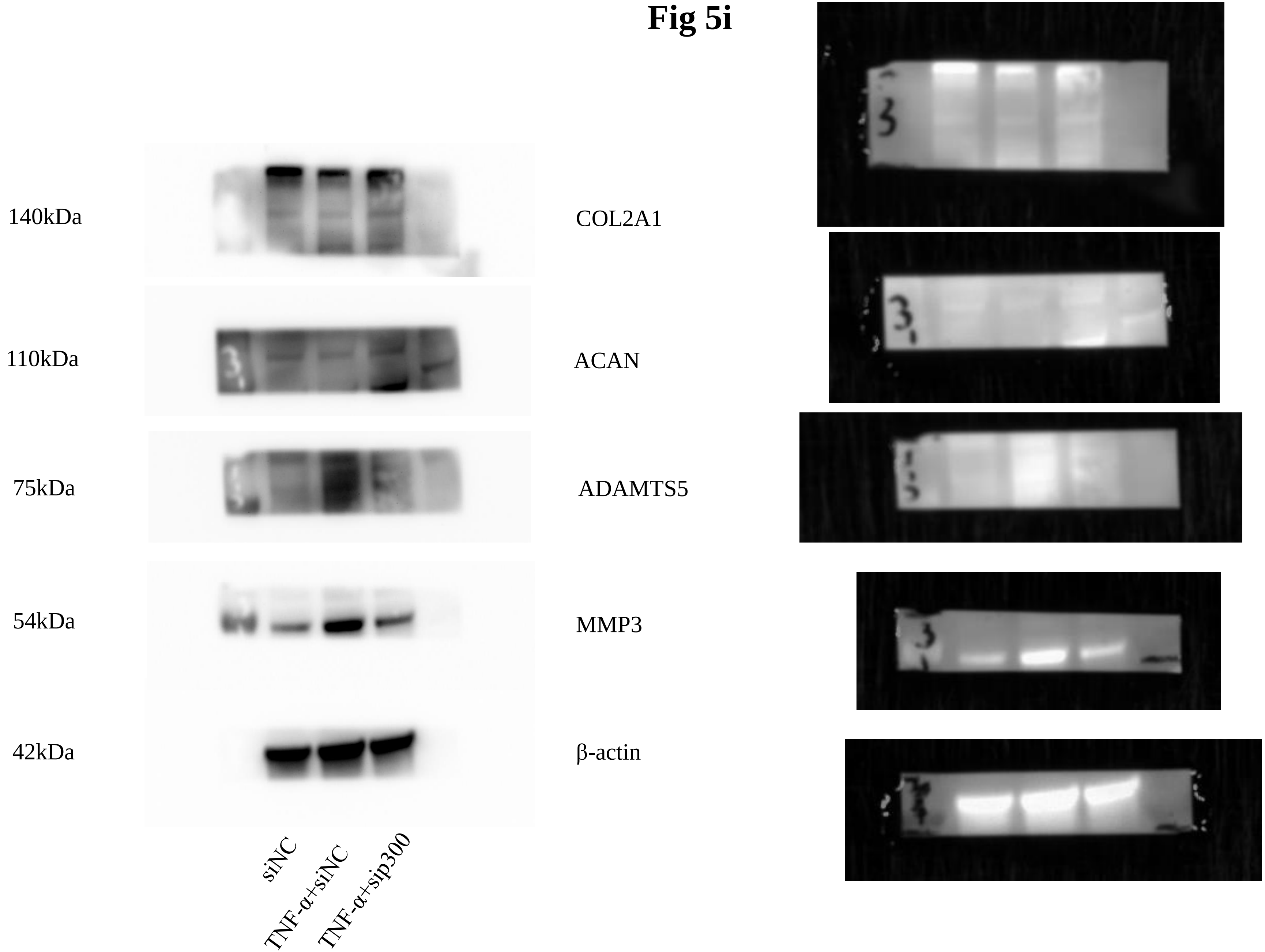

140kDa
COL2A1
110kDa
ACAN
75kDa
ADAMTS5
54kDa
MMP3
42kDa
β-actin
siNC
TNF-α+siNC
TNF-α+sip300

Fig 5j

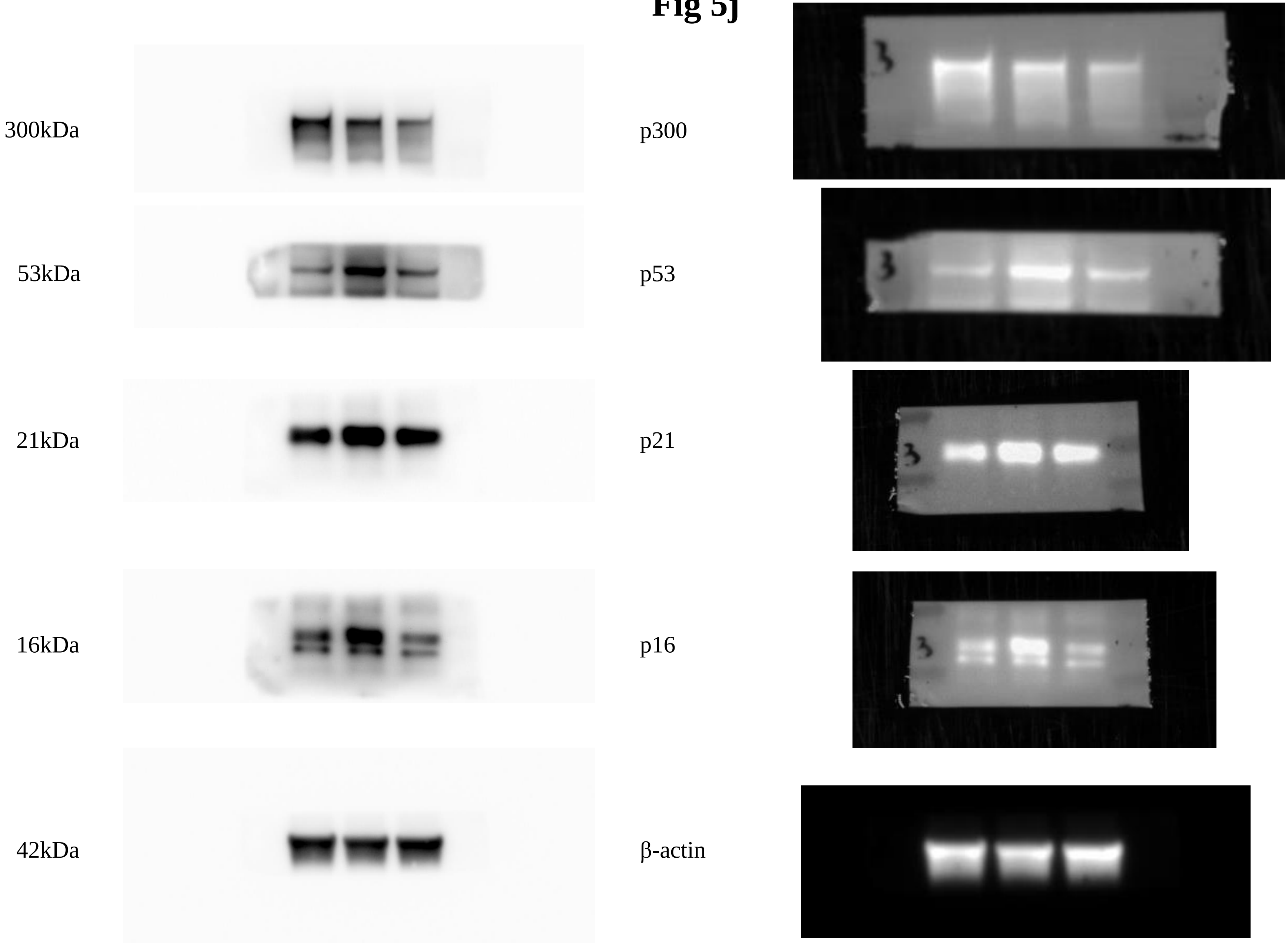

Fig 5k

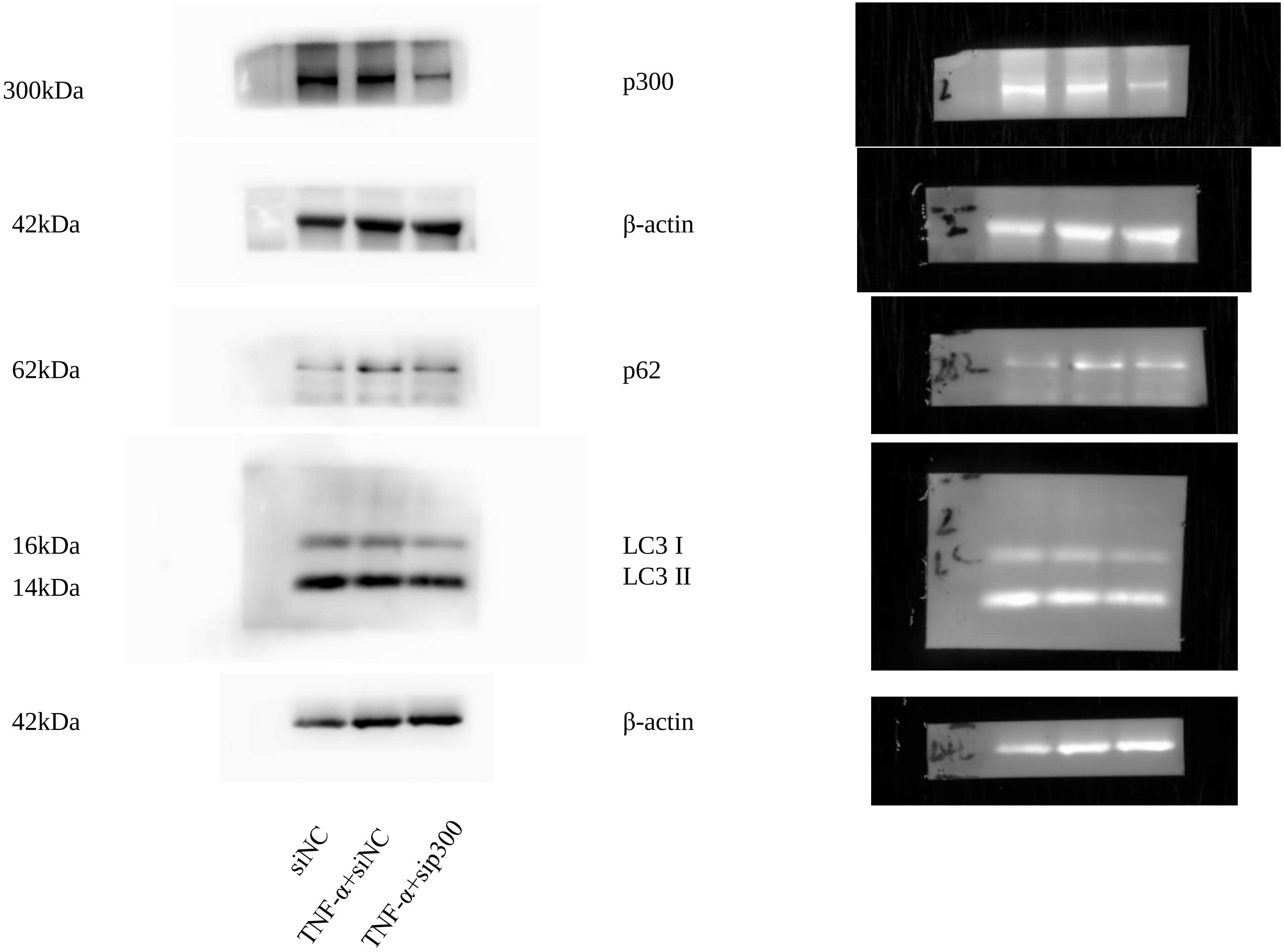
300kDa
p300
42kDa
β-actin
62kDa
p62
16kDa
LC3 I
14kDa
LC3 II
42kDa
β-actin
siNC
TNF-α+siNC
TNF-α+sip300

Fig 6b

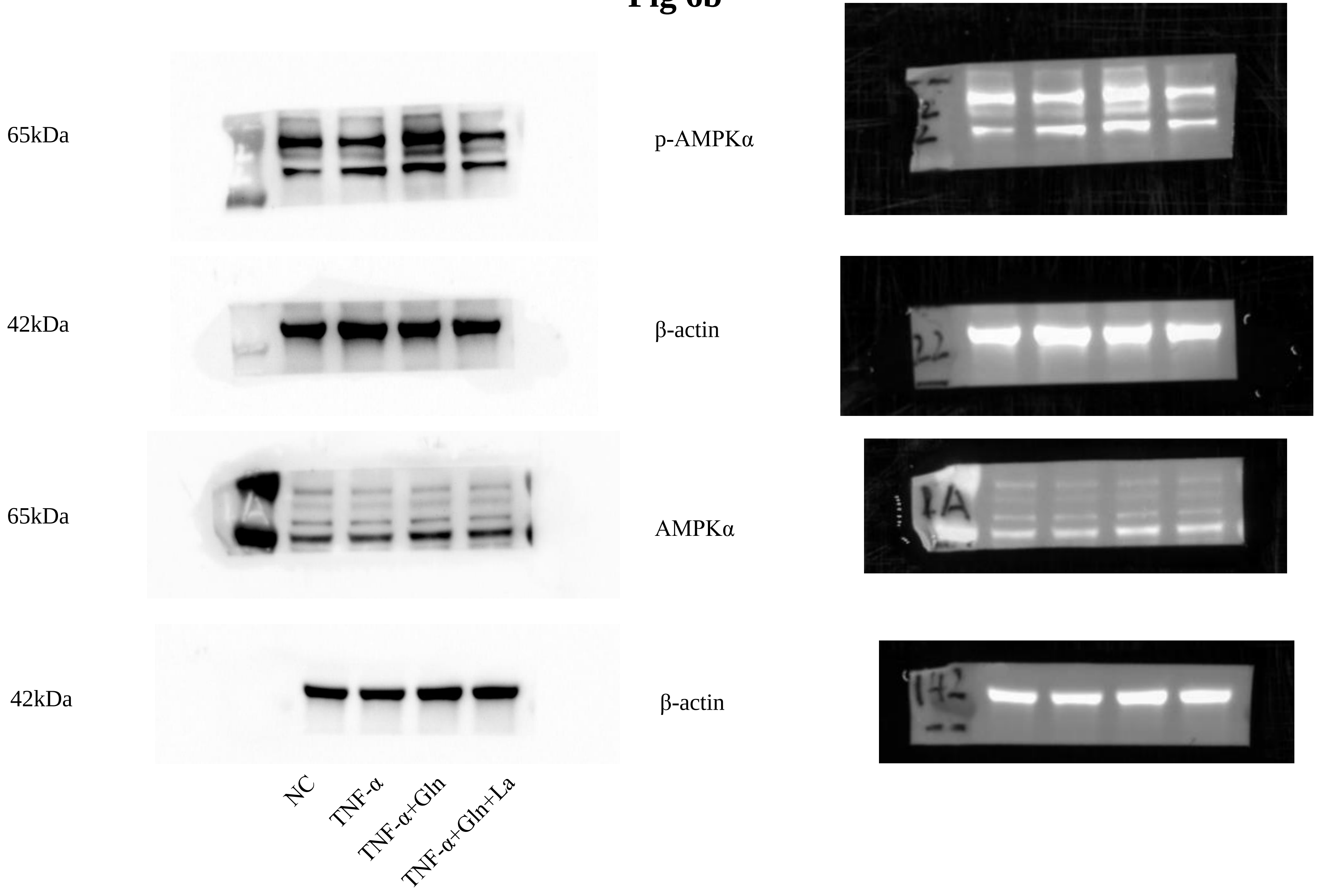
65kDa
p-AMPKα
42kDa
β-actin
65kDa
AMPKα
42kDa
β-actin
NC
TNF-α
TNF-α+Gln
TNF-α+Gln+La

Fig 7a

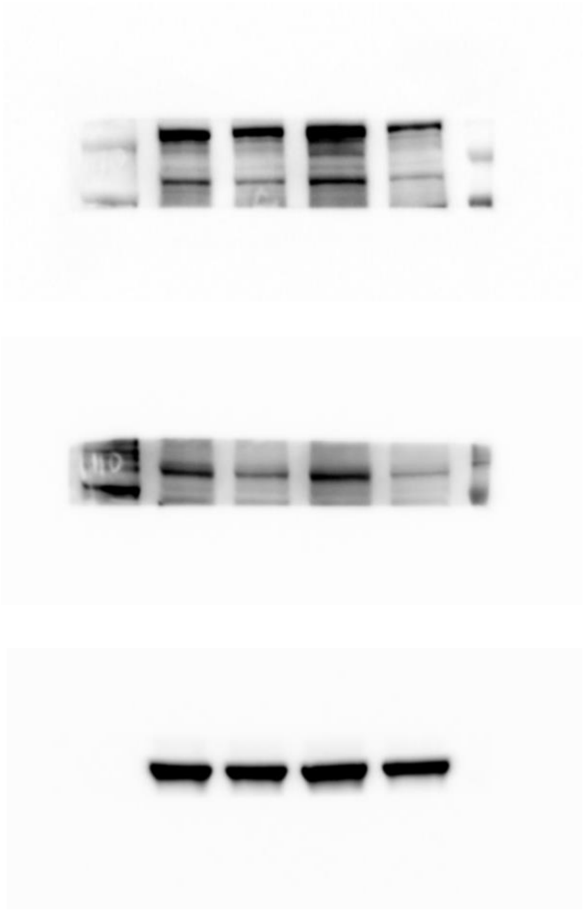

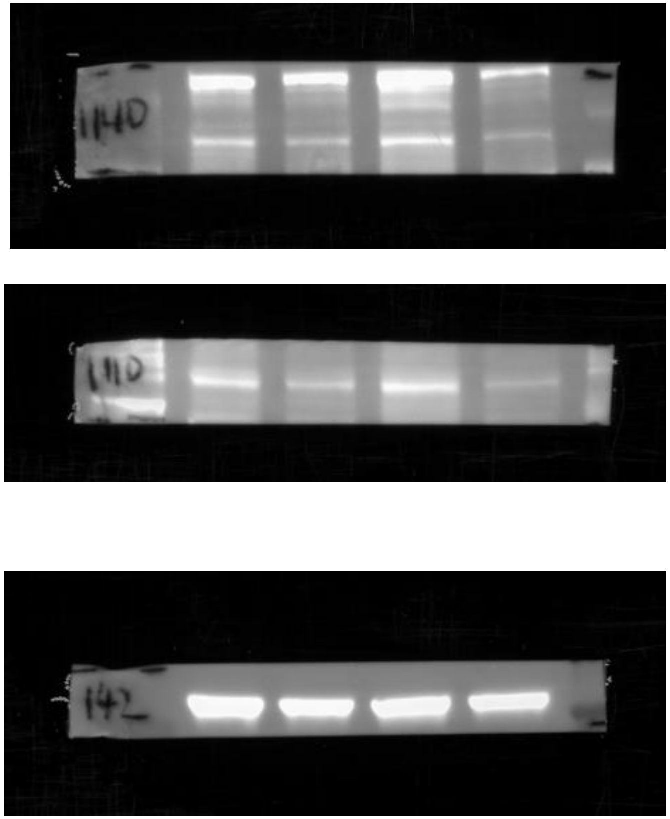

140kDa COL2A1

110kDa ACAN

42kDa β-actin

NC
TNF-α
TNF-α+Gln
TNF-α+Gln+ComC

Fig 7a

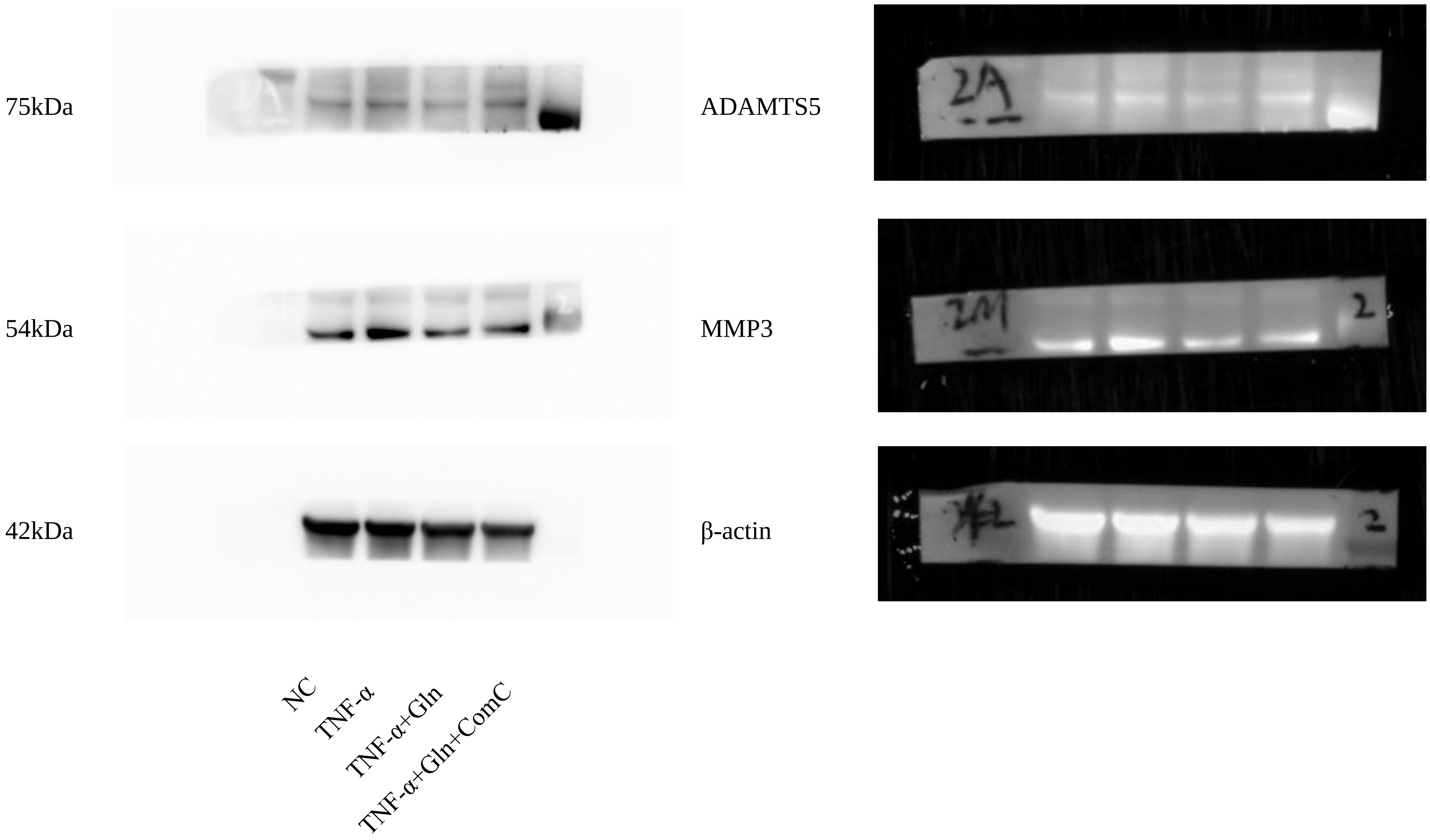

75kDa
ADAMTS5
54kDa
MMP3
42kDa
β-actin
NC
TNF-α
TNF-α+Gln
TNF-α+Gln+ComC

Fig 7c

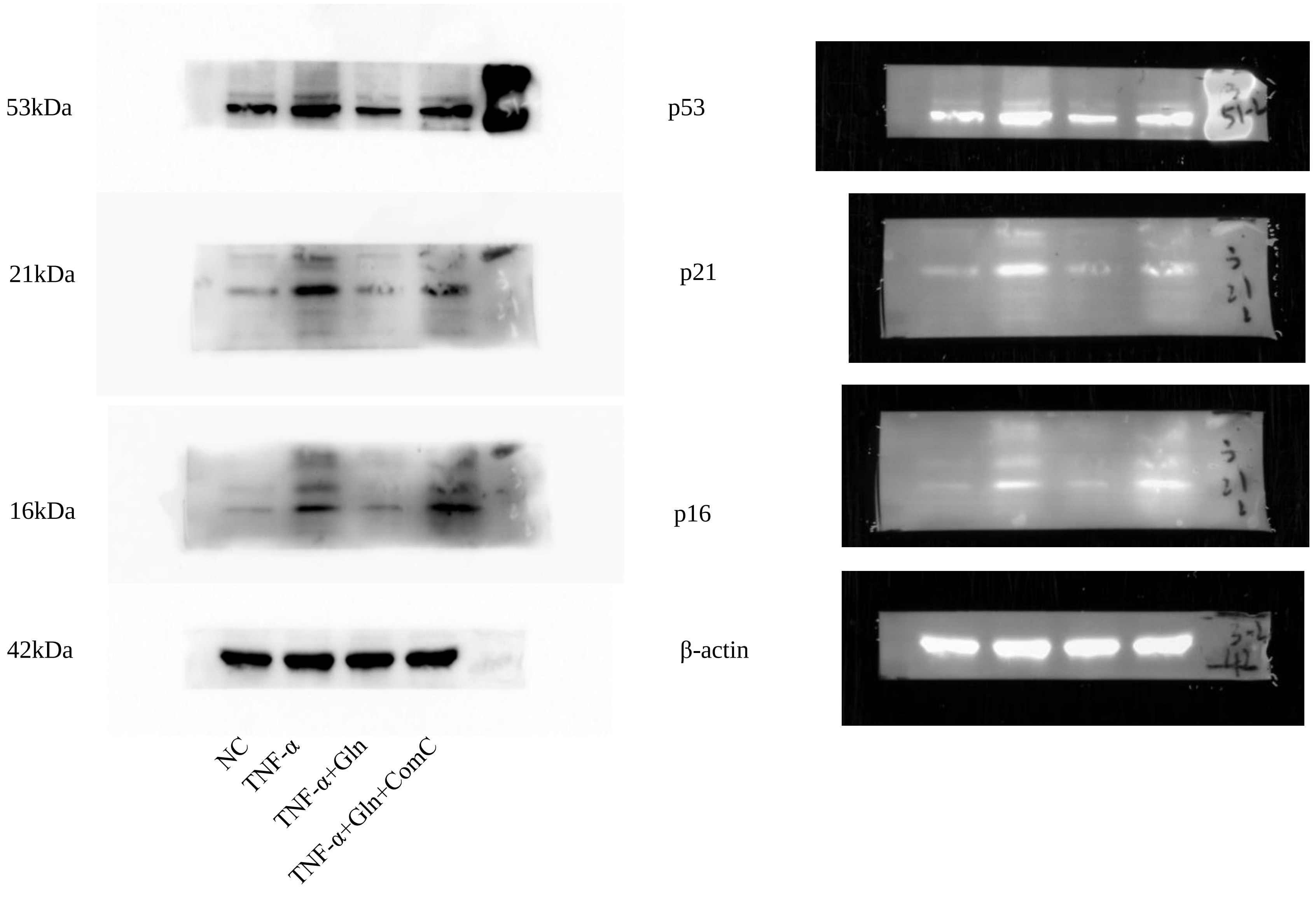
53kDa
p53
21kDa
p21
16kDa
p16
42kDa
β-actin
NC
TNF-α
TNF-α+Gln
TNF-α+Gln+ComC

Fig 7h

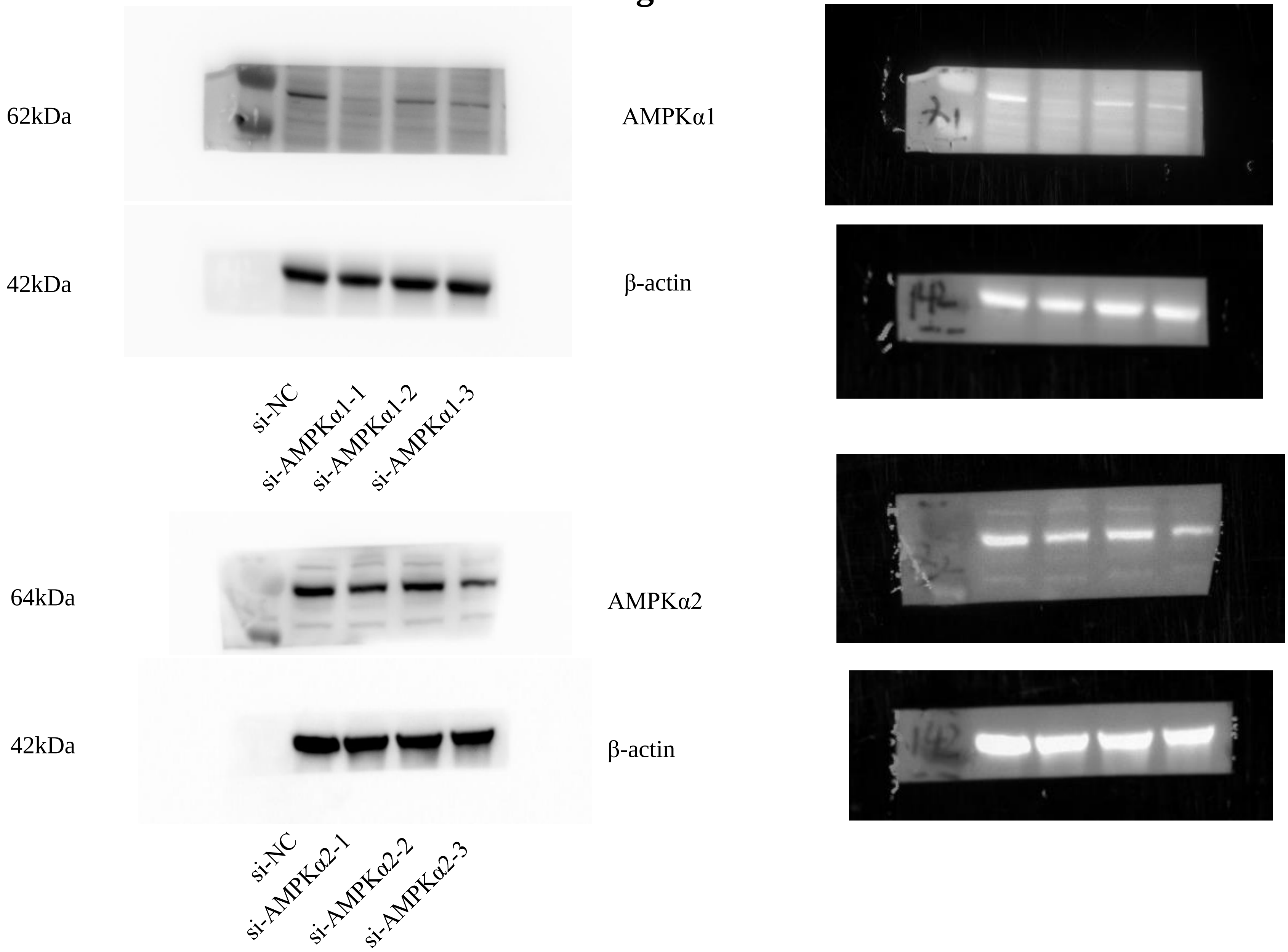
62kDa
AMPKα1
42kDa
β-actin
si-NC
si-AMPKα1-1
si-AMPKα1-2
si-AMPKα1-3
64kDa
AMPKα2
42kDa
β-actin
si-NC
si-AMPKα2-1
si-AMPKα2-2
si-AMPKα2-3

Fig 7i

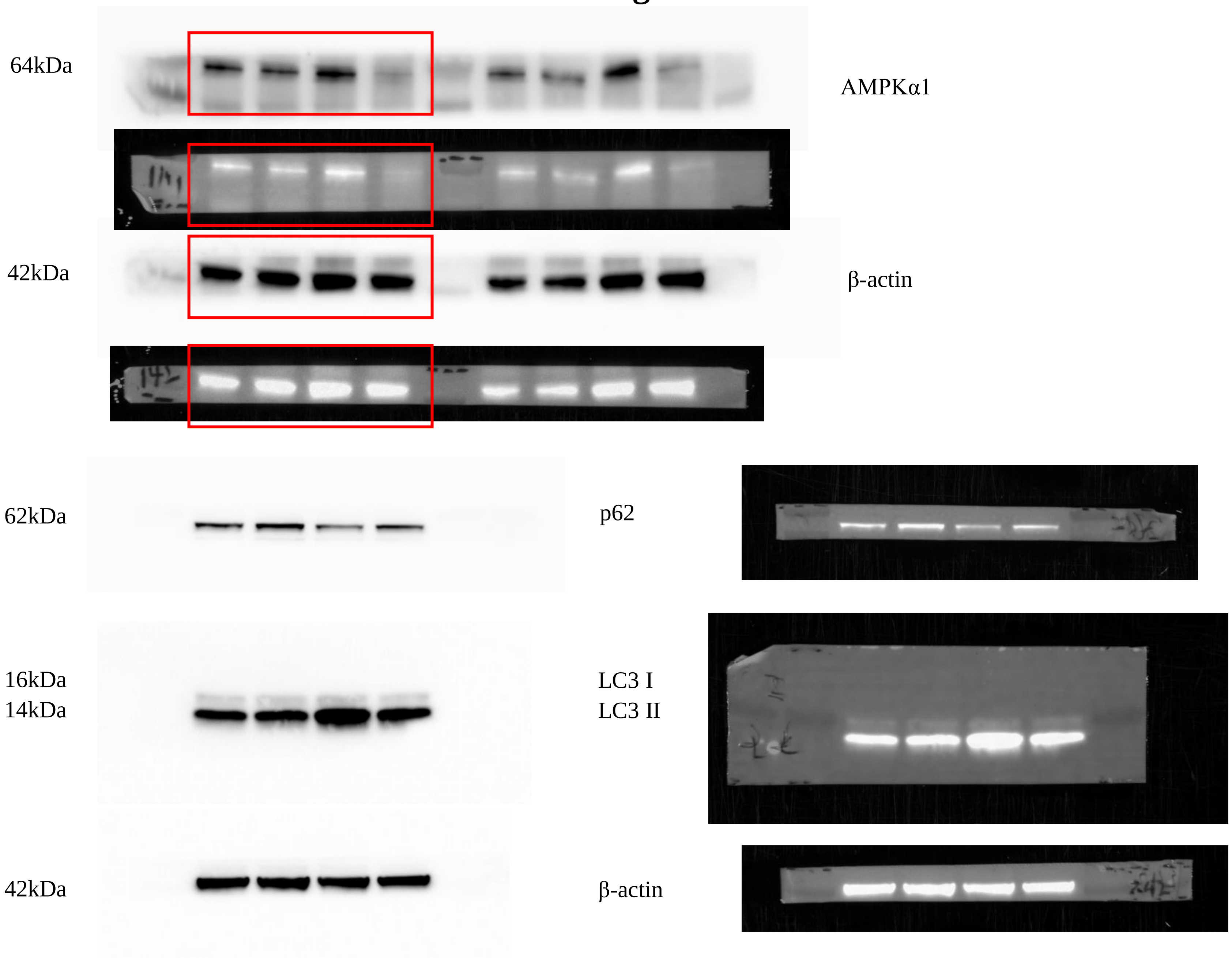
64kDa
AMPKα1
42kDa
β-actin
62kDa
p62
16kDa
14kDa
LC3 I
LC3 II
42kDa
β-actin

Fig 7j

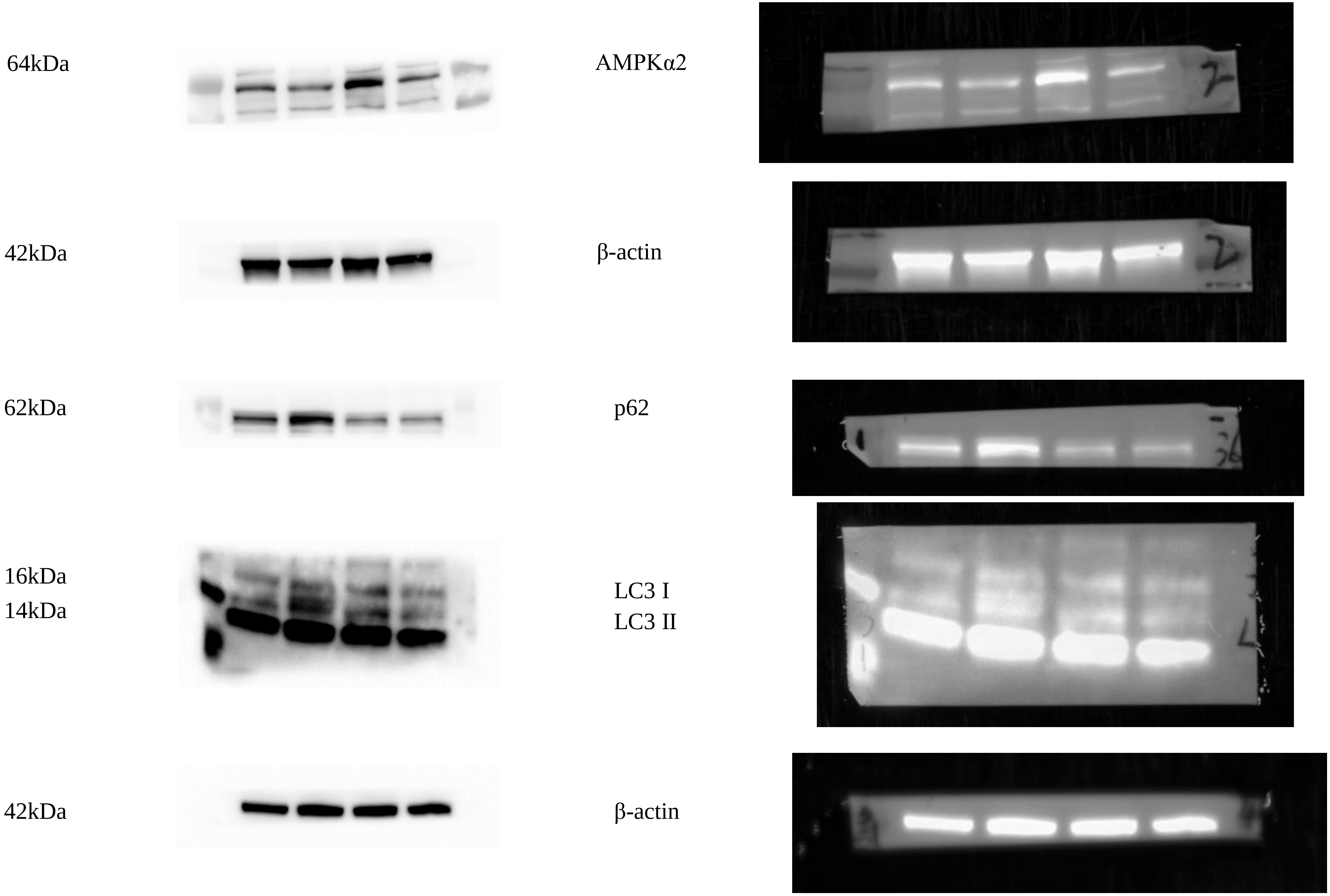

64kDa
AMPKα2
42kDa
β-actin
62kDa
p62
16kDa
14kDa
LC3 I
LC3 II
42kDa
β-actin

Supplementary Figure 1a

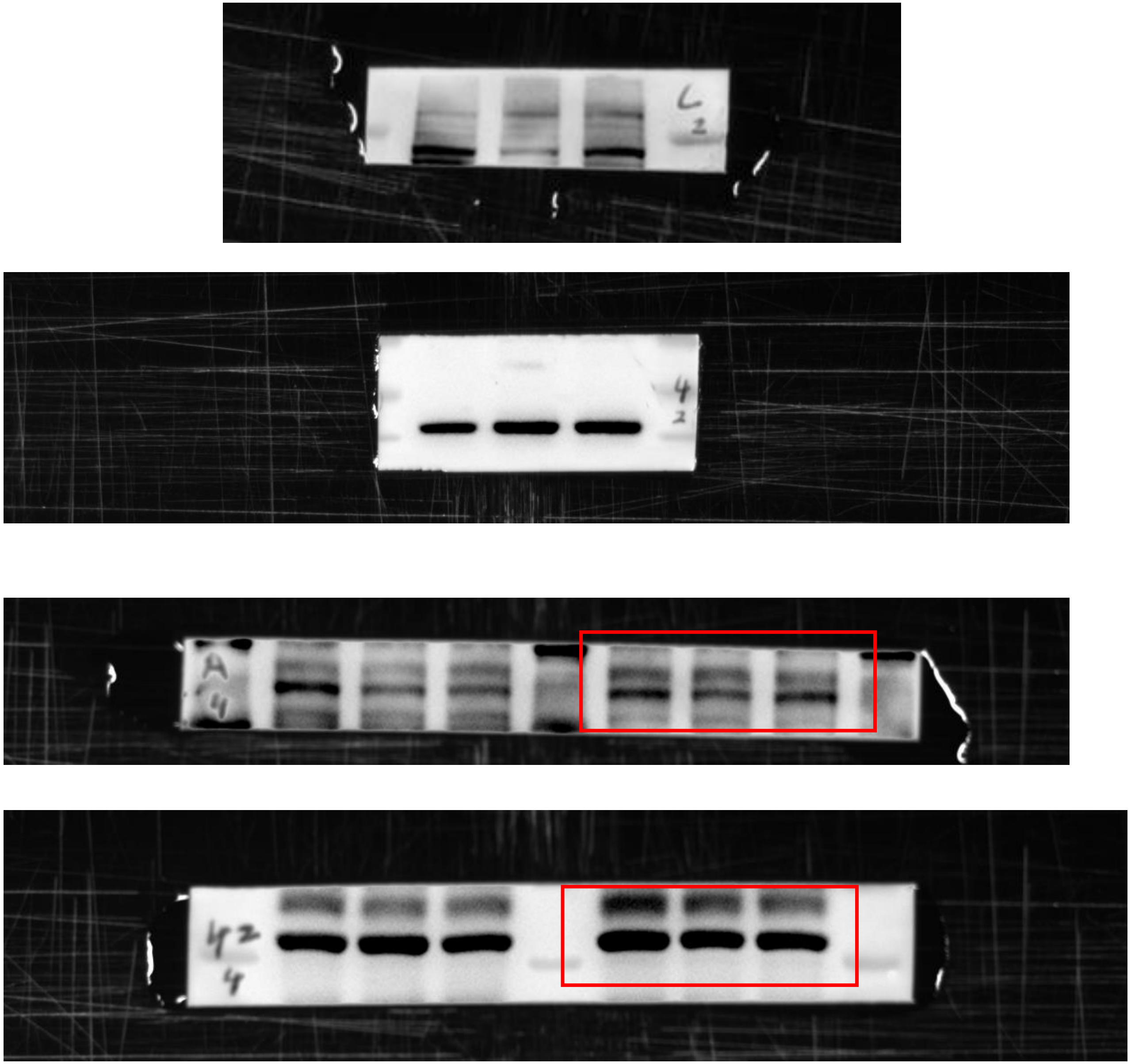

140kDa COL2A1

42kDa ACTB

110kDa ACAN

42kDa ACTB

Supplementary Figure 1a

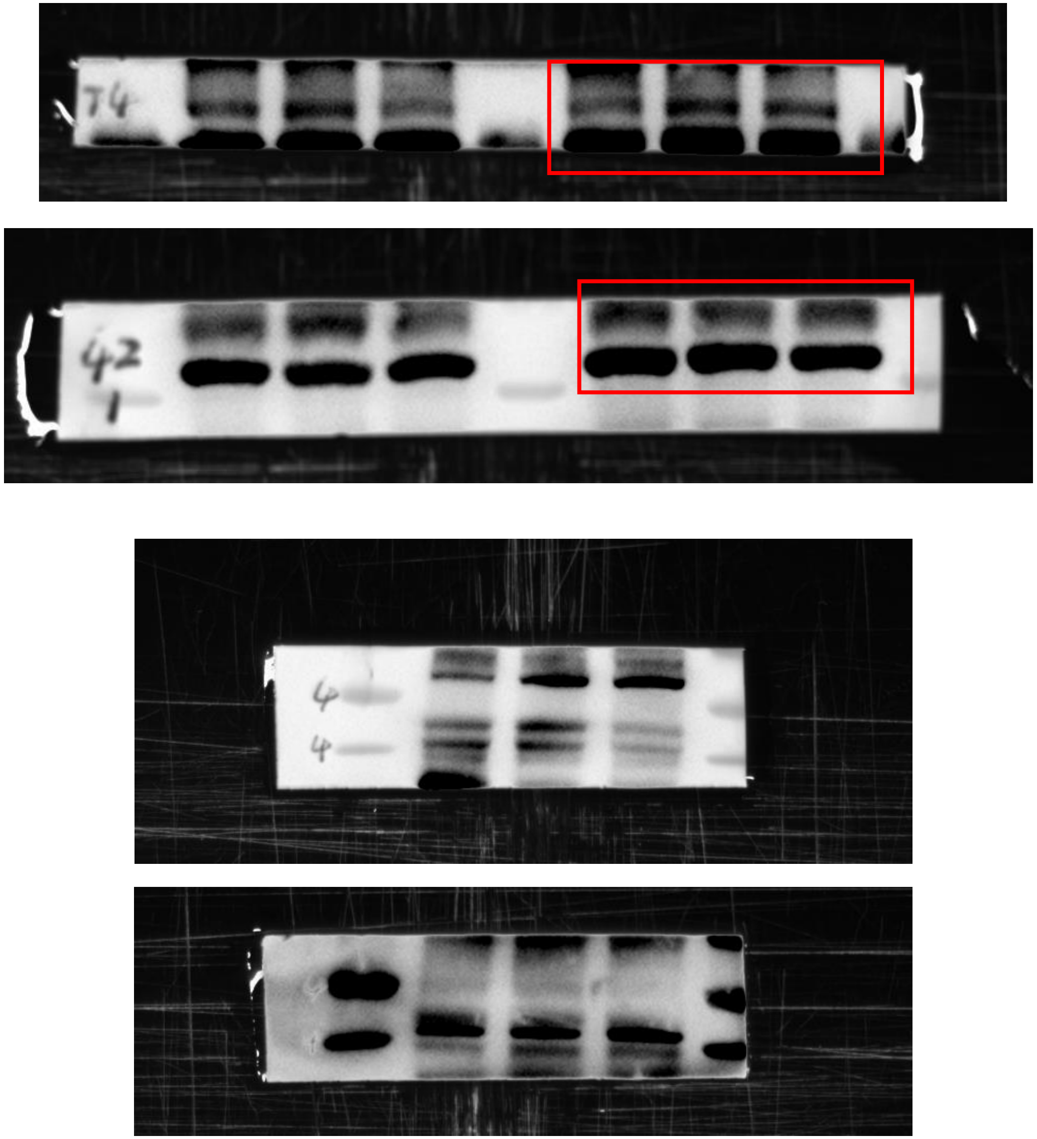

75kDa ADAMTS5

42kDa ACTB

54kDa MMP3

42kDa ACTB

Supplementary Figure 1c

21kDa

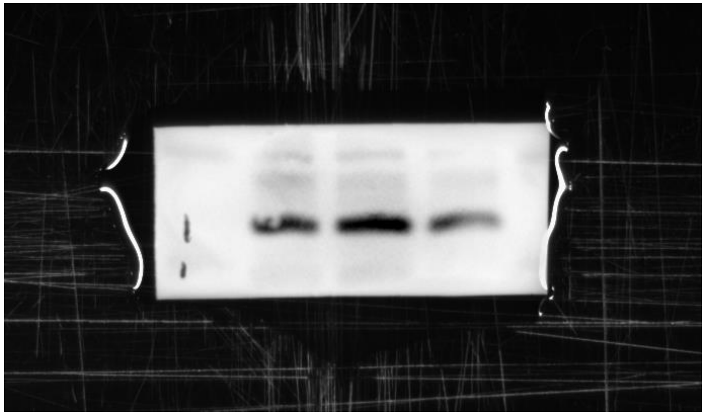

P21

42kDa

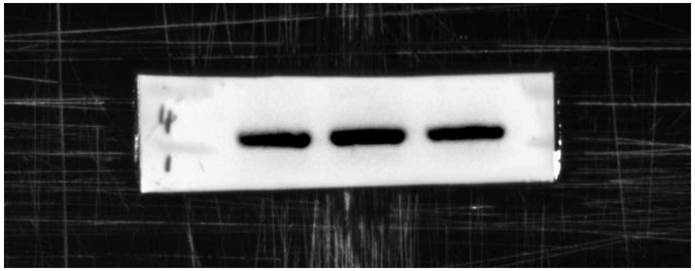

ACTB

Supplementary Figure 1e and 1g

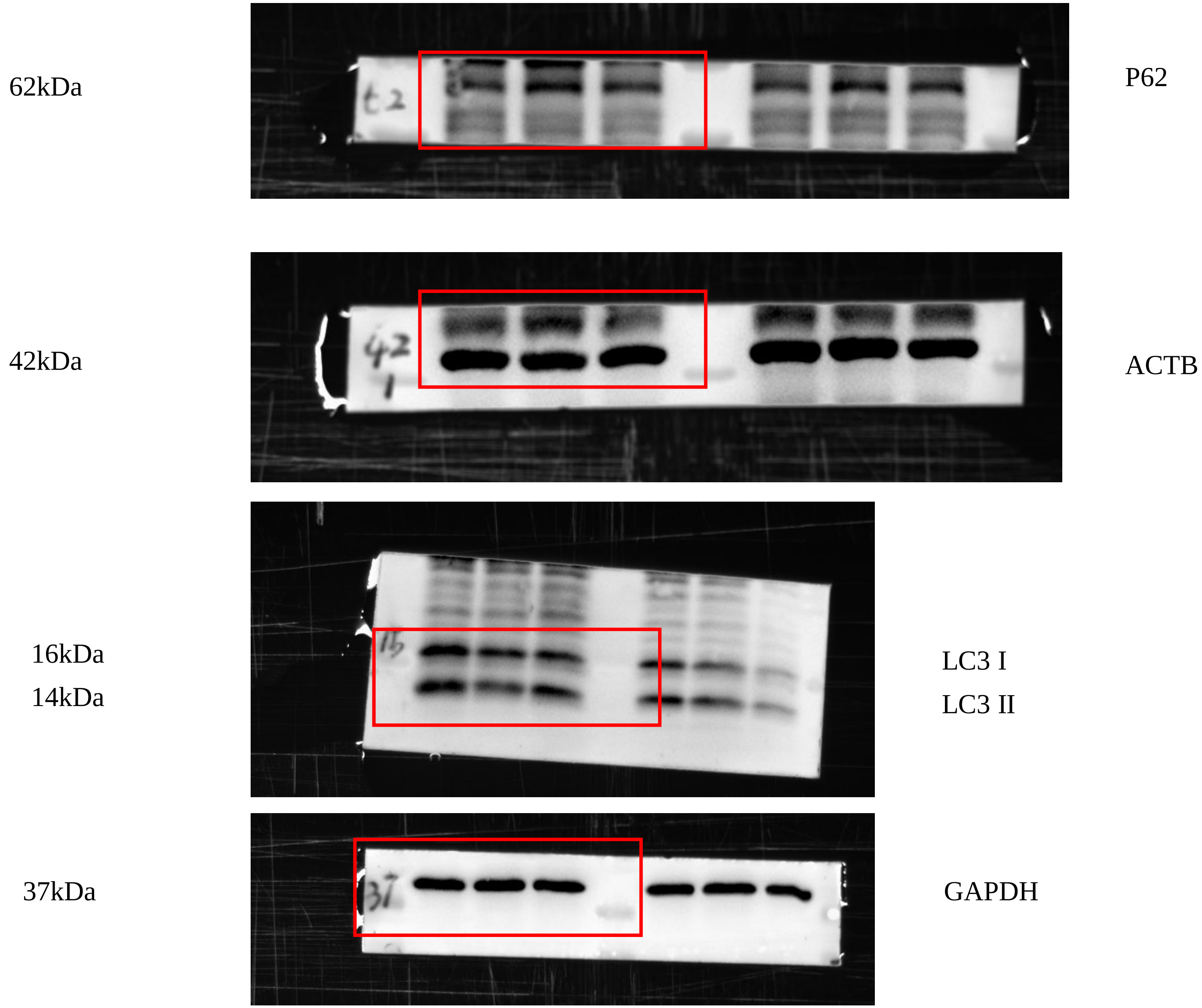

Supplementary Figure 1j

289kDa

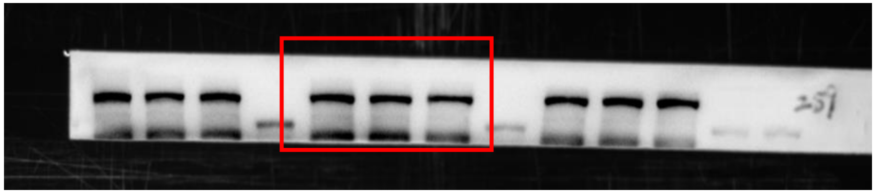

p-mTOR

42kDa

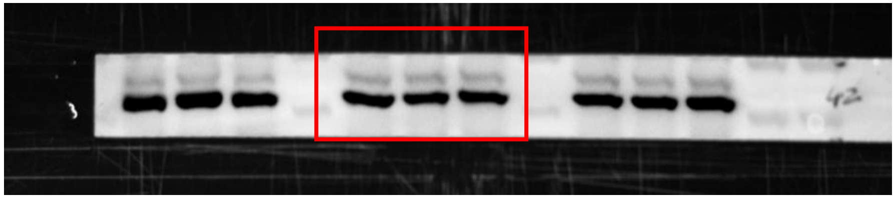

ACTB

289kDa

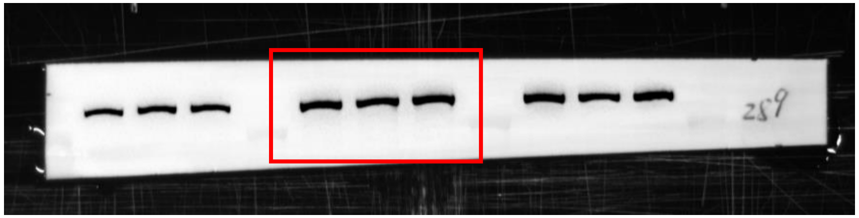

mTOR

42kDa

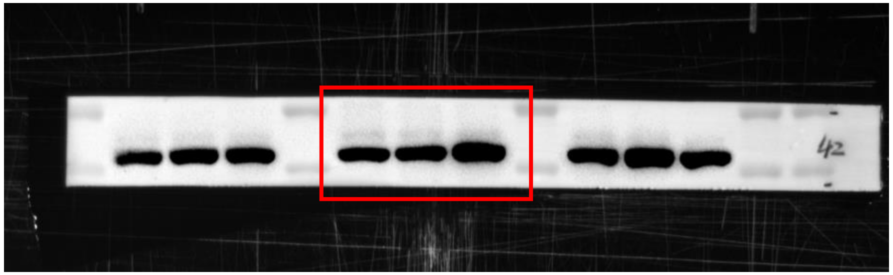

ACTB

Supplementary Table. 1. The primary antibodies for western blotting

Name	Dilution	Lot Number	Company	Country
COL2A1	1:500	YT1022	Immunoway	USA
ACAN	1:500	ab36861	Abcam	USA
ADAMTS5	1:1000	Ab41037	Abcam	USA
MMP3	1:1000	YT4465	Immunoway	USA
β-actin	1:1000	AC026	Abclonal	USA
P53	1:500	60283-2-Ig	Proteintech	USA
P21	1:1000	Ab109199	Abcam	USA
P16	1:1000	Ab51243	Abcam	USA
P62	1:1000	23214	CST	USA
LC3	1:1000	2775S	CST	USA
HK2	1:1000	22029-1-AP	Proteintech	USA
PFKP	1:1000	13389-1-AP	Proteintech	USA
PKM2	1:1000	15822-1-AP	Proteintech	USA
LDHA	1:1000	19987-1-AP	Proteintech	USA
P300	1:1000	ab275378	Abcam	USA
Kla	1:1000	PTM1401	PTM BIO	China
p-AMPKα	1:1000	2535T	CST	USA
AMPKα	1:1000	2532S	CST	USA
AMPKα1	1:1000	ET1608-40	HUABIO	China
AMPKα2	1:1000	HA600078	HUABIO	China
p-mTOR	1:1000	2971S	CST	USA
mTOR	1:1000	2972S	CST	USA
GAPDH	1:1000	YN5585	Immunoway	USA
Rabbit IgG	-	2729s	CST	USA

Supplementary Table. 2. The sequence of siRNA for knockdown

siRNA		Sequence (5'-3')
siRNA-NC	Forward strand	UUCUCCGAACGUGUCACGUdTdT
	Reverse strand	ACGUGACACGUUCGGAGAAdTdT
siRNA-p300	Forward strand	GGACUACCCUAUCAAGUAAACdTdT
	Reverse strand	GUUUACUUGAUAGGGUAGUCCdTdT
siRNA-AMPKα1	Forward strand	CGAGUUGACUGGACAUAAAdTdT
	Reverse strand	UUUAUGUCCAGUCAACUCGdTdT
siRNA-AMPKα2	Forward strand	CCAGAUGAAUGCUAAGAUAdTdT
	Reverse strand	UAUCUUAGCAUUCAUCUGGdTdT

Supplementary Table. 3. The primary antibodies for immunohistochemical staining

Name	Dilution	Lot Number	Company	Country
ACAN	1:100	A8536	Abclonal	USA
MMP3	1:100	YT4465	Immunoway	USA
P16	1:100	ab241543	Abcam	USA
LC3	1:100	14600-1-AP	Proteintech	USA
HK2	1:100	22029-1-AP	Proteintech	USA
PFKP	1:100	13389-1-AP	Proteintech	USA
PKM2	1:100	15822-1-AP	Proteintech	USA
LDHA	1:100	19987-1-AP	Proteintech	USA
P300	1:100	A13016	Abclonal	USA
Kla	1:100	PTM1401	PTM BIO	China
p-AMPKα	1:100	YP0575	Immunoway	USA
AMPKα	1:100	YT0216	Immunoway	USA

Supplementary Table. 4. The list of DEGs for all pathways

symbol	pvalue	padj	pathway_id	pathway_name
INSIG1	3.80E-13	1.43E-08		
MVD	1.85E-11	3.48E-07	hsa00900;hsa01100	Terpenoid backbone biosynthesis ;Metabolic pathways
ACAT2	6.09E-11	5.73E-07	hsa00071;hsa00280;hsa00310;hsa00380;hsa00620;hsa00630;hsa00650;hsa00900;hsa01100;hsa01200;hsa01212;hsa04975	Fatty acid degradation ;Valine, leucine and isoleucine degradation ;Lysine degradation ;Tryptophan metabolism ;Pyruvate metabolism ;Glyoxylate and dicarboxylate metabolism ;Butanoate metabolism ;Terpenoid backbone biosynthesis ;Metabolic pathways ;Carbon metabolism ;Fatty acid metabolism ;Fat digestion and absorption
TMEM97	5.29E-11	5.73E-07		
FDPS	8.01E-11	6.04E-07	hsa00900;hsa01100;hsa05164;hsa05166	Terpenoid backbone biosynthesis ;Metabolic pathways ;Influenza A ;Human T-cell leukemia virus 1 infection
MSMO1	4.00E-10	2.51E-06	hsa00100;hsa01100	Steroid biosynthesis ;Metabolic pathways
DHCR7	2.42E-09	1.30E-05	hsa00100;hsa01100	Steroid biosynthesis ;Metabolic pathways
FDFT1	1.02E-08	4.82E-05	hsa00100;hsa01100	Steroid biosynthesis ;Metabolic pathways
IDI1	2.16E-08	9.04E-05	hsa00900;hsa01100	Terpenoid backbone biosynthesis ;Metabolic pathways
LPIN1	4.05E-08	0.000139	hsa00561;hsa00564;hsa01100;hsa04150	Glycerolipid metabolism ;Glycerophospholipid metabolism ;Metabolic pathways ;mTOR signaling pathway
ACSS2	3.75E-08	0.000139	hsa00010;hsa00620;hsa00630;hsa00640;hsa01100;hsa01200	Glycolysis / Gluconeogenesis ;Pyruvate metabolism ;Glyoxylate and dicarboxylate metabolism ;Propanoate metabolism ;Metabolic pathways ;Carbon metabolism
SCD	5.07E-08	0.000159	hsa01040;hsa01100;hsa01212;hsa03320;hsa04152	Biosynthesis of unsaturated fatty acids ;Metabolic pathways ;Fatty acid metabolism ;PPAR signaling pathway ;AMPK signaling pathway
SQLE	7.94E-08	0.00023	hsa00100;hsa01100	Steroid biosynthesis ;Metabolic pathways
HMGCS1	1.68E-07	0.000428	hsa00280;hsa00650;hsa00900;hsa01100;hsa03320	Valine, leucine and isoleucine degradation ;Butanoate metabolism ;Terpenoid backbone biosynthesis ;Metabolic pathways ;PPAR signaling pathway

FADS2	1.71E-07	0.000428	hsa00592;hsa01040;hsa01100;hsa01212;hsa03320	alpha-Linolenic acid metabolism ;Biosynthesis of unsaturated fatty acids ;Metabolic pathways ;Fatty acid metabolism ;PPAR signaling pathway
LSS	4.00E-07	0.000941	hsa00100;hsa01100	Steroid biosynthesis ;Metabolic pathways
PCSK9	5.46E-07	0.00121	hsa04979	Cholesterol metabolism
NUPR1	8.43E-07	0.001764	hsa05202	Transcriptional misregulation in cancer
ERG28	1.01E-06	0.001997		
DHCR24	1.66E-06	0.003123	hsa00100;hsa01100	Steroid biosynthesis ;Metabolic pathways
LDLR	3.86E-06	0.006924	hsa04144;hsa04913;hsa04925;hsa04927;hsa04934;hsa04976;hsa04979;hsa05145;hsa05160;hsa05417	Endocytosis ;Ovarian steroidogenesis ;Aldosterone synthesis and secretion ;Cortisol synthesis and secretion ;Cushing syndrome ;Bile secretion ;Cholesterol metabolism ;Toxoplasmosis ;Hepatitis C ;Lipid and atherosclerosis
HSD17B7	4.52E-06	0.007421	hsa00100;hsa00140;hsa01100;hsa04913	Steroid biosynthesis ;Steroid hormone biosynthesis ;Metabolic pathways ;Ovarian steroidogenesis
HMGCR	4.53E-06	0.007421	hsa00900;hsa01100;hsa04152;hsa04976	Terpenoid backbone biosynthesis ;Metabolic pathways ;AMPK signaling pathway ;Bile secretion
MMAB	5.36E-06	0.008407	hsa00860;hsa01100;hsa01240	Porphyrin and chlorophyll metabolism ;Metabolic pathways ;Biosynthesis of cofactors
SNRPGP10	5.67E-06	0.008539		
CLCN6	8.91E-06	0.012907		
FASN	1.74E-05	0.024287	hsa00061;hsa01100;hsa01212;hsa04152;hsa04910	Fatty acid biosynthesis ;Metabolic pathways ;Fatty acid metabolism ;AMPK signaling pathway ;Insulin signaling pathway
CCRL2	2.42E-05	0.032021		
POM121L9P	2.47E-05	0.032021		
PCYT2	3.03E-05	0.038067	hsa00440;hsa00564;hsa01100	Phosphonate and phosphinate metabolism ;Glycerophospholipid metabolism ;Metabolic pathways

ALDH1B1	3.70E-05	0.044954	hsa00010;hsa00053;hsa00071;hsa00280;hsa00310;hsa00330;hsa00340;hsa00380;hsa00410;hsa00561;hsa00620;hsa00770;hsa01100;hsa01240	Glycolysis / Gluconeogenesis ;Ascorbate and aldarate metabolism ;Fatty acid degradation ;Valine, leucine and isoleucine degradation ;Lysine degradation ;Arginine and proline metabolism ;Histidine metabolism ;Tryptophan metabolism ;beta-Alanine metabolism ;Glycerolipid metabolism ;Pyruvate metabolism ;Pantothenate and CoA biosynthesis ;Metabolic pathways ;Biosynthesis of cofactors
SC5D	3.86E-05	0.045408	hsa00100;hsa01100	Steroid biosynthesis ;Metabolic pathways
CYP51A1	4.36E-05	0.04974	hsa00100;hsa01100	Steroid biosynthesis ;Metabolic pathways
