# Supplementary material for: Glutamine suppresses senescence and promotes autophagy through glycolysis inhibition-mediated AMPKα lactylation in intervertebral disc degeneration
Source: Commun Biol. 2024 Mar 14;7:325. doi: 10.1038/s42003-024-06000-3 (PMC10940657; doi:10.1038/s42003-024-06000-3)
Supplement: Supplementary file 2 — Description of Additional Supplementary Files [file 42003_2024_6000_MOESM2_ESM.pdf]

Description of Additional Supplementary Files

File name: Supplementary Data
Description: The source data behind the graphs in the manuscript.
